# Synthesis and Reactivity of Martin’s Spirosilane-Derived Chloromethylsilicate

**DOI:** 10.3390/molecules27061767

**Published:** 2022-03-08

**Authors:** Thomas Deis, Jérémy Forte, Louis Fensterbank, Gilles Lemière

**Affiliations:** CNRS, Institut Parisien de Chimie Moléculaire, IPCM, Sorbonne Université, 4 Place Jussieu, F-75005 Paris, France; thomas.deis@sorbonne-universite.fr (T.D.); jeremy.forte@sorbonne-universite.fr (J.F.)

**Keywords:** silicon, spirosilane, pentacoordination, Sila-Matteson rearrangement, DFT calculations

## Abstract

Pentacoordinate silicon derivatives with a chloromethyl ligand are versatile compounds that are usually obtained from the corresponding tetravalent trialkoxy- or trihalogeno(chloromethyl)silane. We describe herein the synthesis of a chloromethylsilicate bearing two Martin’s ligands readily obtained by addition of in situ generated chloromethyllithium to a spirosilane. The reactivity of this new species was evaluated and it has been established that the chloride is displaced by strong nucleophiles such as alkyllithiums and (hetero)aryllithiums. In Lewis acidic conditions, the pentacoordinate silicon species rearranges through a formal insertion of a methylene into one Si–C bond, to form a new tetravalent spirosilane with a six-membered ring. The same kind of rearrangement can be triggered also by addition of a Lewis base. The mechanism of the rearrangement in both conditions has been studied by means of DFT calculations.

## 1. Introduction

Conversely to carbon, silicon can easily reach coordination higher than four [1]. The stability of the corresponding species depends on the ligands surrounding the silicon atom and to date, an impressive number of various stable pentacoordinate and hexacoordinate silicon(IV) compounds have been reported [2,3]. Among these structures, some of them present a chloromethyl ligand that can bring peculiar reactivity and properties to the hypercoordinate system. For example, chloromethyl (bis-catecholato)silicate can be efficiently used in photoredox catalysis as chloromethyl radical precursor [4] for the formation of cyclopropane derivatives [5,6,7] and chloromethylsilatranes have shown interesting biological activities [8].

**Figure 1:**
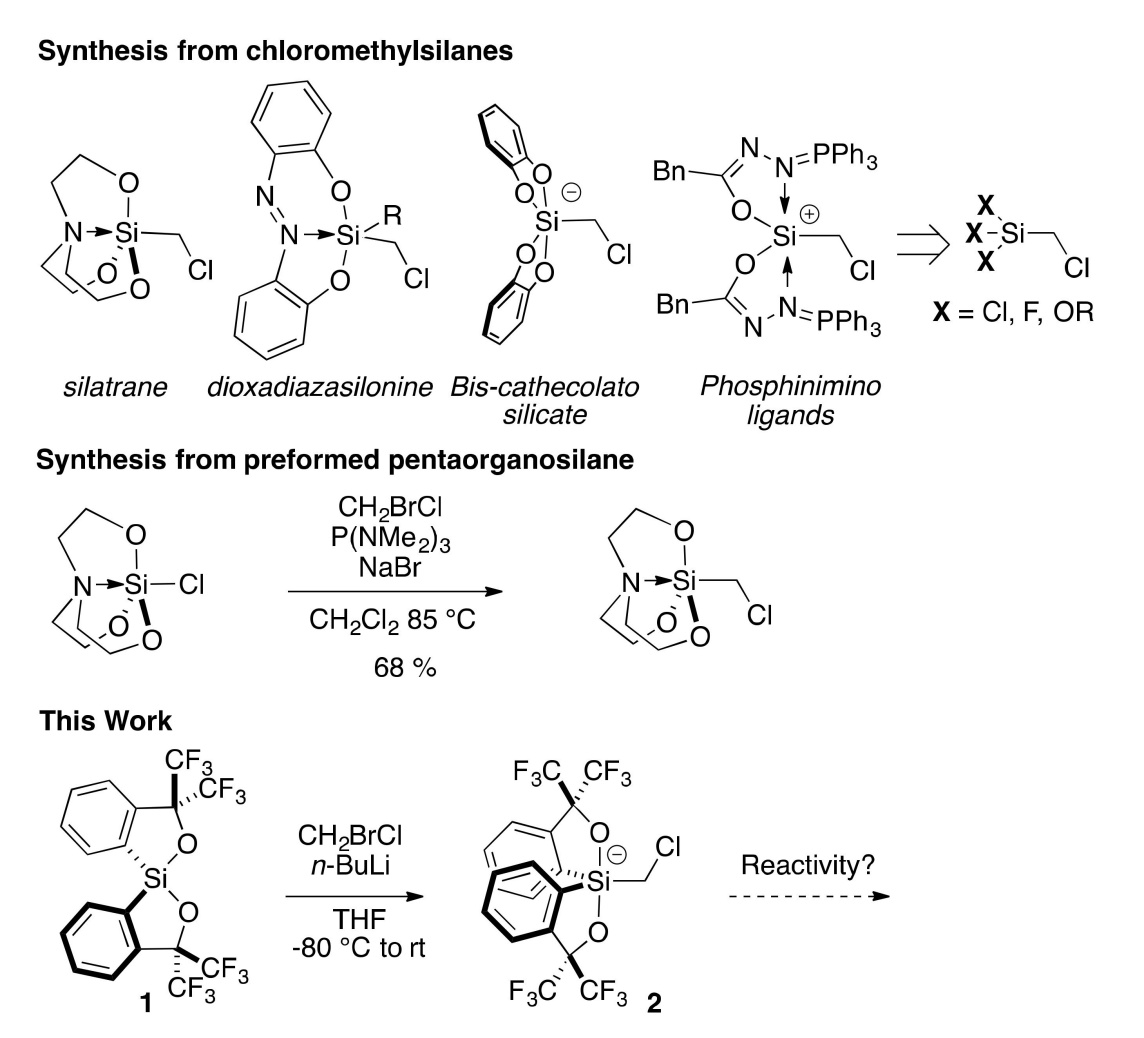
Synthesis of pentacoordinate chloromethylsilane derivatives and presentation of the objectives.

In terms of synthesis, in most cases the hypercoordinate silicon compounds featuring a chloromethyl group are obtained from the corresponding chloromethylsilane derivatives bearing three alkoxy groups or halogens on the silicon [5,6,7,8,9,10]. The direct introduction of the chloromethyl group to the silicon part has been described only once by the group of Lazareva for the formation of chloromethylsilatrane [11]. In this procedure, the chloromethyl group is introduced by mixing bromochloromethylene and a tris(amino)phosphine in the presence of a chlorosilatrane at high temperature (Figure 1).

Silicates bearing two hexafluorocumyl alkoxy ligands represent an important class of stable and isolable pentacoordinate silicon compounds [12]. They can be prepared either by the addition of a nucleophile to Lewis acidic spirosilane **1** or by addition of the dilithio derivative of hexafluorocumyl alcohol to a trichlorosilane in the case of the methyl- and phenylsilicate [13]. In recent years, our group has been interested in the synthesis of pentacoordinate silicon derivatives [14] based on Martin’s ligands in the context of Frustrated Lewis Pair chemistry, which led to new organometallic species [15,16,17]. In the present study, we wish to report the first synthesis of chloromethylsilicate **2** that can be obtained from Martin’s spirosilane **1** and its reactivity in the presence of strong nucleophiles, a Lewis acid and a Lewis base (Figure 1).

## 2. Results and Discussion

### 2.1. Synthesis of Chloromethylsilicate **2**

**Scheme 1:**
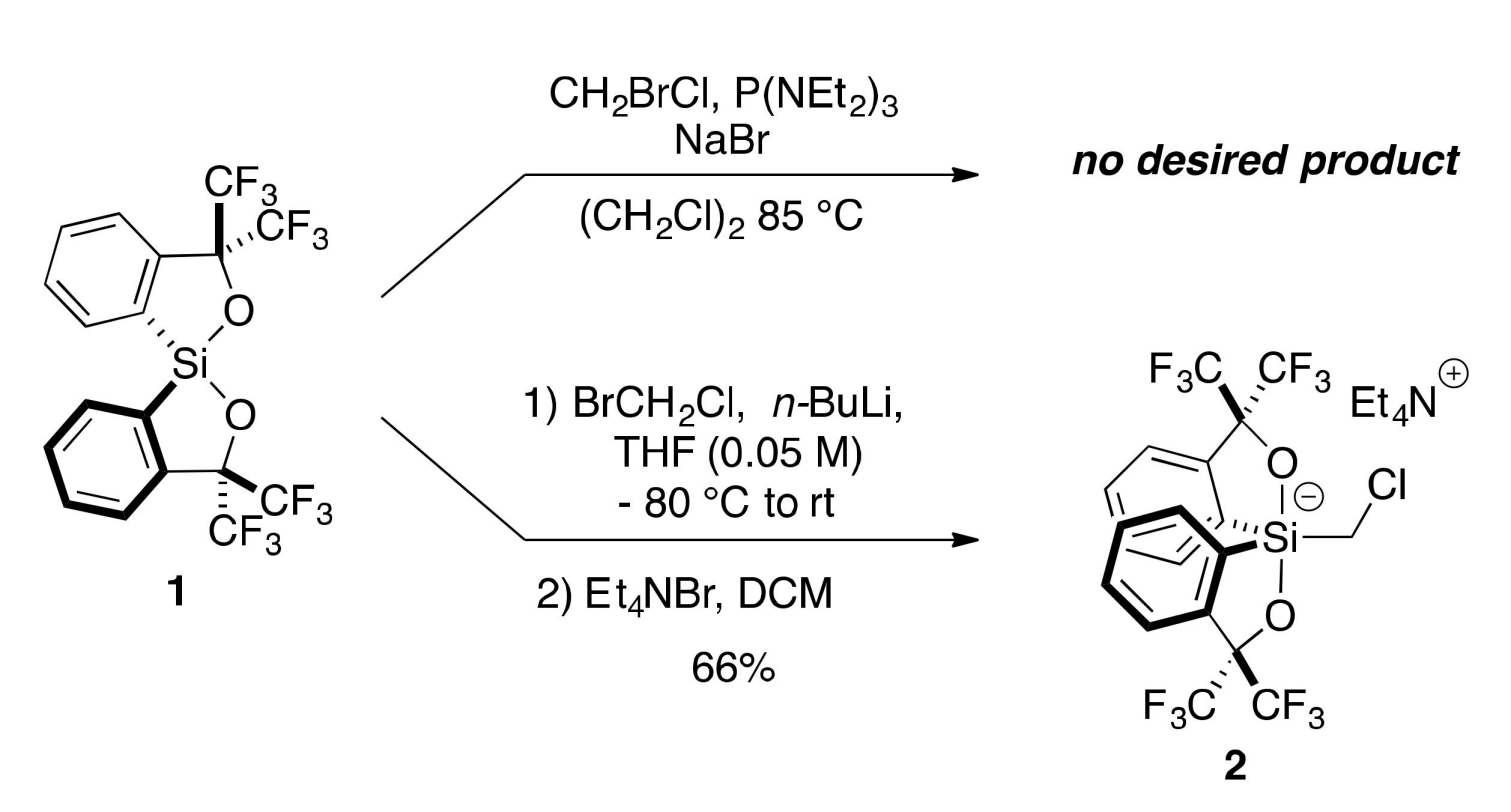
Synthesis of chloromethylsilicate **2**.

To access chloromethylsilicate **2**, we first tried to apply the conditions abovementioned in Scheme 1 for silatrane in the presence of a tris(amino)phosphine, to spirosilane **1**. Unfortunately, by following the same procedure, no desired product could be isolated, and therefore another strategy was adopted. It is well established that alkyllithium reagents can efficiently add to spirosilane **1** at low temperature to afford the corresponding pentacoordinate silicon adducts [18,19]. We wondered if chloromethyllithium, which can be generated in situ in cryogenic conditions from bromochloro methylene and *n*-butyllithium [20], would add to spirosilane **1** to form a stable chloromethyl adduct. Gratifyingly, the dropwise addition of *n*-butyllithium to a solution of spirosilane **1** and methylene bromochloride at −80 °C led to the formation of chloromethylsilicate **2** (Scheme 1). It turned out that the concentration was very important to avoid the formation of by-products and a concentration of 0.05 M was necessary to get a good yield of 66% after cation exchange (Scheme 1).

**Figure 2:**
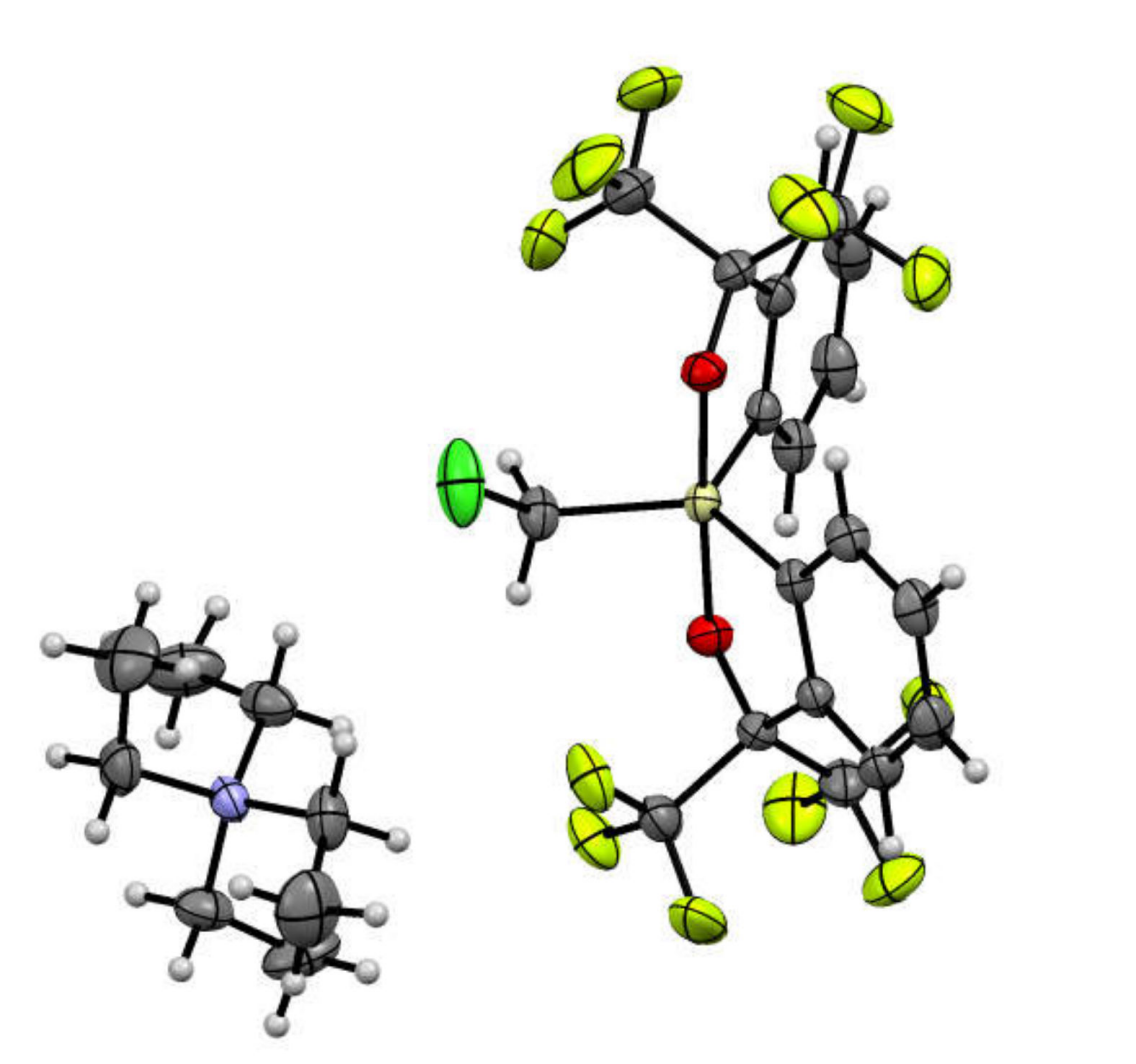
X-Ray structure of chloromethylsilicate **2** (CCDC number 2124348). Ellipsoids are shown with 50% of probability.

X-ray diffraction (XRD) analysis of crystals **2** confirmed the pentacoordination of the silicon and the presence of the chlorine atom (Figure 2). The compound belongs to the monoclinic P2_1_/c space group. As for most of the pentacoordinate structures based on Martin’s ligands, the geometry is close to a trigonal bipyramid with both oxygen atoms placed in axial positions and the chloromethyl group in equatorial position. The O–Si–O angle is 176.86(6)° and the Si–CH_2_ bond length was measured as 1.911(2) Å.

### 2.2. Addition of Organolithiums

**Scheme 2:**
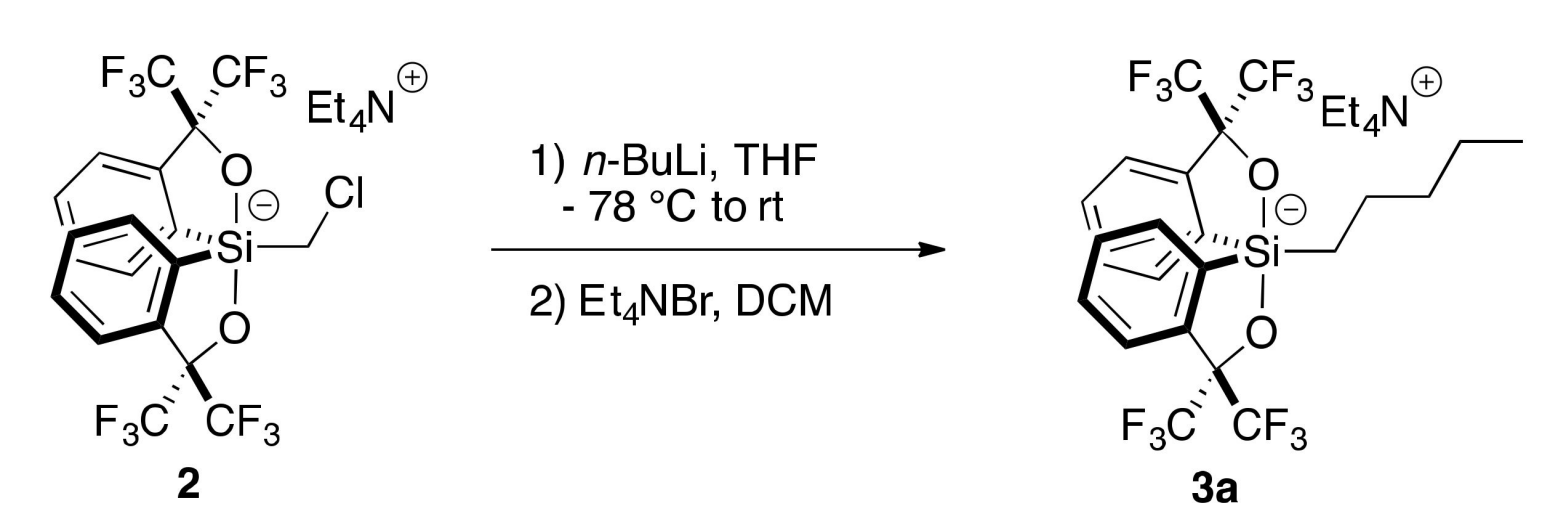
Nucleophilic substitution of chloromethyl silicate **2** with *n*-butyllithium.

With this new compound in hands, we were interested in assessing its reactivity towards strong *C*-nucleophiles. Indeed, if the chloride can be involved in nucleophilic substitution, the chloromethylsilicate **2** would therefore represent a new interesting starting platform for functionalisation. We first tried to make the chloromethylsilicate **2** react with Grignard reagents, but in every case, we could not identify any substitution products. Increasing the strength of the nucleophile had a great effect, since in the presence of *n*-butyllithium, the silicate **2** was totally converted into the product **3a** presenting a *n*-pentyl side chain connected to the silicon atom (Scheme 2).

We then assessed the reactivity of other organolithium compounds. More hindered *tert*-butyllithium, which can also act as a very strong base, proved to readily and selectively react with the methyl silicate **2** to afford the neopentylsilicate **3b** in a good yield of 74% after cation exchange (Table 1, entry 1). Less reactive benzyllithium, easily prepared by deprotonation of toluene by *n*-butyllithium, also added cleanly and led to phenethylsilicate **3c** (entry 2). Surprisingly, the action of methyllitium on silicate **2** provided the expected ethylsilicate **3d** along with methylsilicate **4d**, in an approximately 1:1 ratio (entry 3). This new compound corresponds to the formal addition of methyllithium to spirosilane **1**. The mechanism is not clear as we do not know if either the spirosilane **1** is somehow reformed in the medium or a transmetallation between the C–Si and the C–Li bonds occurs. This intriguing result was also observed, to a lesser extent, when cyclopropyllithium, prepared by metal-halogen exchange from cyclopropylbromide, was used. In that case, expected substitution product **3e** was obtained in 67% yield (entry 4). Phenyllithium could also be used as Csp^2^-nucleophile to afford the benzylsilicate **3f** as the only product of the reaction (entry 5). Less nucleophilic metallated heteroaromatics such as 2-thienyllithium was involved in the same reaction conditions to afford **3g** as the only product (entry 6).

### 2.3. Lewis Acid-Assisted Ring Expansion

**Scheme 3:**
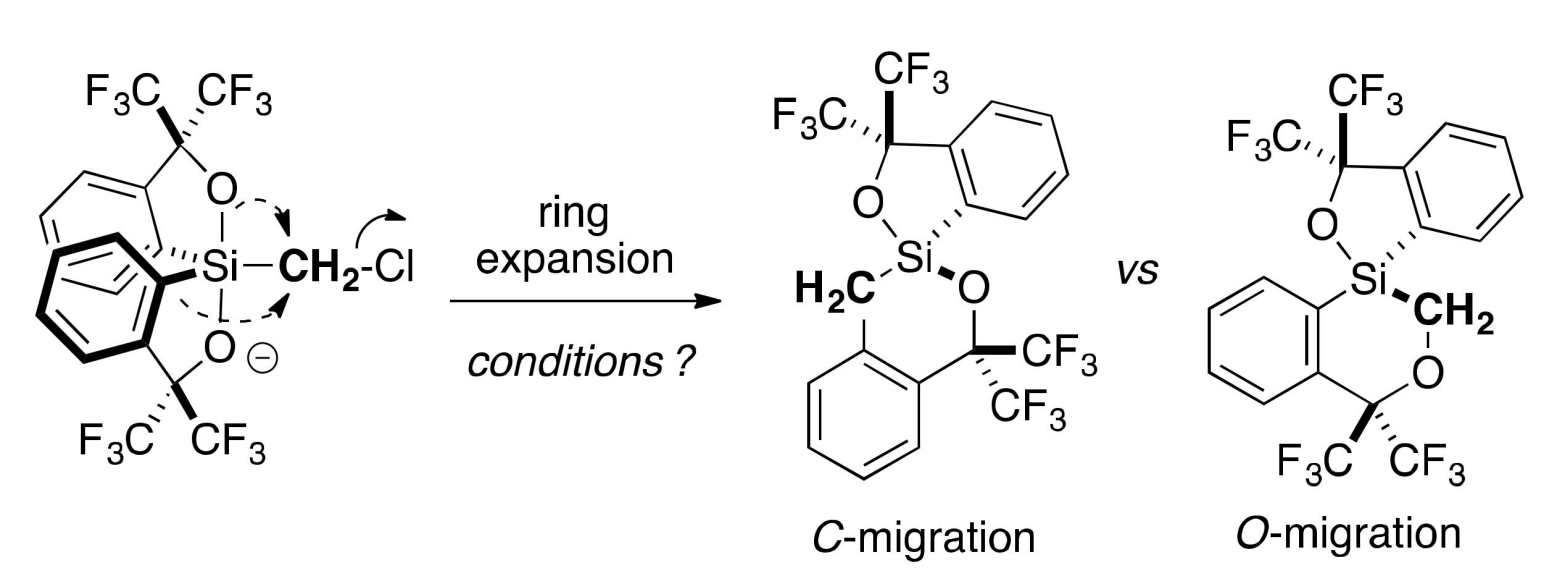
Expected products from ring expansion.

The silicate **2** possesses as particular features a pentacoordinate silicon atom with an extra negative charge dispatched onto the ligands and a methylene bearing a chloride leaving group. One can wonder if such a structure can rearrange via ring expansion, driven by a favorable strain release, to form a new tetravalent species with a six-membered ring (Scheme 3). This behavior, related to a sila-Matteson rearrangement [21,22,23], has already been observed for isolable pentacoordinate [24] and hexacoordinate chloromethylsilicates [25]. *O*-Migration, *N*-migration and *C*-migration have been reported and are usually triggered by thermal conditions when the hypercoordinate species are isolable.

**Figure 3:**
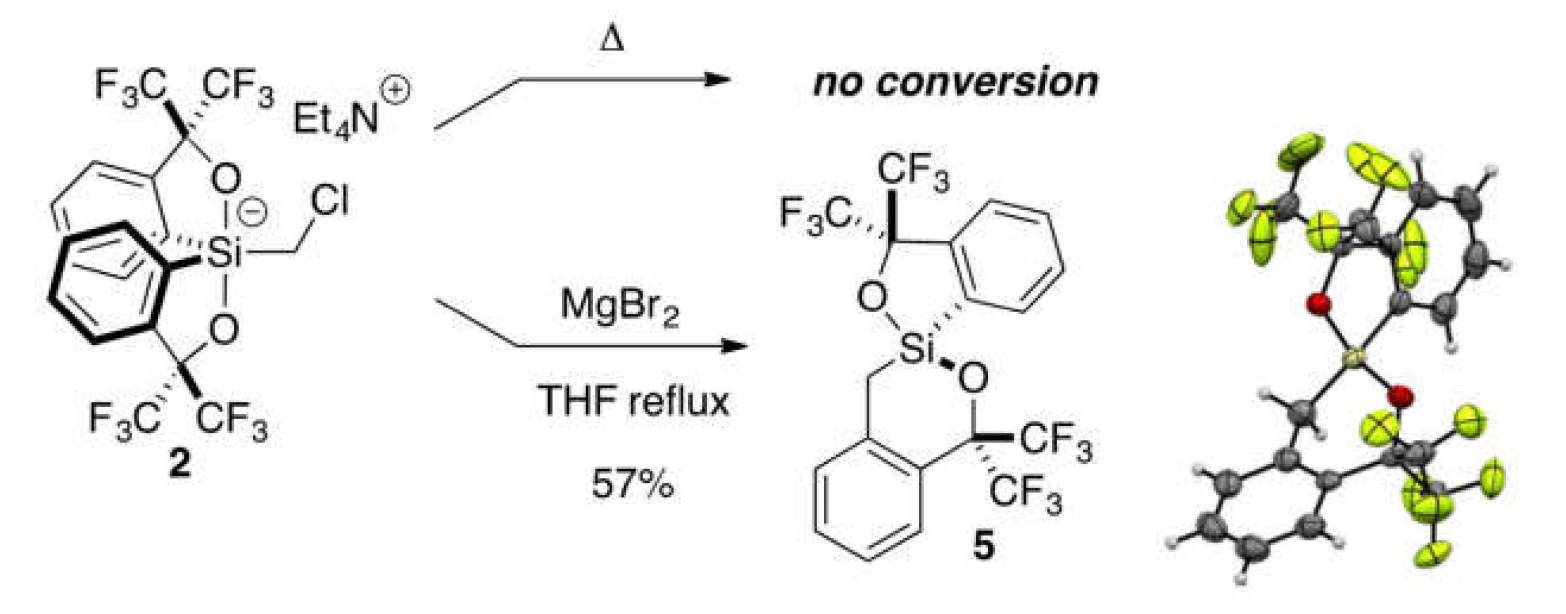
Synthesis of rearranged product **5** and the corresponding X-ray structure (CCDC-2129139) Ellipsoids are shown with 50% of probability.

Heating the chloromethylsilicate **2** at 100 °C in a sealed tube in THF or CH_3_CN did not provide any reaction product and the starting material was always totally recovered. Gratifyingly, the addition of a Lewis acid such as magnesium(II) bromide had a dramatic effect on the reaction course, since the rearrangement was promoted by this additive to provide the new tetravalent spirosilane **5**, in 57% yield, in which a methylene was inserted into the Si–C_Ar_ bond (Figure 3). No *O*-migration product was observed. Crystals of compound **5** could be grown and the structure was confirmed by X-ray diffraction (XRD) analysis.

**Scheme 4:**
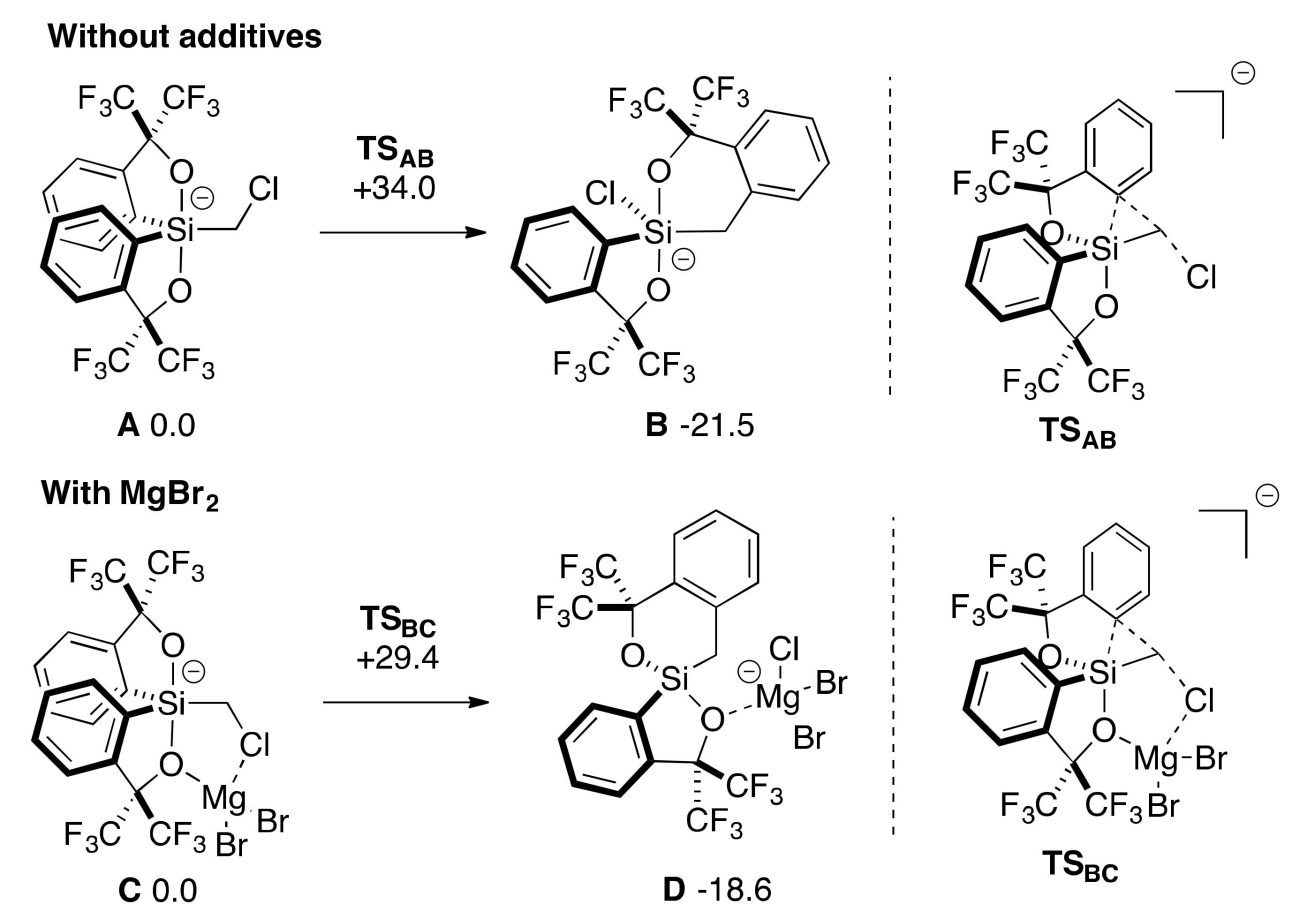
Gibbs free energy profile for the aryl migration with or without magnesium(II) bromide at the B3LYP-D3/6-311+G(2d,p) level of theory. Energies are in kcal·mol^−1^.

In order to get more insight into the mechanism of this ring expansion, we performed some DFT calculations at the B3LYPD3/6311G+(2d,p) level of theory. Although the ring expansion in thermal conditions without any additives could not be observed experimentally, we were able to compute it and determine the associated energies of the whole process. Indeed, a transition state **TS_AB_** corresponding to the aryl migration was found to lie at 34.0 kcal·mol^−1^ above the starting chloromethyl silicate **A** (Scheme 4). In this transition state, the migrating aryl is placed in axial position. Interestingly, the final compound **B** presents a pentacoordinate silicon with the chloride as a ligand. The whole process is exergonic by 21.5 kcal·mol^−1^.

In the presence of a Lewis acid such as magnesium(II) bromide, the same type of mechanism for the migration could be computed with close geometries for the chloromethyl silicate moiety. Interestingly, the Lewis acid interacts with one oxygen of the starting silicate and the chlorine atom. This complexation allows to assist the concerted migration/chloride release step, and therefore, decreases the corresponding energy barrier. In this case, transition state **TS_BC_** lies at 29.4 kcal·mol^−1^ above the starting magnesium(II) silicate complex **C**. The coordination of the chloride to magnesium is also effective in the transition state. Finally, the product is the tetravalent spirosilane **D** with the magnesium(II) chloride not far from the oxygen.

**Figure 4:**
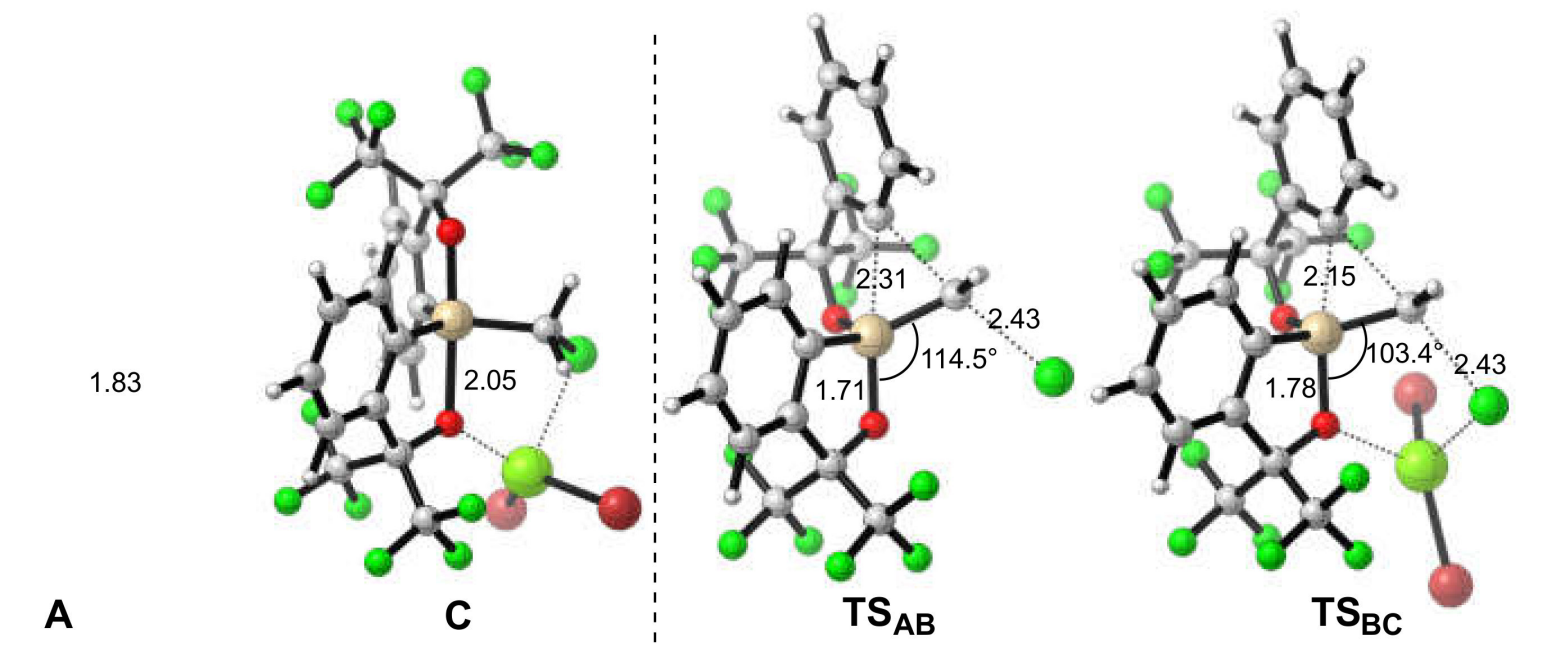
Representation of some relevant calculated structures.

A closer look to the calculated structures allows us to notice some disparities between the two pathways, especially concerning the bond lengths. Indeed, complexation of magnesium(II) to one of the oxygens of chloromethyl silicate significantly increases the Si–O bond length from 1.83 Å to 2.09 Å highlighting a relatively strong interaction (Figure 4). No other substantial changes in bond length can be noticed. Regarding the transition states geometries, without the Lewis acid (**TS_AB_**), the migrating aryl is placed relatively far from the silicon atom with a calculated Si–C_Ar_ bond length of 2.31 Å. In this case, the O–Si–CH_2_ angle is of 114.5° which is much higher than the nearly 90° of the starting trigonal bipyramid. In other words, an energetically costly distortion must take place for the aryl migration to occur. The presence of magnesium leads to some beneficial effect on the transition state (**TS_BC_**). The complexation of magnesium(II) to chloride considerably increases its nucleofugicity and therefore favors the aryl migration. In that case, the Si–C_Ar_ bond is shorter than the one of TS_AB_ by 0.16 Å and the O–Si–CH_2_ is smaller (103.4°). It is noteworthy that in both transition states the C–Cl bond length is the same (2.43 Å).

### 2.4. Lewis Base-Assisted Ring Expansion

**Scheme 5:**
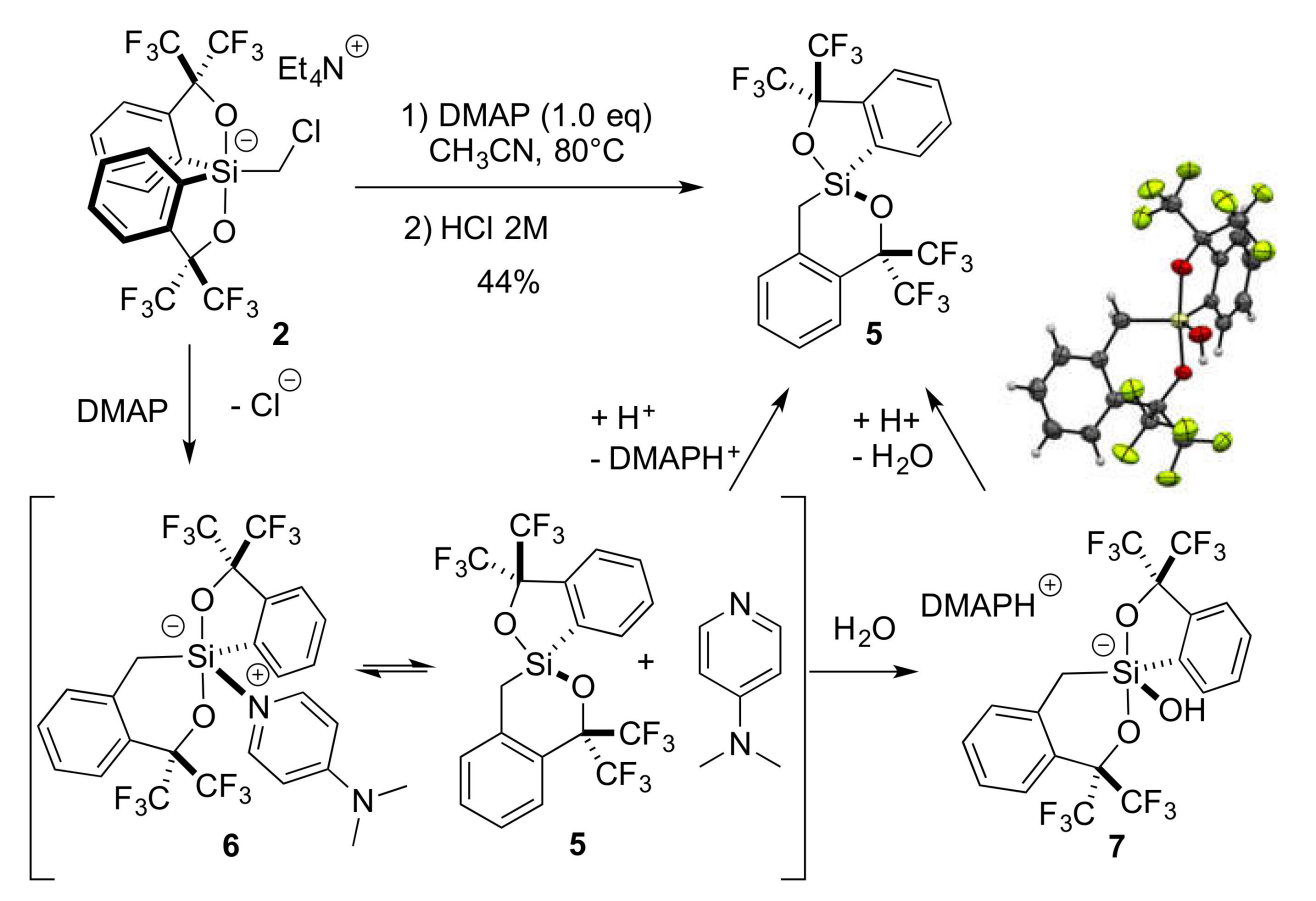
Rearrangement of methylsilicate **2** in Lewis basic conditions. X-ray structure of hydroxy derivative **7** (CCDC-2124350) protonated DMAP was omitted for more clarity. Ellipsoids are shown with 50% of probability.

As previously mentioned, the ring expansion can be triggered by the addition of a Lewis acid. Interestingly, the presence of a Lewis base can also promote the rearrangement. Indeed, when chloromethylsilicate **2** and a stoichiometric amount of *N*,*N*-dimethylaminopyridine (DMAP) was heated in refluxing acetonitrile, the formation of new pentacoordinate silicon derivative **7** with protonated DMAP as counter-ion was obtained after simple aqueous workup (Scheme 5). An X-ray structure was obtained that could confirm unambiguously the rearranged nature of the spirosilane moiety with the methylene insertion into the Si–C_Ar_ bond. Treatment of the hydroxy derivative **8** with a 2 M HCl solution allowed to form the tetravalent compound **5** in 44% as overall yield from **2**. NMR monitoring of the reaction in CD_3_CN was carried out using a J-Young NMR tube which highlights the presence of spirosilane **5** that could be possibly in rapid equilibrium with the pentacoordinate DMAP-adduct **6**. In the presence of water molecules, the hydroxy derivative **7** forms spontaneously.

**Scheme 6:**
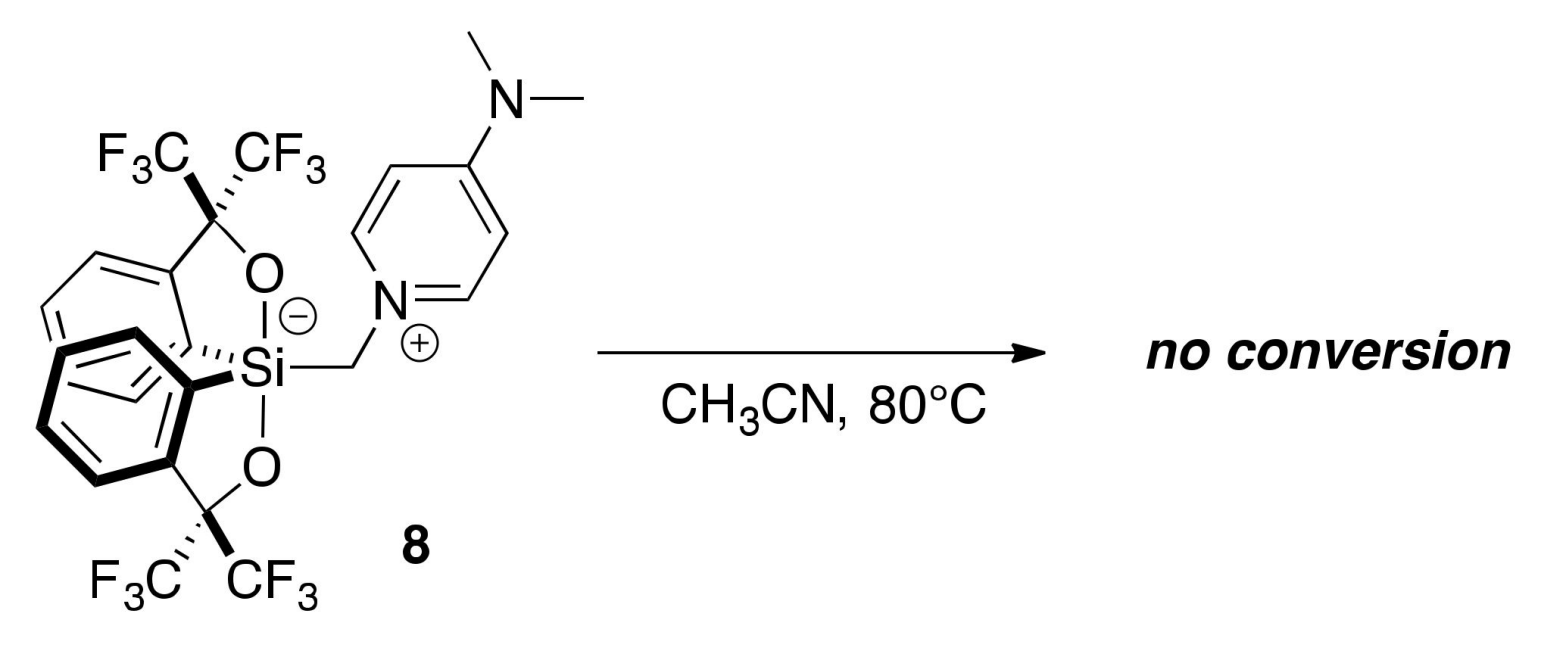
Assessment of the stability of zwitterion **8**.

Interestingly, the NMR monitoring allowed us to observe the presence of a silicate-methylpyridinium by-product **8** (<10%) that originated from the nucleophilic substitution of the chloride by the DMAP. We wondered whether this new compound **8**, which was isolated and fully characterized, was actually a reaction intermediate. It turned out that under the same reaction conditions, zwitterion **8** was very stable and no rearrangement products were detected (Scheme 6) even after prolonged reaction time.

**Scheme 7:**
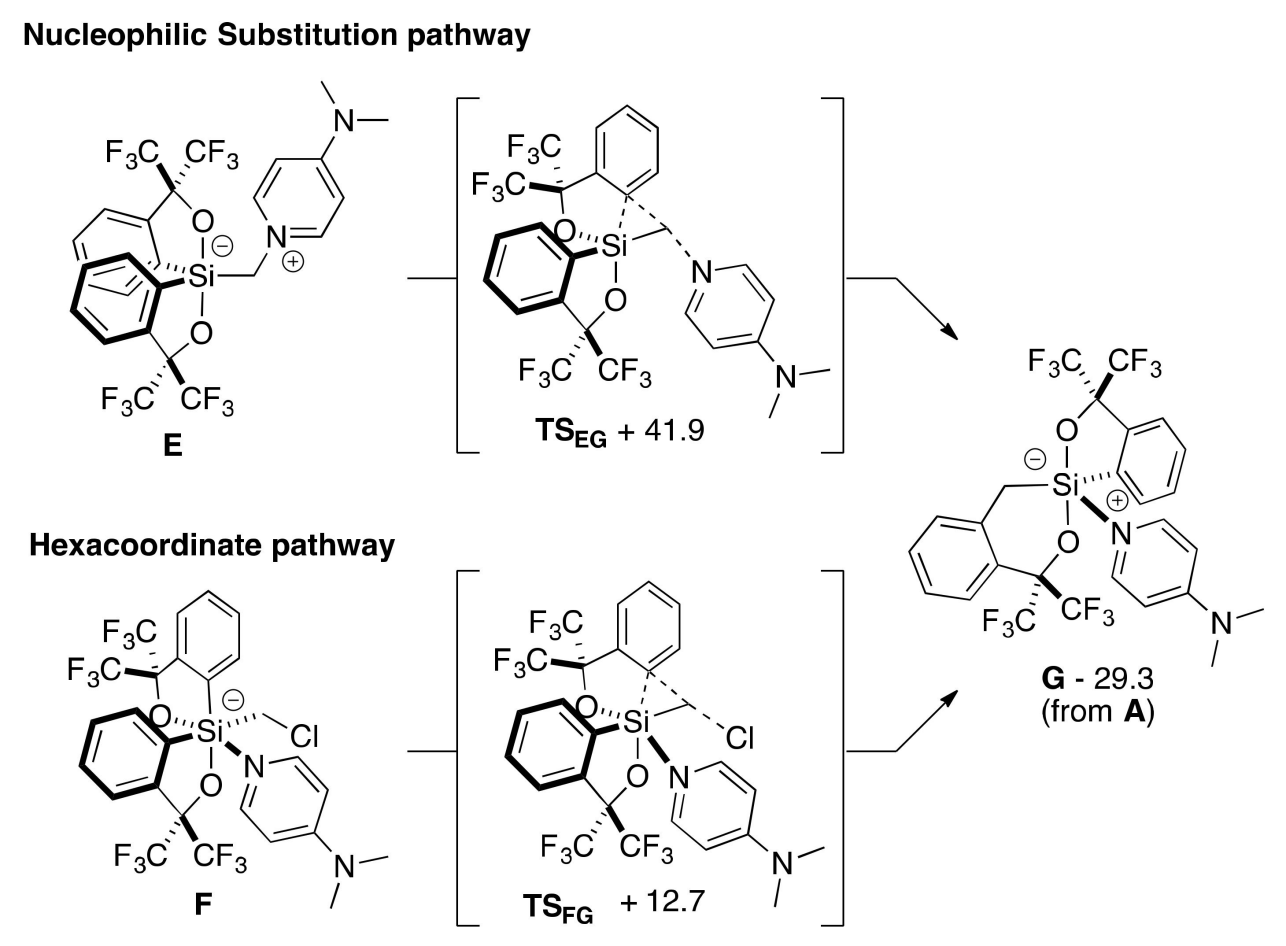
Gibbs free energy profile for the aryl migration with DMAP at the B3LYP-D3/6-311+G(2d,p) level of theory with the CPCM model (acetonitrile). Energies are in kcal·mol^−1^.

These intriguing results prompted us to investigate further on the mechanism of formation of the rearranged product **6** in the presence of DMAP using DFT calculations at the B3LYPD3/6311G+(2d,p) level of theory. The solvent effect was taken into account using the CPCM model (acetonitrile) at the same level of theory. The energetic profile of two rearrangement pathways were evaluated and compared. The first mechanism involves the methylpyridinium intermediate **E** (corresponding to **8** experimentally). A transition state **TS_EG_** for the aryl migration could be calculated for the formation of the final product **G** from **E** where the DMAP goes from the carbon to the silicon atom. It appeared that the aryl migration is very costly in terms of energy with **TS_EG_** lying 41.9 kcal·mol^−1^ above intermediate **E** (Scheme 7).

**Scheme 8:**
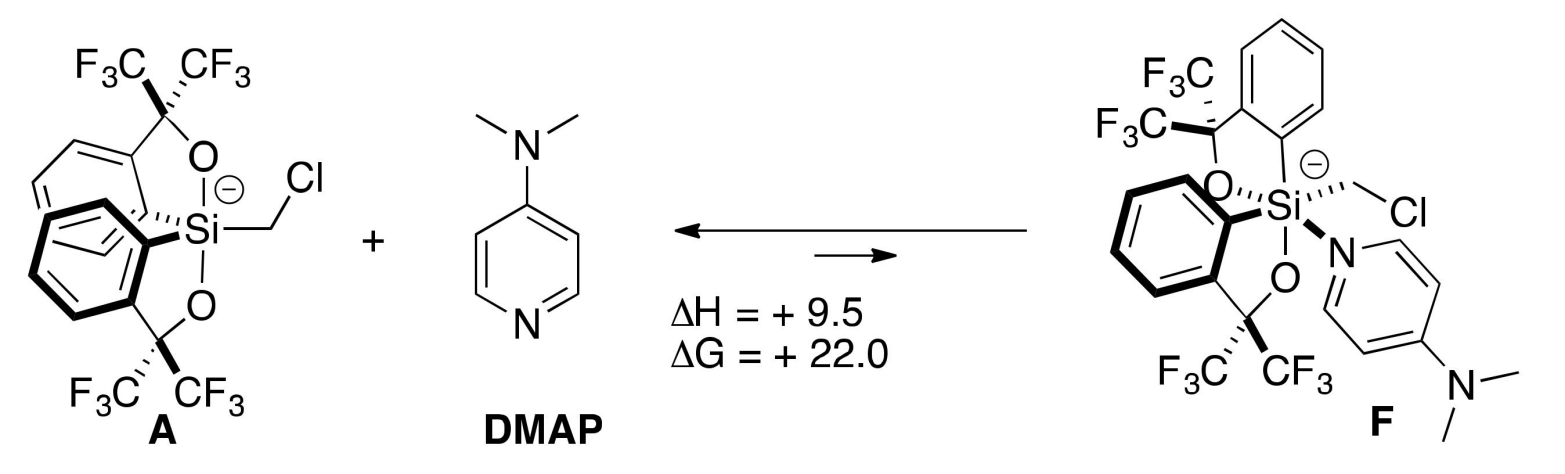
Energies associated to the formation of hexavalent adduct **F** at the B3LYP-D3/6-311+G(2d,p) level of theory with the CPCM model (acetonitrile). Energies are in kcal·mol^−1^.

The second envisaged mechanism involves the formation of a transient hexacoordinate intermediate **F** that would be formed by addition of DMAP onto chloromethylsilicate **2**. Interestingly, **F** is the only stable hexacoordinate isomer we were able to compute and its structure is similar to the only example of Martin ligand-based hexacoordinate silicon derivative described in the literature with phenantroline [26]. A transition state **TS_FG_** could be found for the aryl migration leading to the product **7**. In terms of energies, the aryl migration from **F** to **G** is quite low, about 12.7 kcal·mol^−1^, compared to all other migration barriers calculated so far. The migration step seems to be totally feasible from the hexacoordinate intermediate **F**. The question arising is the ability to form **F** in the medium. The adduct formation with DMAP is not very high in terms of enthalpy (only 9.5 kcal·mol^−1^) (Scheme 8). However, the Gibbs free energy is much higher (22.0 kcal·mol^−1^) because of the entropically disfavored nature of this bimolecular reaction. This mechanism is realistic even though we do not yet have any experimental evidence for the formation of a hexacoordinate intermediate.

## 3. Experimental

### 3.1. General Information

Unless otherwise noted, reactions were carried out under an argon atmosphere in oven-dried glassware using Schlenk line techniques. All the solvents were distilled prior to be used. Dichloromethane was distilled over CaH_2_, THF and Et_2_O were distilled over sodium/benzophenone. Unless otherwise noted, reagents and chemicals were purchased from commercial sources and used as received. ESI-MS experiments were carried out using a LTQ-Orbitrap XL from Thermo Scientific and operated in negative or positive ionization mode. ^1^H, ^19^F, ^13^C, ^29^Si NMR spectra were recorded at room temperature at 400, 376,101 and 79 MHz, respectively, on a Brucker AVANCE 400 spectrometer. ^1^H, ^19^F, ^13^C, ^29^Si NMR spectra were recorded at room temperature at 300, 282, 75 and 60 MHz on a Brucker AVANCE 300 nanobay spectrometer. Chemical shifts (δ) are reported in ppm and coupling constants (*J*) are given in Hertz (Hz). Abbreviations used for peak multiplicity are: s (singlet); d (doublet); t (triplet); q (quartet); hept. (heptuplet); m (multiplet); td (triplet of doublets).

### 3.2. Synthesis and Characterization

Synthesis and characterization of Martin’s spirosilane **1** was reported previously [15].

#### 3.2.1. Synthesis of Chloromethylsilicate **2**

In a dry Schlenk flask, Martin’s spirosilane **1** (1 g, 1.95 mmol, 1.0 eq.) was dissolved in THF (0.05 M). Distilled bromochloromethane (0.25 mL, 3.90 mmol; 2.0 eq.) was added and the mixture was cooled down to −80 °C. *n*-BuLi 2.5 M in hexanes (1.6 mL; 3.90 mmol, 2.0 eq.) was added dropwise along the flask’s wall over 1h by means of a syringe pump. By the end of the addition, the mixture was slowly warmed up to room temperature and stirred overnight. The brown solution was quenched with EtOH abs., and then solvents were removed under reduced pressure. The resulting residue was dissolved in DCM (0.1 M), then tetraethylammonium bromide (1.6 g, 7.8 mmol, 4.0 eq.) was added, and the mixture was stirred for 1h at room temperature. Water was added and the organic layer was washed with water (×3), dried over Na_2_SO_4_, filtered, and finally concentrated in vacuo. The crude product was dissolved in minimal volume of DCM and precipitation was triggered by addition of pentane. Filtration afforded chloromethylsilicate **2** as an off-white solid (890 mg, 66%). ^1^H NMR: (300 MHz, Acetone-*d*_6_) δ 8.30–8.21 (m, 2H, C*H_Ar_*), 7.52 (d, *J* = 6.6 Hz, 2H, C*H_Ar_*), 7.41–7.28 (m, 4H, C*H_Ar_*), 3.52 (q, *J* = 7.3 Hz, 8H, NC*H*_2_CH_3_), 2.91 (s, 2H, SiC*H*_2_Cl), δ 1.59–1.33 (m, 12H, NCH_2_C*H*_3_). ^13^C NMR: (75 MHz, Acetone-*d*_6_) δ 143.1, 141.3, 137.8, 126.5, 123.7 (q, ^1^*J*_C_–_F_ = 287.7 Hz, 2 × *C*F_3_), 121.9 (q, ^1^*J*_C_–_F_ = 287.7 Hz, 2 × *C*F_3_), 81.2 (hept., ^2^*J*_C_–_F_ = 28.5 Hz, 2 × *C*(CF_3_)_2_), 54.3–48.9 (m), 37.2, 6.7. ^19^F NMR: (282 MHz, Acetone-*d*_6_) δ −75.21 (q, *J* = 9.5 Hz), −75.78 (q, *J* = 9.5 Hz). ^1^H/^29^Si HMQC: (300 MHz/60 MHz, Acetone-*d*_6_) δ 8.27/−70.7, δ 2.91/−70.7. HRMS (ESI^−^): *m/z* calcd for C_19_H_10_ClF_12_O_2_Si^−^: 560.9952, found 560.9939. Anal. Calcd for C_27_H_30_ClF_12_NO_2_Si: C, 46.86; H, 4.37; N, 2.02. Found: C, 46.78; H, 3.72; N, 1.88. Melting point: 130–131 °C.

#### 3.2.2. Addition of Organolithium

General procedure: In a dry Schlenk flask, chloromethylsilicate **2** (1.0 eq.) was dissolved in THF or Et_2_O (0.1 M). The mixture was cooled to −78 °C and the organolithium reagent (3.0 eq.) was added dropwise. The reaction was warmed up to room temperature and stirred overnight. Quenching was achieved by addition of EtOH abs., and solvents were removed in vacuo. The resulting residue was diluted in DCM (0.1 M), then tetraethylammonium bromide (4.0 eq.) was added, and the resulting mixture was stirred at room temperature for 1h. Water was added, and the organic layer was washed with water (×3), dried over Na_2_SO_4_, filtered and concentrated under reduced pressure. The crude product was dissolved in minimal volume of DCM and precipitation was triggered by addition of pentane. Filtration afforded silicate **3**.

*tert-Butylsilicate***3b**. Synthesized according to the general procedure starting from chloromethylsilicate **2** (77 mg, 0.11 mmol) in Et_2_O and *tert*-BuLi (1.7 M in pentane, 0.19 mL, 0.33 mmol). Precipitation afforded *tert*-butylsilicate **3b** as a white solid (58 mg, 74%). ^1^H NMR: (400 MHz, Acetone-*d*_6_) δ 8.31 (d, *J* = 7.3 Hz, 2H, C*H_Ar_*), 7.45 (d, *J* = 8.0 Hz, 2H, C*H_Ar_*), 7.34–7.20 (m, 4H, C*H_Ar_*), 3.51 (q, *J* = 7.3, 8H, NC*H*_2_CH_3_), 1.41 (t, *J* = 7.4 Hz, 12H, NCH_2_C*H*_3_), 1.22 (td, *J* = 7.1, 1.8 Hz, 1H, SiC*H*_2_C(CH_3_)_3_), 1.13–1.05 (m, 1H, SiC*H*_2_C(CH_3_)_3_), 0.87 (d, *J* = 1.9 Hz, 9H, SiCH_2_C(C*H*_3_)_3_).^13^C NMR: (101 MHz, Acetone-*d*_6_) δ 147.09, 140.93, 137.77, 127.57, 127.27, 126.15, 123.72 (q, ^1^*J*_C_–_F_ = 287.7 Hz, 2 × *C*F_3_), 122.93 (q, ^1^*J*_C_–_F_ = 287.7 Hz, 2 × *C*F_3_), 81.2 (hept., ^2^*J*_C_–_F_ = 28.5 Hz, 2 × *C*(CF_3_)_2_), 53.76–47.60 (m), 39.40, 33.17, 31.10, 6.72. ^19^F NMR: (282 MHz, Acetone-*d*_6_) δ −74.10 (q, *J* = 9.8, 1.6 Hz), −75.39 (q, *J* = 9.8, 1.6 Hz). ^1^H/^29^Si HMQC: (400 MHz/79 MHz, Acetone-*d*_6_) δ 1.11/−64.5, δ 0.86/−64.5. HRMS (ESI^−^): *m/z* calcd for C_23_H_19_F_12_O_2_Si^−^: 583.0957, found 583.0969. Anal. Calcd for C_31_H_39_F_12_NO_2_Si: C, 52.17; H, 5.51; N, 1.96. Found: C, 52.03; H, 4.54; N, 1.88. Melting point: 151–152 °C.

*Phenylethylsilicate***3c**. Synthesized according to the general procedure starting from chloromethylsilicate **2** (70 mg, 0.10 mmol) in THF and PhCH_2_Li (see Supplementary Materials for its preparation, 0.30 mmol). Precipitation afforded phenylethylsilicate 3c as a white solid (54 mg, 72%).^1^H NMR: (400 MHz, Acetone-*d*_6_) δ 8.34–8.27 (m, 2H, C*H_Ar_*), 7.55–7.48 (m, 2H, C*H_Ar_*), 7.38–7.25 (m, 4H, C*H_Ar_*), 7.16–6.94 (m, 5H, C*H_Ar_*), 3.50 (q, *J* = 7.3 Hz, 8H, NC*H*_2_CH_3_), 3.07–2.94 (m, 1H, SiCH_2_C*H*_2_Ph), 2.33 (td, *J* = 13.5, 5.6 Hz, 1H, SiCH_2_C*H*_2_Ph), 1.45–1.36 (t, *J* = 7.6 Hz, 12H, NCH_2_C*H*_3_), 1.18–1.00 (m, 2H, SiC*H*_2_CH_2_Ph). ^13^C NMR: (101 MHz, Acetone-*d*_6_) δ 148.7, 145.2, 141.2, 137.6, δ 127.8, 127.7, 127.7, 126.2 (q, ^1^*J*_C_–_F_ = 287.7 Hz, 2 × *C*F_3_), 124.2 (q, ^1^*J*_C_–_F_ = 287.7 Hz, 2 × *C*F_3_), 123.2, 81.9 (hept., ^2^*J*_C_–_F_ = 28.5 Hz, 2 × *C*(CF_3_)_2_), 53.7–49.2 (m), 31.9, 26.5, 6.7. ^19^F NMR: (282 MHz, Acetone) δ −75.22 (q, *J* = 9.5 Hz), −75.67 (q, *J* = 9.3 Hz). ^1^H/^29^Si HMQC: (400 MHz/79 MHz, Acetone-*d*_6_) δ 8.29/−65.3, δ 1.07/−65.3. HRMS (ESI^−^): *m/z* calcd for C_26_H_17_F_12_O_2_Si^−^: 617.0812, found 617.0789. Melting point: 166–167 °C.

*Ethylsilicate***3d***and methylsilcate***4d**. Synthesized according to the general procedure starting from chloromethysilicate **2** (70 mg, 0.10 mmol) in THF and MeLi (1.6 M in Et_2_O, 0.19 mL, 0.30 mmol). Precipitation afforded a mixture of targeted ethylsilicate **3d** and methylsilicate **4d** (55:45) as a white solid (25 mg, 36% overall). **3d**: ^1^H NMR: (300 MHz, Acetone-*d*_6_) δ 8.27 (d, *J* = 7.1 Hz, 2H, C*H_Ar_*), 7.49 (s, 2H, C*H_Ar_*), 7.30 (s, 4H, C*H_Ar_*), 3.51 (q, *J* = 7.4 Hz, 8H, NC*H*_2_CH_3_), 1.41 (t, *J* = 7.6 Hz, 12H, NCH_2_C*H*_3_), 0.89 (t, *J* = 7.9 Hz, 3H, SiCH_2_C*H*_3_), 0.76 (d, *J* = 8.4 Hz, 2H, SiC*H*_2_CH_3_). ^13^C NMR: (101 MHz, Acetone-*d*_6_) δ 145.8, 141.2, δ 137.6, 137.3, 127.7 (q, ^1^*J*_C_–_F_ = 287.7 Hz, 2 × *C*F_3_), 123.1 (q, ^1^*J*_C_–_F_ = 287.7 Hz, 2 × *C*F_3_), 77.9 (hept., ^2^*J*_C_–_F_ = 28.5 Hz, 2 × *C*(CF_3_)_2_), 56.0–48.6 (m), 6.7. ^19^F NMR: (282 MHz, Acetone-*d*_6_) δ −75.34 (q, *J* = 10.4 Hz), −75.61 (q, *J* = 9.7 Hz). ^1^H/^29^Si HMQC: (400 MHz/79 MHz, Acetone-*d*_6_) δ 0.89/−63.4. HRMS (ESI^−^): *m/z* calcd for C_20_H_13_F_12_O_2_Si^−^: 541.0499, found 541.0498. **4d**: ^1^H NMR: (300 MHz, Acetone-*d*_6_) δ 8.27 (d, *J* = 7.1 Hz, 2H), 7.49 (s, 2H), 7.30 (s, 4H), 3.51 (q, *J* = 7.4 Hz, 8H), 1.41 (t, *J* = 7.6 Hz, 12H), 0.89 (t, *J* = 7.9 Hz, 3H), 0.25 (s, 3H). ^19^F NMR: (282 MHz, Acetone-*d*_6_) δ −75.38 (q, *J* = 9.3 Hz), −75.61 (q, *J* = 9.1 Hz). ^1^H/^29^Si HMQC: (400 MHz/79 MHz, Acetone-*d*_6_) δ 0.27/−64.6. NMR analyses were consistent with data reported in the literature [19].

*Cyclopropylmethylsilicate***3e** *and cyclopropylsilicate***4e**. Synthesized according to the general procedure starting from chloromethylsilicate **2** (70 mg, 0.10 mmol, 1.0 eq.) in Et_2_O and cyclopropyllithium (see Supplementary Materials for its preparation, 0.3 mmol). Precipitation afforded a mixture of targeted cyclopropylmethylsilicate **3e** and cyclopropylsilicate **4e** (83:17) as a white solid (56 mg, 81% overall). **3e**: ^1^H NMR: (300 MHz, Acetone-*d*_6_) δ 8.30 (d, *J* = 7.2 Hz, 2H, C*H_Ar_*), 7.47 (d, *J* = 7.2 Hz, 2H, C*H_Ar_*), 7.32–7.23 (m, 4H, C*H_Ar_*), 3.50 (q, *J* = 7.3 Hz, 8H, NC*H*_2_CH_3_), 1.41 (t, *J* = 7.6 Hz, 12H, NCH_2_C*H*_3_), 0.91–0.79 (m, 2H, SiC*H*_2_C_3_H_5_), 0.68–0.59 (m, 1H, SiCH_2_C*H*CH_2_CH_2_), 0.18–0.06 (m, 1H, SiCH_2_CHC*H*_2_C*H*_2_), 0.05–−0.01 (m, 1H, SiCH_2_C_3_*H*_5_), −0.03–−0.09 (m, 1H, SiCH_2_C_3_*H*_5_), −0.41–−0.44 (m, 1H, SiCH_2_C_3_*H*_5_). ^13^C NMR: (101 MHz, Acetone-*d*_6_) δ 145.94, 141.04, 137.65, 127.61, 127.49, 126.23 (q, ^1^*J*_C_–_F_ = 287.7 Hz, 2 × *C*F_3_), 122.98 (q, ^1^*J*_C_–_F_ = 287.7 Hz, 2 × *C*F_3_), 81.3 (hept., ^2^*J*_C_–_F_ = 28.5 Hz, 2 × *C*(CF_3_)_2_) 54.45–46.99 (m), 10.38–1.09 (m). ^19^F NMR: (376 MHz, Acetone-*d*_6_) δ −75.05 (q, *J* = 9.6 Hz), −75.63 (q, *J* = 9.5 Hz). ^1^H/^29^Si HMQC: (300 MHz/60 MHz, Acetone-*d*_6_) 0.64/−64.5 δ. HRMS (ESI^−^): *m/z* calcd for C_22_H_15_F_12_O_2_Si^−^: 567.0655, found 567.0648. **4e**: ^1^H NMR: (300 MHz, Acetone-*d*_6_) δ 8.24 (d, *J* = 7.3 Hz, 2H), 7.47 (d, *J* = 7.2 Hz, 2H), 7.32–7.23 (m, 4H), 3.50 (q, *J* = 7.3 Hz, 8H), 1.41 (t, *J* = 7.6 Hz, 12H), 0.47–0.38 (m, 1H), 0.33–0.26 (m, 1H), 0.18–0.06 (m, 1H), −0.19–−0.26 (m, 1H). ^13^C NMR (101 MHz, Acetone) δ 145.67, 141.05, 137.46, 127.68, 127.55, 126.29 (q, ^1^*J*_C-F_ = 287.7 Hz, 2 × *C*F_3_), 123.42 (q, ^1^*J*_C-F_ = 287.7 Hz, 2 × *C*F_3_), 123.02–122.82 (m), 81.97 (hept., ^2^*J*_C-F_ = 28.5 Hz, 2 × *C*(CF_3_)_2_), 52.25–52.03 (m), 6.70, 2.11, 1.69, 1.1. ^19^F NMR: (376 MHz, Acetone-*d*_6_) δ −74.50 (q, *J* = 7.5 Hz), −75.36 (q, *J* = 7.6 Hz).

*Benzylsilicate***3f**. Synthesized according to the general procedure starting from chloromethylsilicate **2** (70 mg, 0.10 mmol) in THF and PhLi (1.9 M in dibutyl ether, 0.16 mL, 0.30 mmol). Precipitation afforded benzylsilicate **3f** as a white solid (45 mg, 61%). ^1^H NMR: (400 MHz, Acetone-*d*_6_) δ 8.15–8.11 (m, 2H, C*H_Ar_*), 7.51–7.43 (m, 2H, C*H_Ar_*), 7.27–7.23 (m, 4H, C*H_Ar_*), 7.05–6.98 (m, 2H, C*H_Ar_*), 6.88 (m, 2H, C*H_Ar_*), 6.80–6.70 (m, 1H, C*H_Ar_*), 3.49 (q, *J* = 7.3 Hz, 8H, NC*H*_2_CH_3_), 2.42–2.29 (m, 2H, SiC*H*_2_Ph), 1.41 (t, *J* = 7.6 Hz, 12H, NCH_2_C*H*_3_).^13^C NMR: (101 MHz, Acetone-*d*_6_) δ 144.9, 144.3, 141.0, 137.8, 129.4, 127.7, 127.6, 126.1, 123.6 (q, ^1^*J*_C_–_F_ = 287.7 Hz, 2 × *C*F_3_), 123.2, 123.0 (q, ^1^*J*_C_–_F_ = 287.7 Hz, 2 × *C*F_3_), 121.9, 81.6 (hept., ^2^*J*_C_–_F_ = 28.5 Hz, 2 × *C*(CF_3_)_2_), 52.9–51.8 (m), 32.9, 6.7. ^19^F NMR: (376 MHz, Acetone-*d*_6_) δ −74.66 (q, *J* = 9.5 Hz), −75.56 (q, *J* = 9.6 Hz). ^1^H/^29^Si HMQC: (400 MHz/79 MHz, Acetone-*d*_6_) δ 8.15/−66.3, δ 2.34/−66.3. HRMS (ESI^−^): *m/z* calcd for C_25_H_15_F_12_O_2_Si^−^: 603.0655, found 603.0651. Melting point: 145–146 °C.

*Thienylmethylsilicate***3g**. Synthesized according to the general procedure starting from chloromethylsilicate **2** (70 mg, 0.10 mmol) in THF and 2-thienyllithium (see Supplementary Materials for its preparation, 0.30 mmol). Precipitation afforded phenylethylsilicate **3g** as a white solid (50 mg, 69%). ^1^H NMR: (400 MHz, Acetone-*d*_6_) δ 8.28–8.17 (m, 2H, C*H_Ar_*), 7.50 (s, 2H, C*H_Ar_*), 7.38–7.24 (m, 4H, C*H_Ar_*), 6.73 (d, *J* = 5.2 Hz, 1H, C_4_*H*_4_S), 6.60 (dd, *J* = 5.2, 3.4 Hz, 1H SiC*H*_2_(C_4_*H*_4_S)), 6.53 (d, *J* = 3.4 Hz, 1H, SiC*H*_2_(C_4_*H*_4_S)), 3.47 (q, *J* = 7.3 Hz, 8H, NC*H*_2_CH_3_), 2.58–2.44 (m, 2H, SiC*H*_2_(C_4_H_4_S)), 1.38 (t, *J* = 7.6 Hz, 12H, NCH_2_C*H*_3_). ^13^C NMR: (101 MHz, Acetone-*d*_6_) δ 146.31, 144.46, 141.11, 137.88, 128.73, 128.04, 127.90, 127.75, 126.35, 126.10, 125.41, 123.49, 123.23 (q, ^1^*J*_C_–_F_ = 287.7 Hz, 2 × *C*F_3_), 123.09 (q, ^1^*J*_C_–_F_ = 287.7 Hz, 2 × *C*F_3_), 122.93, 119.48, 81.45 (hept., ^2^*J*_C_–_F_ = 28.5 Hz, 2 × *C*(CF_3_)_2_), 52.14, 52.11, 52.08, 26.38, 6.73. ^19^F NMR: (376 MHz, Acetone) δ −74.88 (q, *J* = 9.4 Hz), −75.65 (q, *J* = 9.5 Hz). ^1^H/^29^Si HMQC: (400 MHz/79 MHz, Acetone-*d*_6_) δ 8.21/−68.1, δ 2.49/−68.1. HRMS (ESI^−^): *m/z* calcd for C_23_H_13_F_12_O_2_SSi^−^: 609.0219, found 609.0192. Melting point: 150 °C.

#### 3.2.3. Synthesis of Spirosilane **5**

*Method A with a Lewis acid*: In a dry Schlenk flask chloromethylsilicate **2** (160 mg, 0.23 mmol, 1.0 eq.) and MgBr_2_ (60 mg, 0.23 mmol, 1.0 eq.) were dissolved in THF (0.1 M). The resulting mixture was refluxed overnight while stirred. Water was added and extraction with DCM was performed. Organic layers were combined, washed with water, dried over Na_2_SO_4_, filtered and concentrated under reduced pressure. The resulting residue was diluted in Et_2_O and the solid was filtered off. The filtrate was concentrated in vacuo and recrystallized in DCM to afford colorless crystals of silane **5** (86 mg, 57%). ^1^H NMR: (400 MHz, CDCl_3_) δ 7.81–7.59 (m, 5H, C*H_Ar_*), 7.49–7.45 (m, 1H, C*H_Ar_*), 7.41–7.34 (m, 2H, C*H_Ar_*), 2.68 (A of AB, *J* = 17.4 Hz, 1H, SiC*H*_2_C_Ar_), 2.59 (B of AB, *J* = 17.4 Hz, 1H, SiC*H*_2_C_Ar_). ^13^C NMR: (75 MHz, CDCl_3_) δ 140.9, 133.9, 133.6, 132.6, 132.4, 131.1, 130.7, 129.2, 127.9, 126.9, 126.8, 125.3, 124.5 (q, ^1^*J*_C_–_F_ = 287.7 Hz, 2 × *C*F_3_), 120.7 (q, ^1^*J*_C_–_F_ = 287.7 Hz, 2 × *C*F_3_), 81.1 (hept., ^2^*J*_C_–_F_ = 28.5 Hz, 2 × *C*(CF_3_)_2_), 53.4, 6.9. ^19^F NMR: (376 MHz, CDCl_3_) δ −73.97 (q, *J* = 8.9 Hz), −75.42 (q, *J* = 9.0 Hz), −76.20. ^1^H/^29^Si HMQC: (400 MHz/79 MHz, CDCl_3_) δ 2.63/3.1. HRMS (ESI^+^): *m/z* calcd for C_19_H_10_F_12_O_2_Si [M + H]: 527.0297, found 527.0333. Anal. Calcd for C_19_H_10_F_12_O_2_Si: C, 43.36; H, 1.92. Found: C, 43.23; H, 2.51. Melting point: 118–119 °C.

*Method B with a Lewis base:* In a dry Schlenk flask chloromethylsilicate **2** (70 mg, 0.10 mmol, 1.0 eq.) and DMAP (12 mg, 0.10 mmol, 1.0 eq.) were dissolved in acetonitrile (0.1 M). The resulting mixture was refluxed overnight. HCl (0.5 M) was added, and extraction with DCM was carried out. Organic layers were combined, washed with water, dried over Na_2_SO_4_, filtered and concentrated under reduced pressure. The resulting residue was diluted in Et_2_O and the solid was filtered off. The filtrate was concentrated in vacuo and recrystallized in DCM to afford colorless crystals of silane **5** (23 mg, 44%).

#### 3.2.4. Obtaining Crystals of Hydroxysilicate **7**

According to the method B in a J Young NMR tube from chloromethylsilicate **2** (20 mg, 0.029 mmol) and DMAP (3.5 mg, 0.029 mmol) in acetonitrile-*d*_3_ (0.4 mL). Crystals of hydroxysilicate **7** were obtained after slow evaporation in non-inert conditions. ^1^H NMR: (400 MHz, Acetone-*d*_6_) δ 8.34–8.28 (m, 1H, C*H_Ar_*), 7.62–7.53 (m, 3H, C*H_Ar_*), 7.48–7.41 (m, 3H, C*H_Ar_*), 7.28–7.24 (m, 1H, C*H_Ar_*), 7.19–7.14 (m, 1H, C*H_Ar_*) 6.80 (d, *J* = 6.6 Hz, 2H, C*H_Ar_*), 3.16 (s, 6H, N(C*H*_3_)_2_), 2.31 (A of AB, *J* = 15.4 Hz, 1H, SiC*H*_2_C_Ar_), 2.14 (B of AB, *J* = 15.1 Hz, 1H, SiC*H*_2_C_Ar_)**.**^13^C NMR (75 MHz, CD_3_CN) δ 156.8, 143.6, 142.9, 141.3, 141.2, 137.4, 132.3, 128.92 (d, *J* = 2.8 Hz), 128.7, 128.2, 126.7, 124.1, 123.6, 106.7, 39.2, 25.9. ^19^F NMR (282 MHz, CD_3_CN) δ −72.29, −76.39 (d, *J* = 58.6 Hz), −77.17. ^1^H/^29^Si HMQC: (400 MHz/79 MHz, CD_3_CN) δ 8.29/−86.1.

HRMS (ESI^−^): *m/z* calcd for C_19_H_11_F_12_O_3_Si^−^: 543.0288, found 543.0291.

Hydroxysilicate **7** was converted to hydrosilicate **7**-Et_4_N^+^ after treatment with Et_4_NBr (4.0 eq.) in DCM (0.1 M) at room temperature.

^1^H NMR: (400 MHz, Acetone-*d*_6_) δ 8.47–8.36 (m, 1H, C*H_Ar_*), 7.56–7.48 (m, 3H, C*H_Ar_*), 7.43–7.28 (m, 2H, C*H_Ar_*), 7.25–7.05 (m, 3H, C*H_Ar_*), 3.48 (q, *J* = 7.3 Hz, 8H, NC*H*_2_CH_3_), 2.32 (A of AB, *J* = 15.0 Hz, 1H, SiC*H*_2_C_Ar_), 2.18 (B of AB, *J* = 15.0 Hz, 1H, SiC*H*_2_C_Ar_), 1.38 (t, *J* = 7.6 Hz, 12H, NCH_2_C*H*_3_). ^13^C NMR: (101 MHz, Acetone-*d*_6_) δ 144.6, 141.5, 137.7, 132.3, 128.7, 128.1, 127.9, 126.5 (q, ^1^*J*_C_–_F_ = 287.7 Hz, 2 × *C*F_3_), 123.3 (q, ^1^*J*_C_–_F_ = 287.7 Hz, 2 × *C*F_3_), 83.1 (hept., ^2^*J*_C_–_F_ = 28.5 Hz, 2 × *C*(CF_3_)_2_), 52.1, 26.4, 6.7. ^19^F NMR: (376 MHz, Acetone) δ −71.81 (t, *J* = 9.7 Hz), −76.16 (q, *J* = 9.2 Hz), −76.35 (q, *J* = 9.1 Hz), −77.35 (q, *J* = 9.7 Hz). ^1^H/^29^Si HMQC: (400 MHz/79 MHz, Acetone-*d*_6_) 2.21/−86.9. HRMS (ESI^−^): *m/z* calcd for C_19_H_11_F_12_O_3_Si [M] 543.0288, found 543.0291. Anal. Calcd for C_19_H_10_F_12_O_2_Si: C, 48.14; H, 4.64; N,2.08. Found: C, 47.95; H, 4.25; N, 1.95.

## 4. Conclusions

The formation of chloromethylsilicate **2** has been carried out by direct addition of chloromethyllithium, generated in situ, to spirosilane **1**. This new pentacoordinate silicon derivative could be transformed into a new tetravalent spirosilane **5** presenting a six-membered ring. The ring expansion is promoted by magnesium(II) bromide which favors the insertion of the methylene into the Si–C_Ar_ bond and assists the chloride release as pointed out by DFT calculation. The presence of a Lewis base such as DMAP can also promote the ring expansion. A mechanism going through a hexacoordinate adduct was proposed and seemed to be computationally relevant even if no experimental evidence has yet been obtained. The new spirosilane **5** may present some interesting Lewis acid properties and its resulting pentacoordinate adducts may also exhibit interesting configurational stabilities [27]. These studies are ongoing in our laboratories.

## Figures and Tables

**Table 1:** Additions of various organolithiums to chloromethylsilicate **2**.

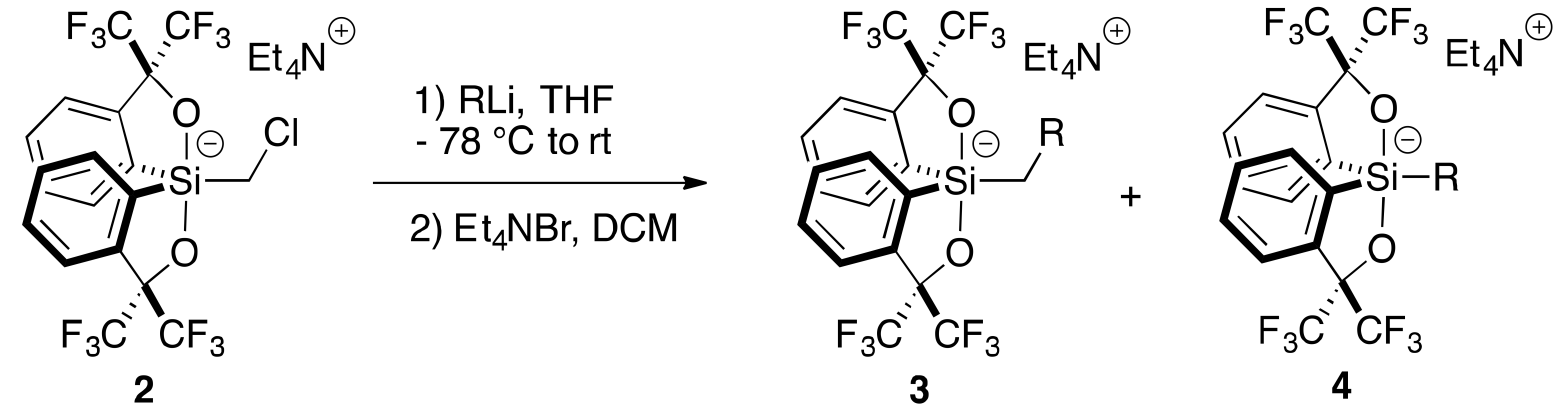			
Entry	RLi	Product (Ratio 3/4)	Overall Yield 3 + 4
1	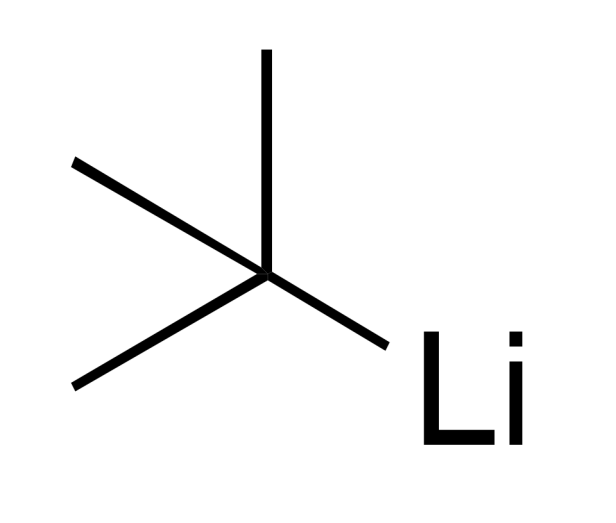	**3b**	74%
2	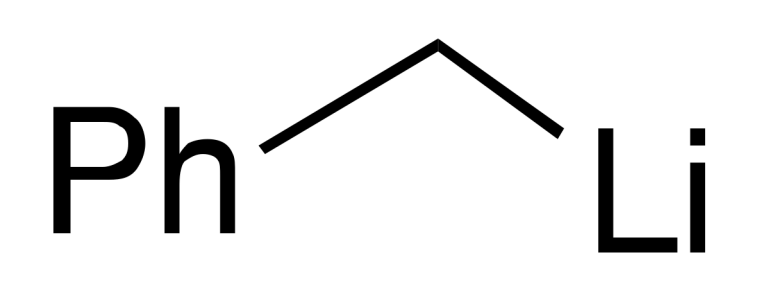	**3c**	73%
3	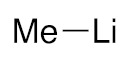	**3d**/**4d** (55/45)	36%
4	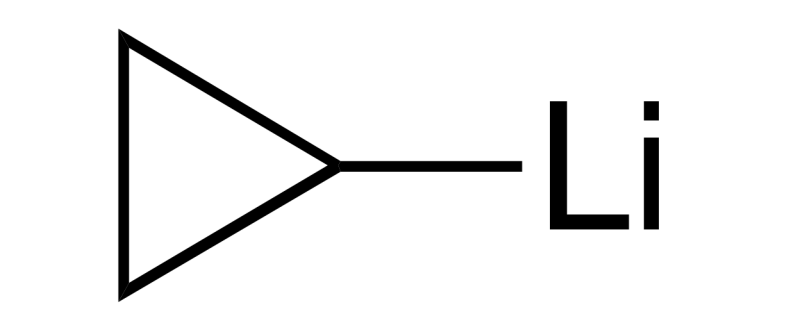	**3e**/**4e** (83/17)	81%
5	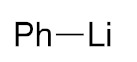	**3f**	61%
6	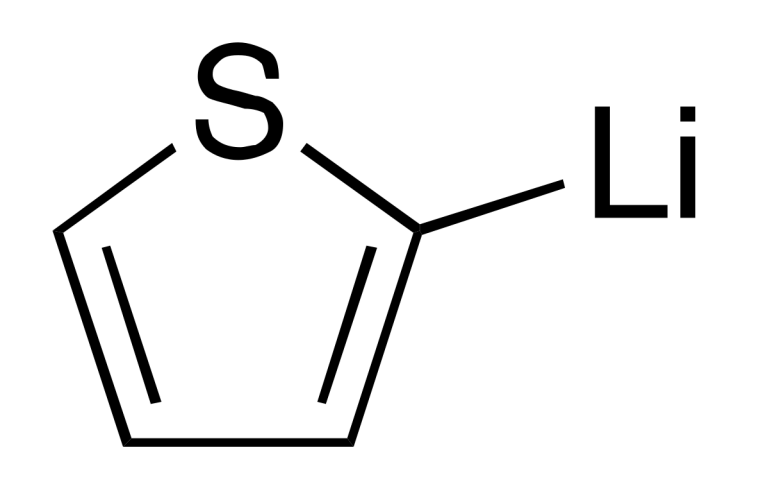	**3g**	69%

## Data Availability

Not applicable.

## Supplementary Materials

Supplementary part change to Copies of the NMR spectra and computational details are available in the online at https://www.mdpi.com/article/10.3390/molecules27061767/s1. Reference [28] is cited in the supplementary materials.
