# Supplementary material for: Synthesis and Reactivity of Martin’s Spirosilane-Derived Chloromethylsilicate
Source: Molecules. 2022 Mar 8;27(6):1767. doi: 10.3390/molecules27061767 (PMC8956079; doi:10.3390/molecules27061767)
Supplement: Supplementary file 1 [file molecules-27-01767-s001.zip › molecules-1605962-supplementary.pdf]

Supporting Information

Synthesis and Reactivity of Martin's Spirosilane-Derived Chloromethylsilicate

Thomas Deis [1], Jérémy Forte [1], Louis Fensterbank [1],* and Gilles Lemière [1],*

[1] Sorbonne Université, CNRS, Institut Parisien de Chimie Moléculaire, IPCM, 4 place Jussieu, F-75005 Paris, France

* Correspondence: louis.fensterbank@sorbonne-universite.fr, gilles.lemiere@sorbonne-universite.fr

I. Synthesis of organolithium reagents **3**
Synthesis of benzyllithium: 3
Synthesis of cyclopropyllithium: 3
Synthesis of 2-thienyllithium: 3
II. NMR monitoring following the method B **4**
Synthesis of hydroxysilicate 7: 4
Isolation and Stability's assessment of the zwitterionic silicate byproduct 8 upon heating 6
III. NMR Spectra **7**
^{1}H, ^{13}C, ^{19}F, and $^{1}H/^{29}Si$ HMQC NMR spectra of 2 in $(CD_3)_2CO$ 7
^{1}H, ^{13}C, ^{19}F, and $^{1}H/^{29}Si$ HMQC NMR spectra of 3b in $(CD_3)_2CO$ 9
^{1}H, ^{13}C, ^{19}F, and $^{1}H/^{29}Si$ HMQC NMR spectra of 3c in $(CD_3)_2CO$ 12
^{1}H, ^{13}C, ^{19}F, and $^{1}H/^{29}Si$ HMQC NMR spectra of mixture 3d/4d in $(CD_3)_2CO$ 14
^{1}H, ^{13}C, ^{19}F, and $^{1}H/^{29}Si$ HMQC NMR spectra of mixture 3e/4e in $(CD_3)_2CO$ and 4e in $(CD_3)_2CO$ 17
^{1}H, ^{13}C, ^{19}F, and $^{1}H/^{29}Si$ HMQC NMR spectra of 3f in $(CD_3)_2CO$ 21
^{1}H, ^{13}C, ^{19}F, and $^{1}H/^{29}Si$ HMQC NMR spectra of 3g in $(CD_3)_2CO$ 23
^{1}H, ^{13}C, ^{19}F, and $^{1}H/^{29}Si$ HMQC NMR spectra of 5 in $CDCl_3$ 26
^{1}H, ^{13}C, ^{19}F, and $^{1}H/^{29}Si$ HMQC NMR spectra of 7 in CD_3CN 29
^{1}H, ^{13}C, ^{19}F, and $^{1}H/^{29}Si$ HMQC NMR spectra of 7-Et_4N^+ in $(CD_3)_2CO$ 32
^{1}H, ^{13}C, ^{19}F, NMR spectra of 8 in $CDCl_3$ and $^{1}H/^{29}Si$ HMQC spectrum of 8 in CD_3CN 34
IV. Computational details **37**
V. Experimental references **49**

I. Synthesis of organolithium reagents

Synthesis of benzyllithium:

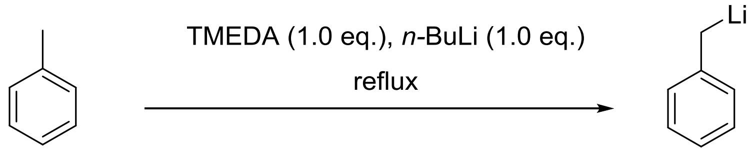

In a dry Schlenk flask was added TMEDA (45 µL, 0.30 mmol, 1.0 eq.) and toluene (0.24 mL, 0.30 mmol, 1.0 eq.). *n*-BuLi 2.5M in hexane (0.12 mL, 0.30 mmol, 1.0 eq.) was added dropwise at room temperature, and the reaction was refluxed for 1h. The resulting deep orange mixture was allowed to reach room temperature and was used immediately for the synthesis of phenylethylsilicate **3c**.

Synthesis of cyclopropyllithium:

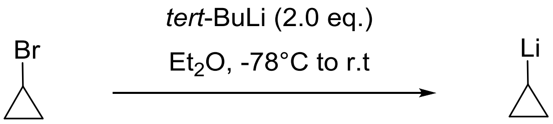

In a dry Schlenk flask was added cyclopropyl bromide (16 µL, 0.20 mmol, 1.0 eq.) and Et_2O (0.5 mL). The resulting solution was cooled down to -78°C and *tert*-BuLi 1.7 M in pentane (0.24 mL, 0.40 mmol, 2.0 eq.) was added dropwise and the reaction mixture was stirred at -78°C for 1h. The solution of cyclopropyllithium was used immediately for the synthesis of the mixture of cyclopropylmethylsilicate **3e** and cyclopropyllithium.

Synthesis of 2-thienyllithium:

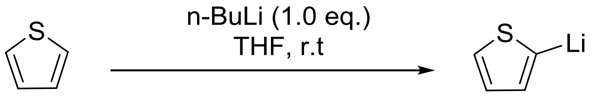

In a dry Schlenk flask was diluted thiophene (25 µL, 0.31 mmol, 1.0 eq.) in THF (0.5 mL), and *n*-BuLi 2.5 M in hexane (0.12 mL, 0.30 mmol, 1.0 eq.) was added dropwise at room temperature. The reaction mixture was stirred for 1h at room temperature. until the solution turns yellow. was used immediately for the synthesis of thienylmethylsilicate **3g**

II. NMR monitoring following the method B

Synthesis of hydroxysilicate 7:

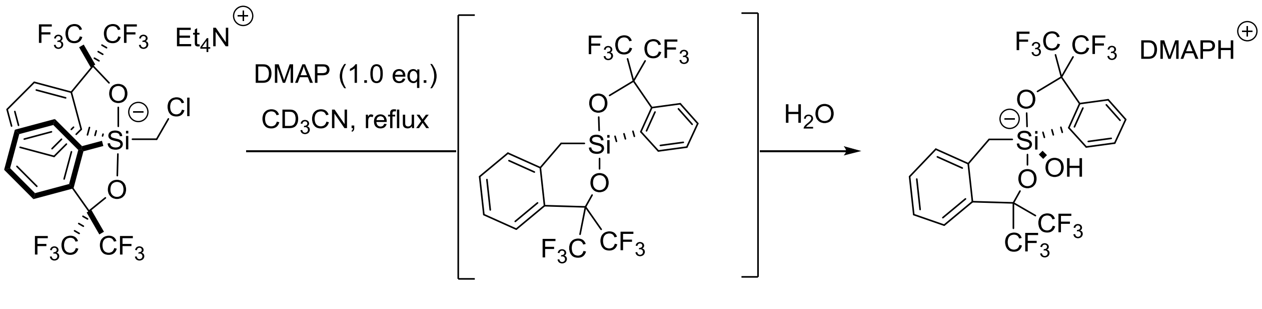

Synthesis of hydroxysilicate **7** according to the method B was NMR-monitored by means of a J. Young's tube starting from chloromethylsilicate **2** (20 mg, 0.029 mmol, 1.0 eq.) and DMAP (3.5 mg, 0.029 mmol, 1.0 eq.) in anhydrous acetonitrile-d_3 (0.4 mL). The reaction was heated at 80°C overnight, and then water was added to afford silicate **7**.

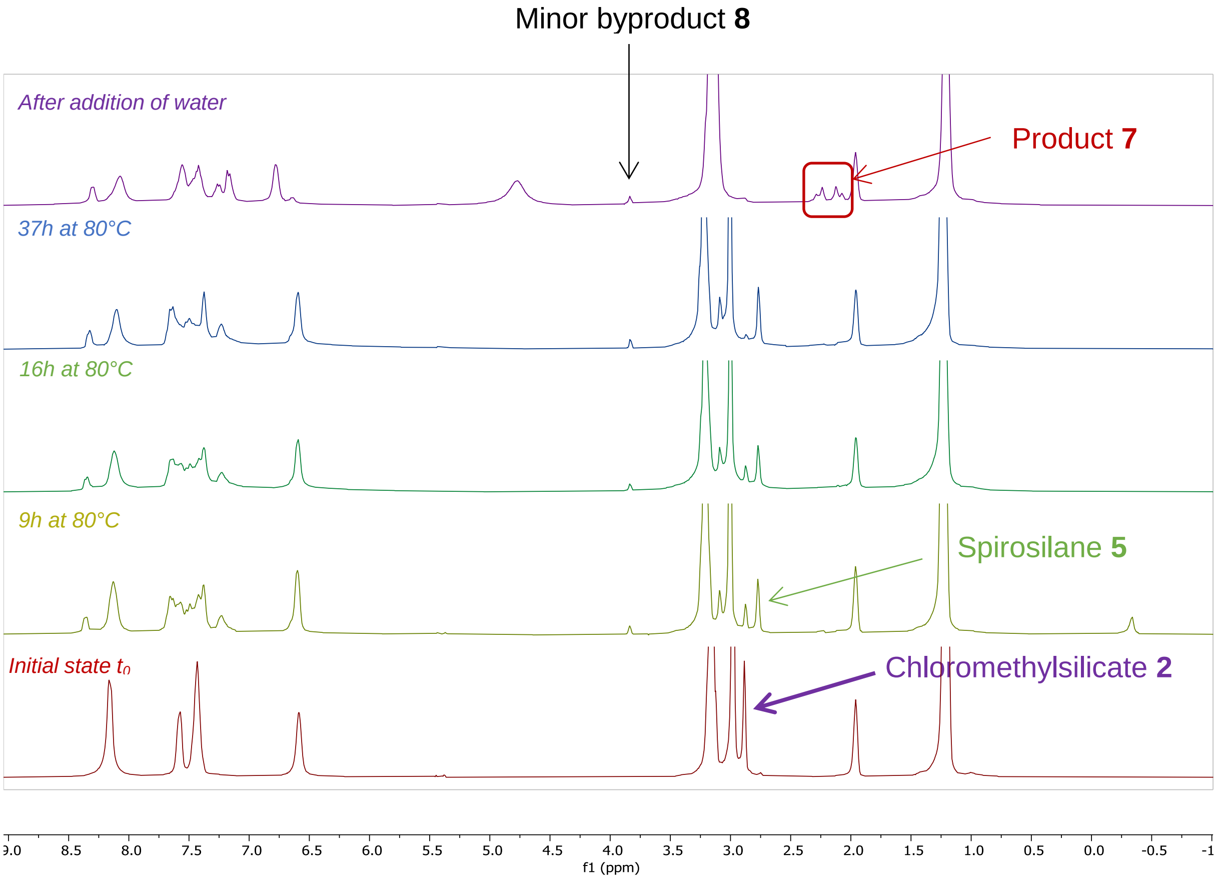

Figure S1 | ^{1}H NMR monitoring in CD_3CN for the reaction of chloromethylsilicate **2** with DMAP as Lewis base (method B).

Formation of spirosilane **5** results in appearance of a singlet (2.76 ppm) in CD_3CN associated to the benzylic protons. Air bubbling into the mixture affords hydroxysilicate **7**, which is highlighted by the AB system at 2.20 & 2.10 ppm. The singlet observed at 3.82 ppm indicated the presence of a minor byproduct, which was identified as the silicate **8** as a consequence of the direct substitution of chlorine atom of **2** by DMAP.

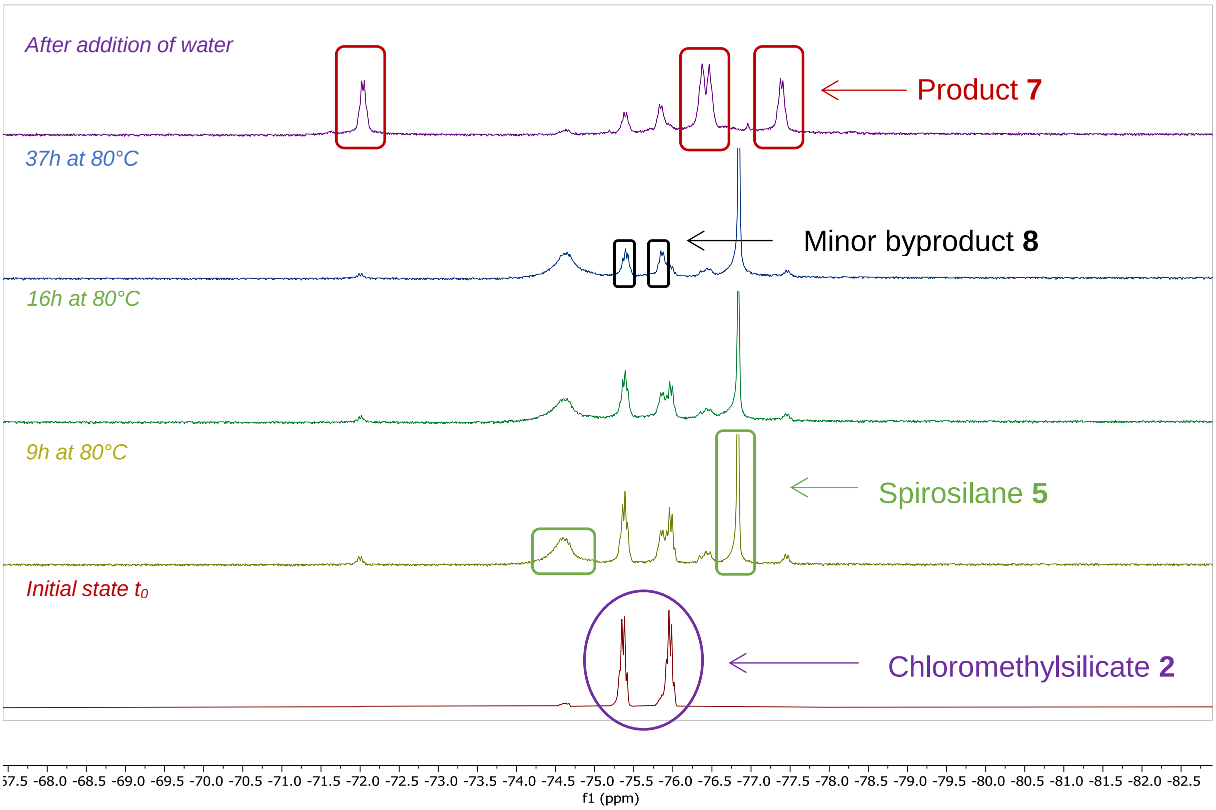

Figure S2 | ^{19}F NMR monitoring in CD_3CN for the reaction of chloromethylsilicate **2** with DMAP as Lewis base (method B).

Due to the loss of symmetry caused by the ring expansion, the transformation of chloromethylsilicate **2** (purple ellipse) to spirosilane **5** (green box) could be monitored by ^{19}F NMR with the apparition of the 1 broad signal at -74.6 and the singlet at -76.9 ppm. Air bubbling into the mixture triggers the transformation of **5** to hydroxysilicate **7** (red box), as highlighted by the appearance of the four quadruplets (-72.1, -76.4, -76.5, -77.4 ppm).

Isolation and Stability's assessment of the zwitterionic silicate byproduct 8 upon heating

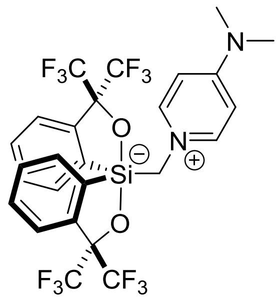

8

Zwiterrionic nature of byproduct **8** allowed its isolation by chromatography over SiO_2 (DCM 100%) as a colorless solid (1.9 mg, 9%).

^{1}H NMR: (400 MHz, $CDCl_3$) δ 8.11 (d, *J* = 6.9 Hz, 2H), 7.62 (m, 4H), 6.51 (d, *J* = 7.3 Hz, 2H), 3.98 (d, *J* = 14.8 Hz, 1H), 3.88 (d, *J* = 14.8 Hz, 1H), 3.19 (s, 6H). **^{13}C NMR**: (101 MHz, $CDCl_3$) δ 155.1, 143.3, 141.0, 140.4, 137.5, 129.4, 128.9, 123.8, 106.3, 56.4, 39.9, 29.7, 1.1. **^{19}F NMR**: (376 MHz, $CDCl_3$) δ -74.77 (q, *J* = 9.2 Hz), -75.33 (q, *J* = 9.4 Hz). **^{1}H/^{29}Si HMQC**: (400 MHZ/79 MHz, CD_3CN) 3.85/-74.7. **HRMS (ESI$^+$)**: m/z calcd for $C_{26}H_{20}F_{12}N_2O_2SiH$ [M+H] 649.1175, found 649.1171.

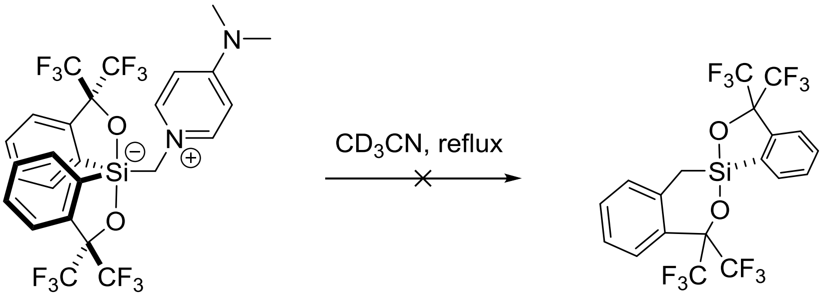

8 **5**

Thermal stability of silicate **8** was assessed by means of ^{1}H and ^{19}F NMR: Silicate **8** was added to a dried J. Young's tube with anhydrous acetonitrile-d_3, and the mixture was refluxed overnight. No reaction occurred.

III. NMR Spectra

^{1}H, ^{13}C, ^{19}F, and $^{1}H/^{29}Si$ HMQC NMR spectra of 2 in $(CD_3)_2CO$

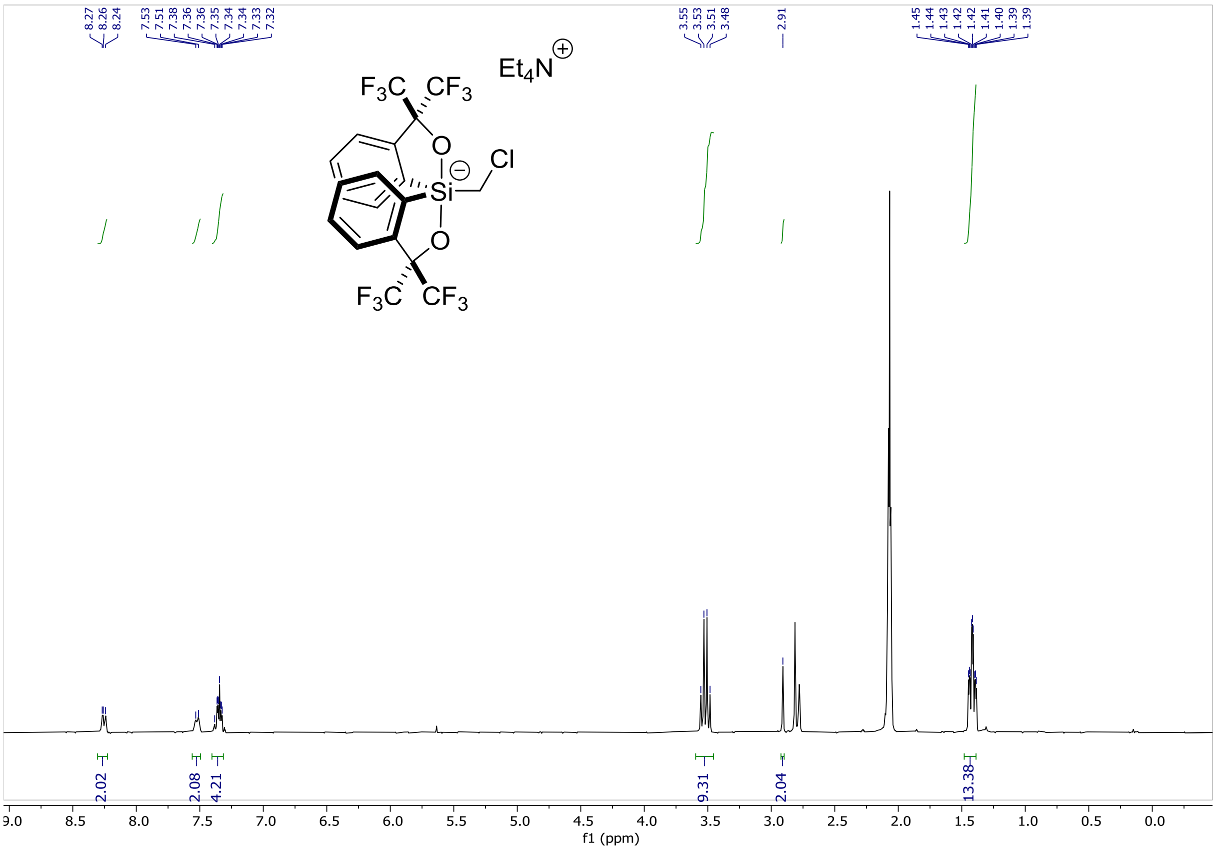

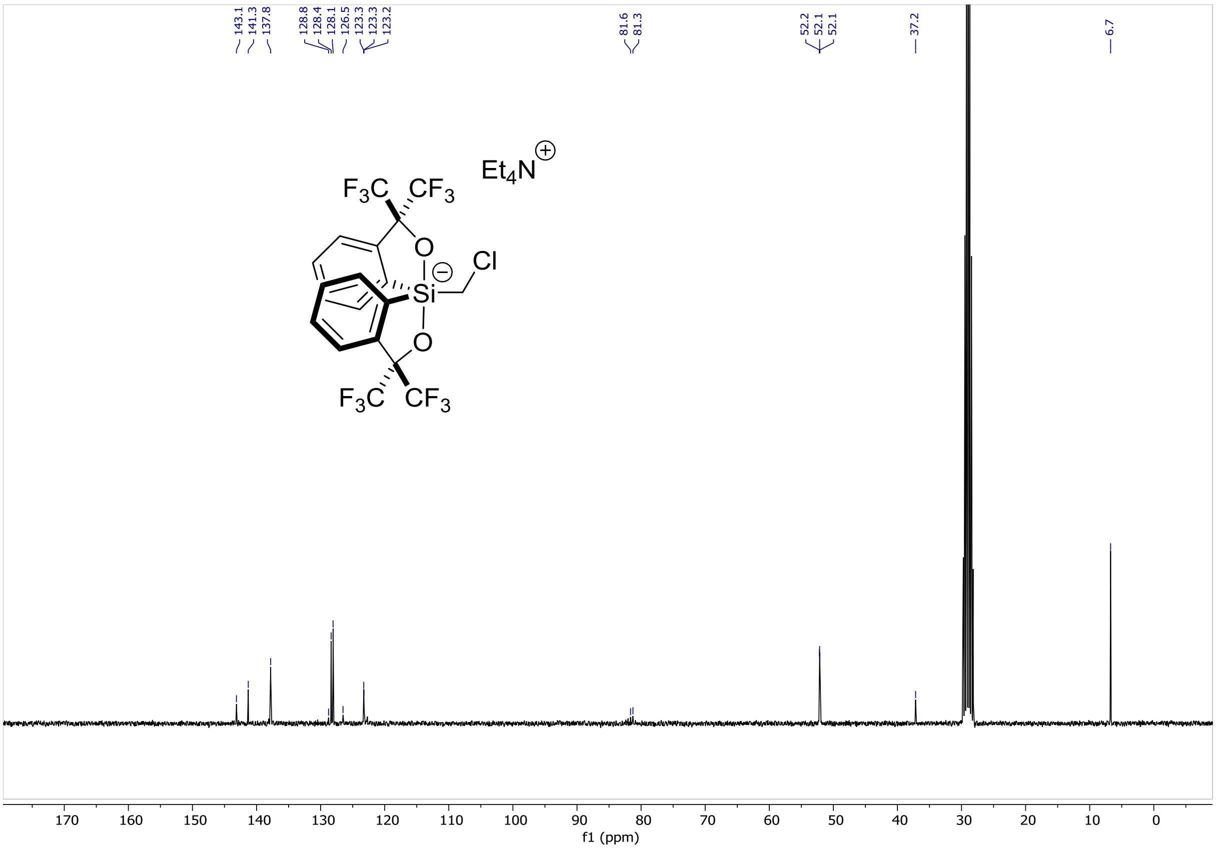
143.1
141.3
137.8
128.8
128.4
128.1
126.5
123.3
123.3
123.2
81.6
81.3
52.2
52.1
52.1
37.2
6.7
Et4N
F3C
CF3
O
Si
Cl
O
F3C
CF3
170
160
150
140
130
120
110
100
90
80
70
60
50
40
30
20
10
0
f1 (ppm)

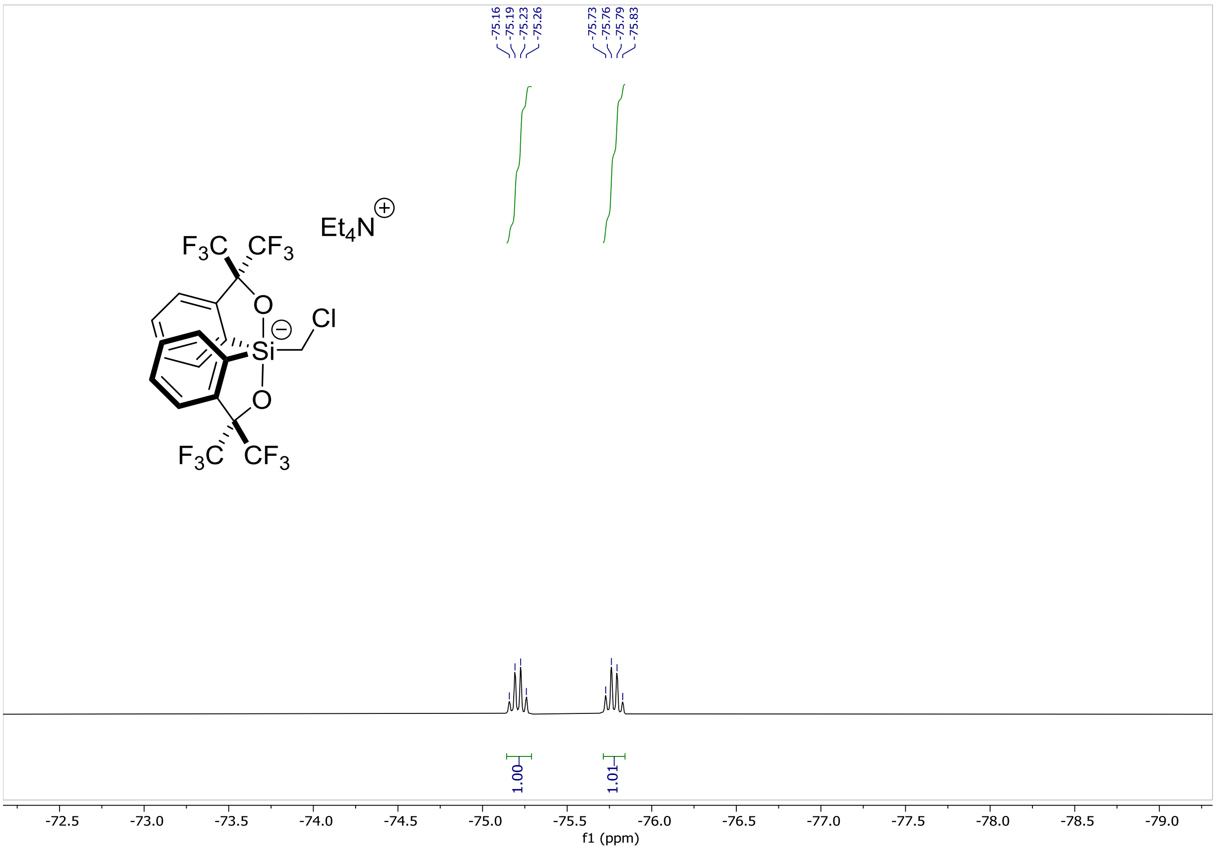
-75.16
-75.19
-75.23
-75.26
-75.73
-75.76
-75.79
-75.83
Et4N
F3C
CF3
O
Si
Cl
O
F3C
CF3
1.00
1.01
-72.5
-73.0
-73.5
-74.0
-74.5
-75.0
-75.5
-76.0
-76.5
-77.0
-77.5
-78.0
-78.5
-79.0
f1 (ppm)

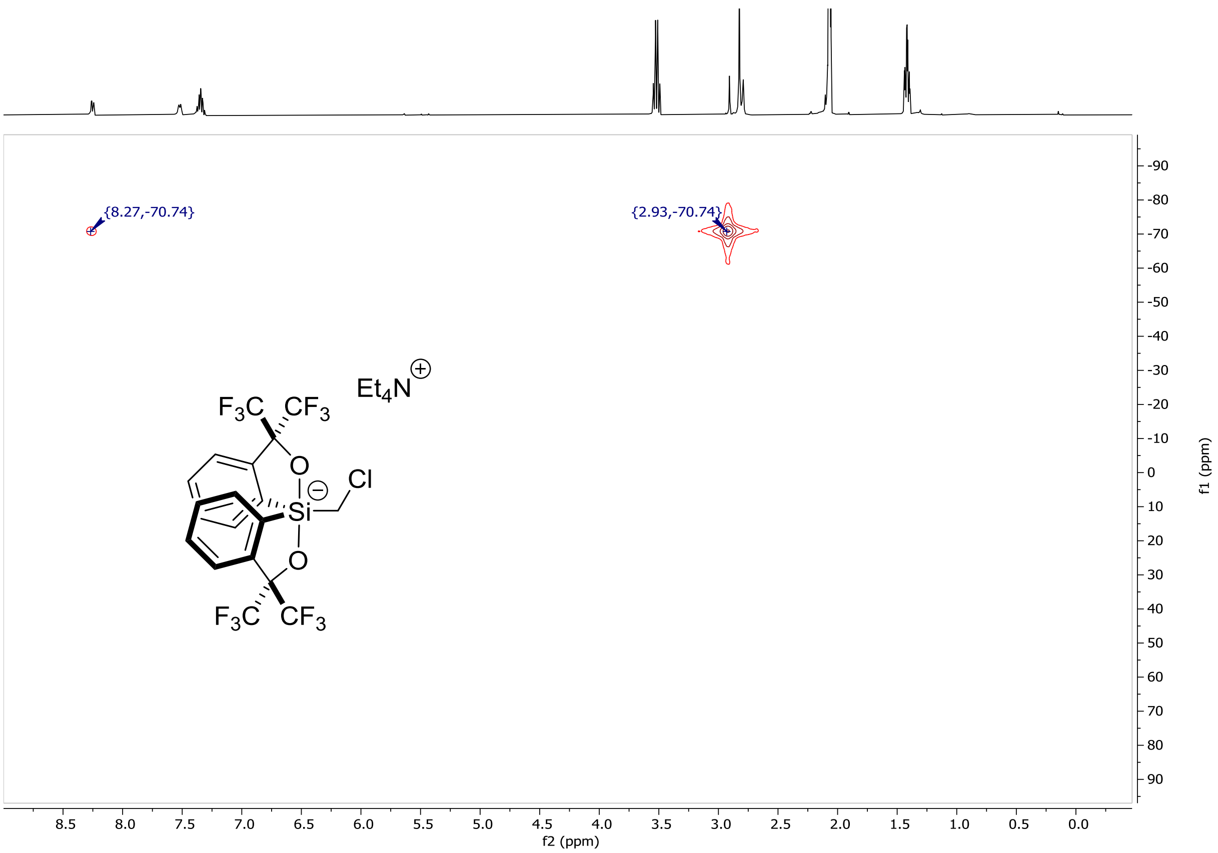

^{1}H, ^{13}C, ^{19}F, and $^{1}H/^{29}Si$ HMQC NMR spectra of 3b in $(CD_3)_2CO$

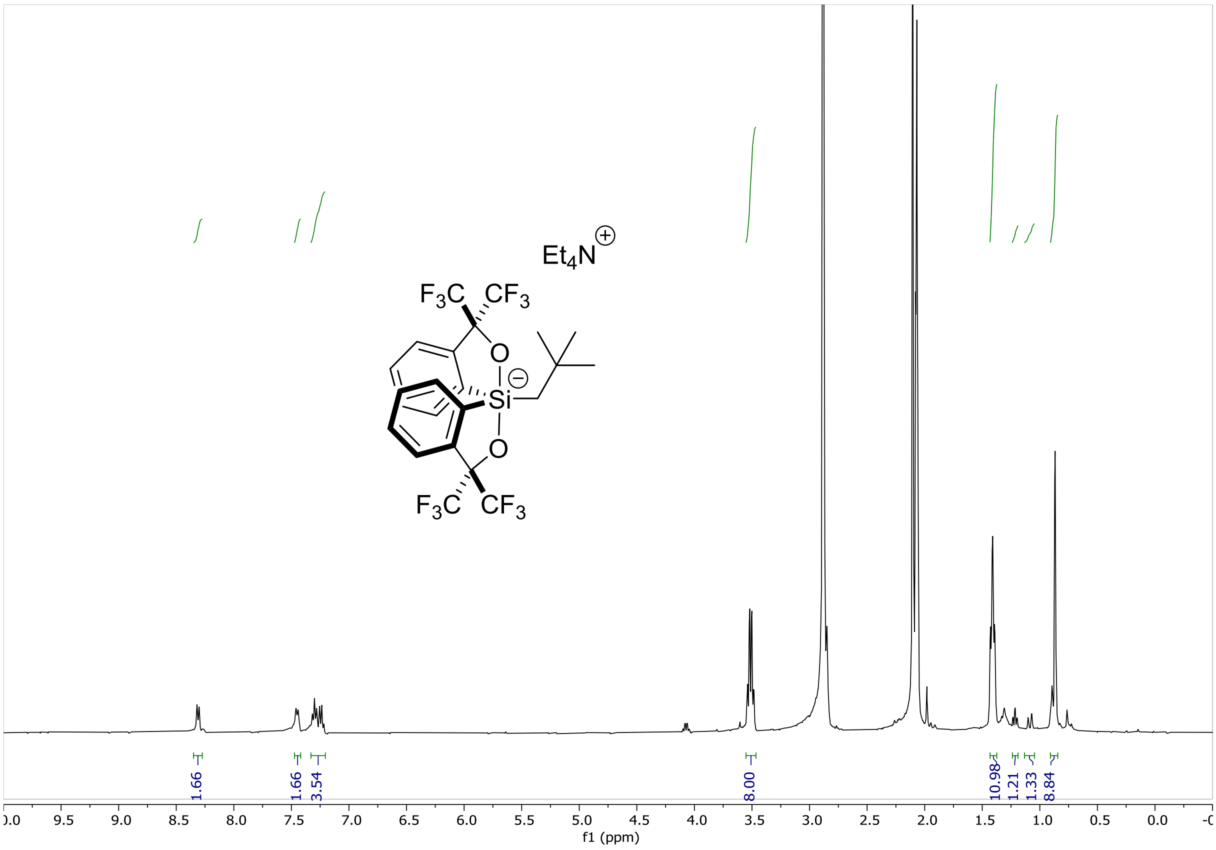
Et4N
F3C CF3
O
Si
O
F3C CF3
1.66
1.66
3.54
8.00
10.98
1.21
1.33
8.84
f1 (ppm)

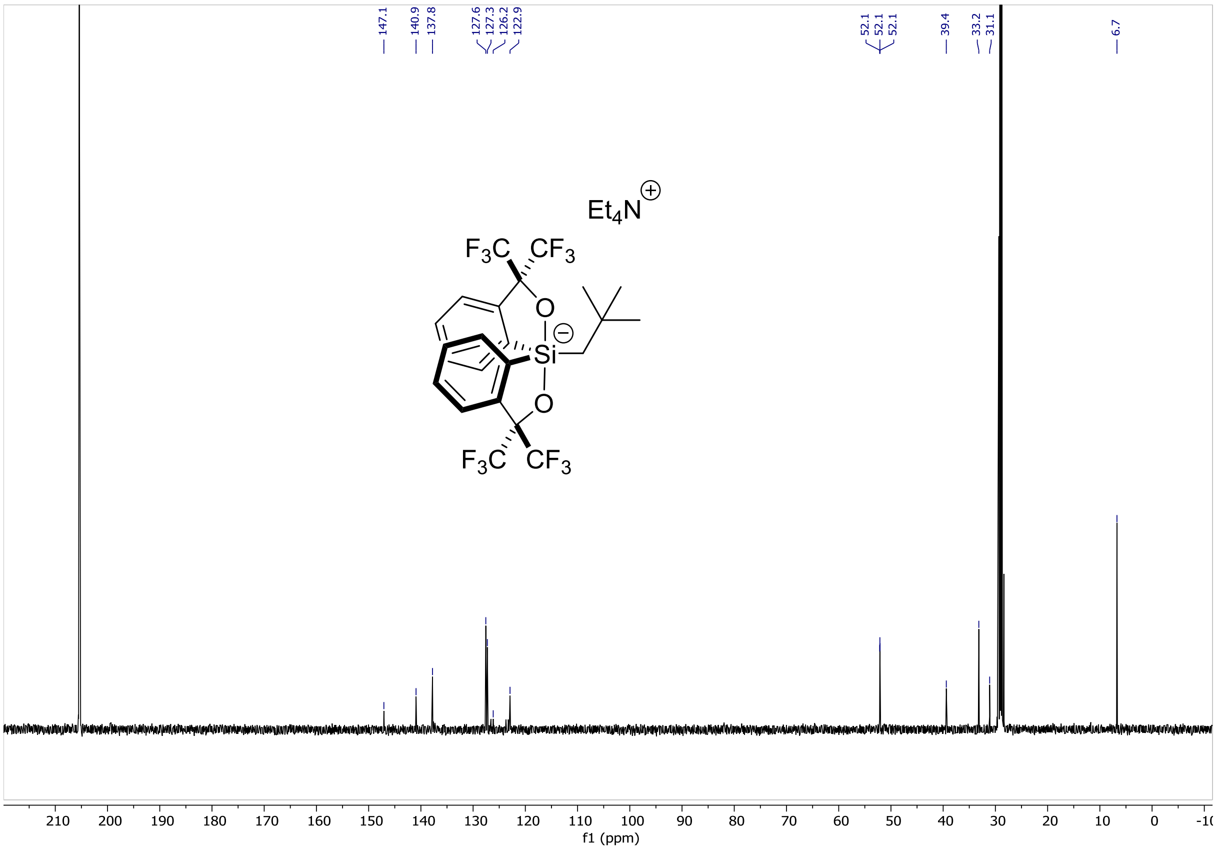
147.1
140.9
137.8
127.6
127.3
126.2
122.9
52.1
52.1
52.1
39.4
33.2
31.1
6.7
Et4N
F3C CF3
O
Si
O
F3C CF3
f1 (ppm)

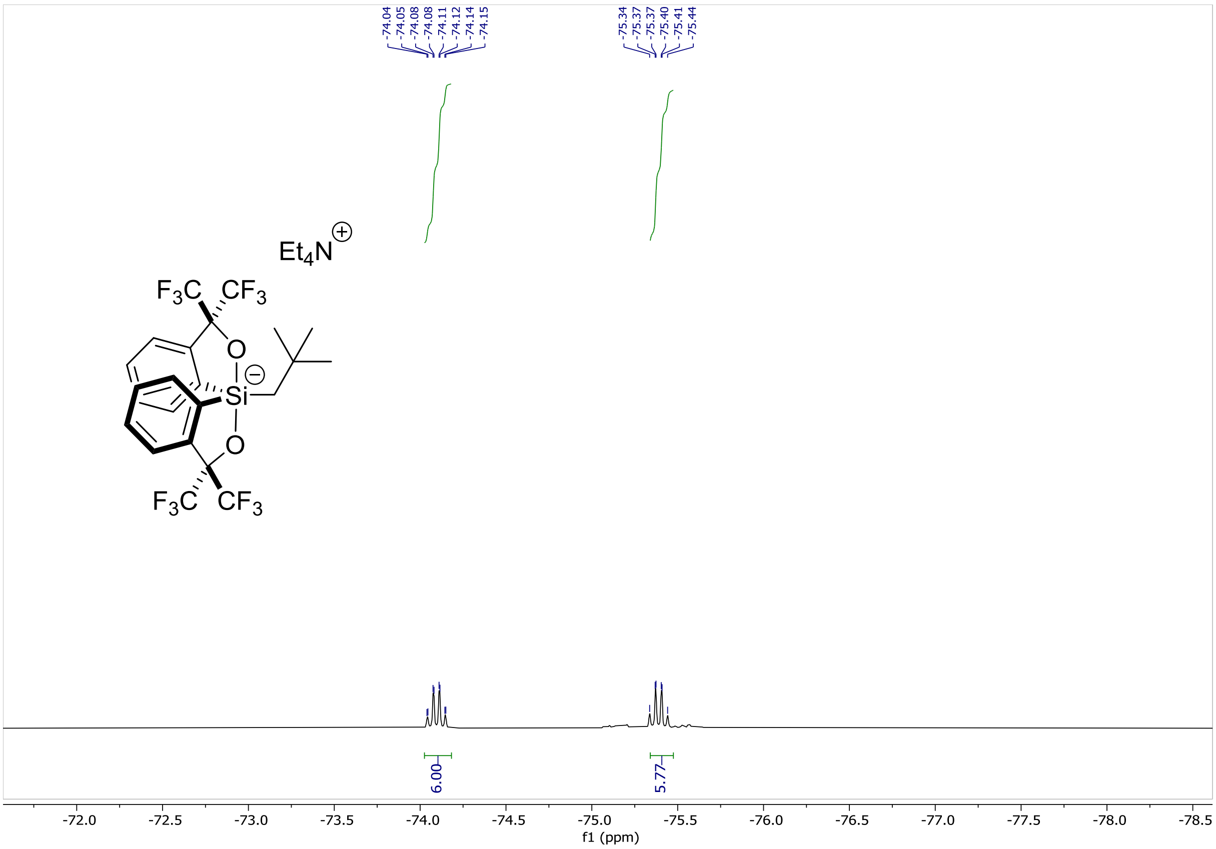
-74.04
-74.05
-74.08
-74.08
-74.11
-74.12
-74.14
-74.15
-75.34
-75.37
-75.37
-75.40
-75.41
-75.44
Et4N
F3C CF3
O
Si
O
F3C CF3
6.00
5.77
-72.0
-72.5
-73.0
-73.5
-74.0
-74.5
-75.0
-75.5
-76.0
-76.5
-77.0
-77.5
-78.0
-78.5
f1 (ppm)

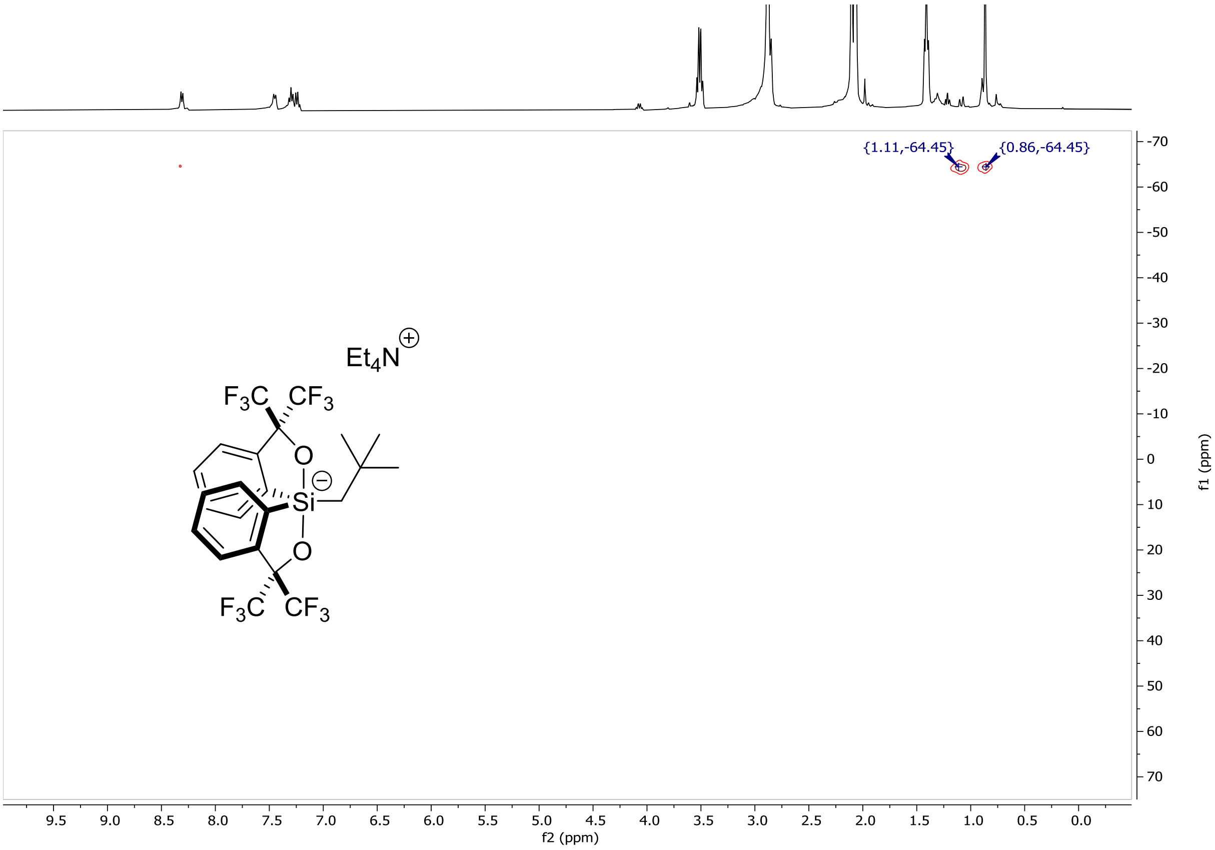
{1.11,-64.45}
{0.86,-64.45}
Et4N
F3C CF3
O
Si
O
F3C CF3
-70
-60
-50
-40
-30
-20
-10
0
10
20
30
40
50
60
70
f1 (ppm)
9.5
9.0
8.5
8.0
7.5
7.0
6.5
6.0
5.5
5.0
4.5
4.0
3.5
3.0
2.5
2.0
1.5
1.0
0.5
0.0
f2 (ppm)

^{1}H, ^{13}C, ^{19}F, and ^{1}H/^{29}Si HMQC NMR spectra of 3c in $(CD_3)_2CO$

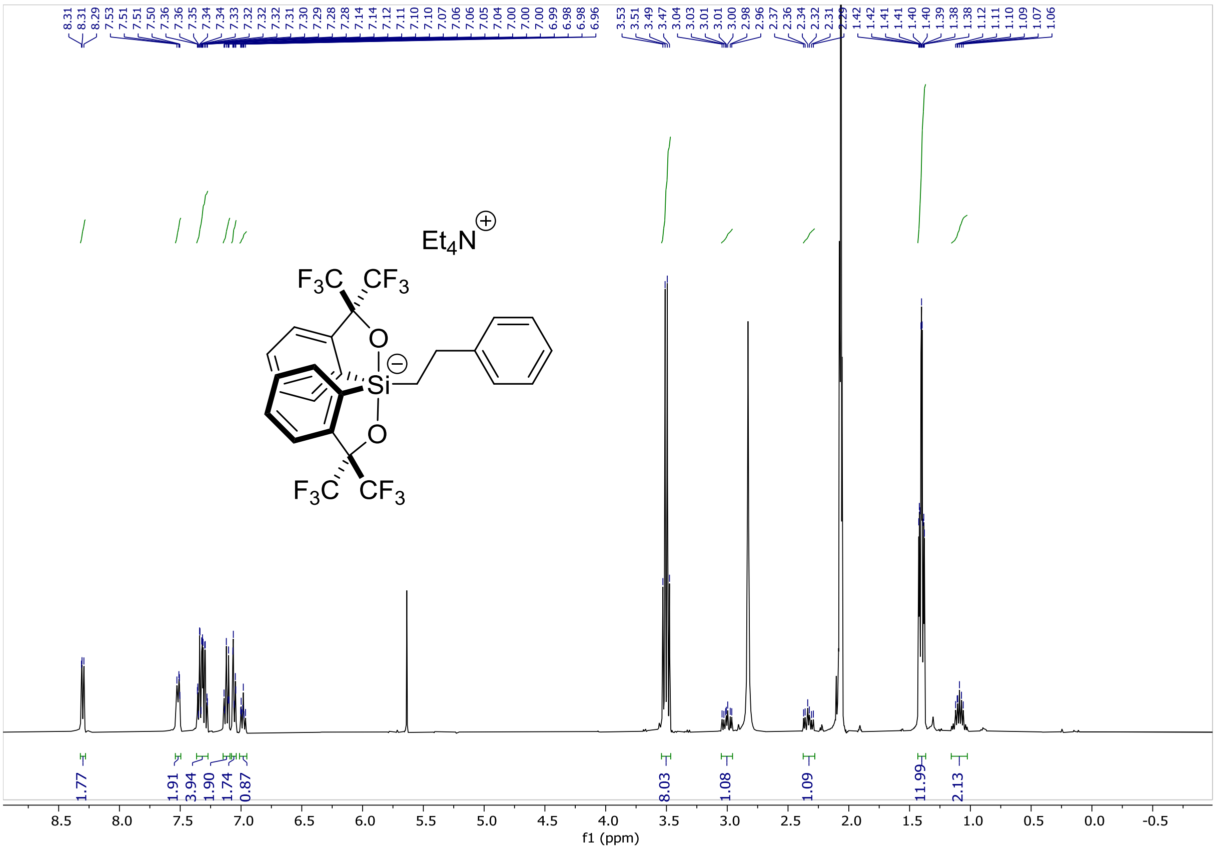

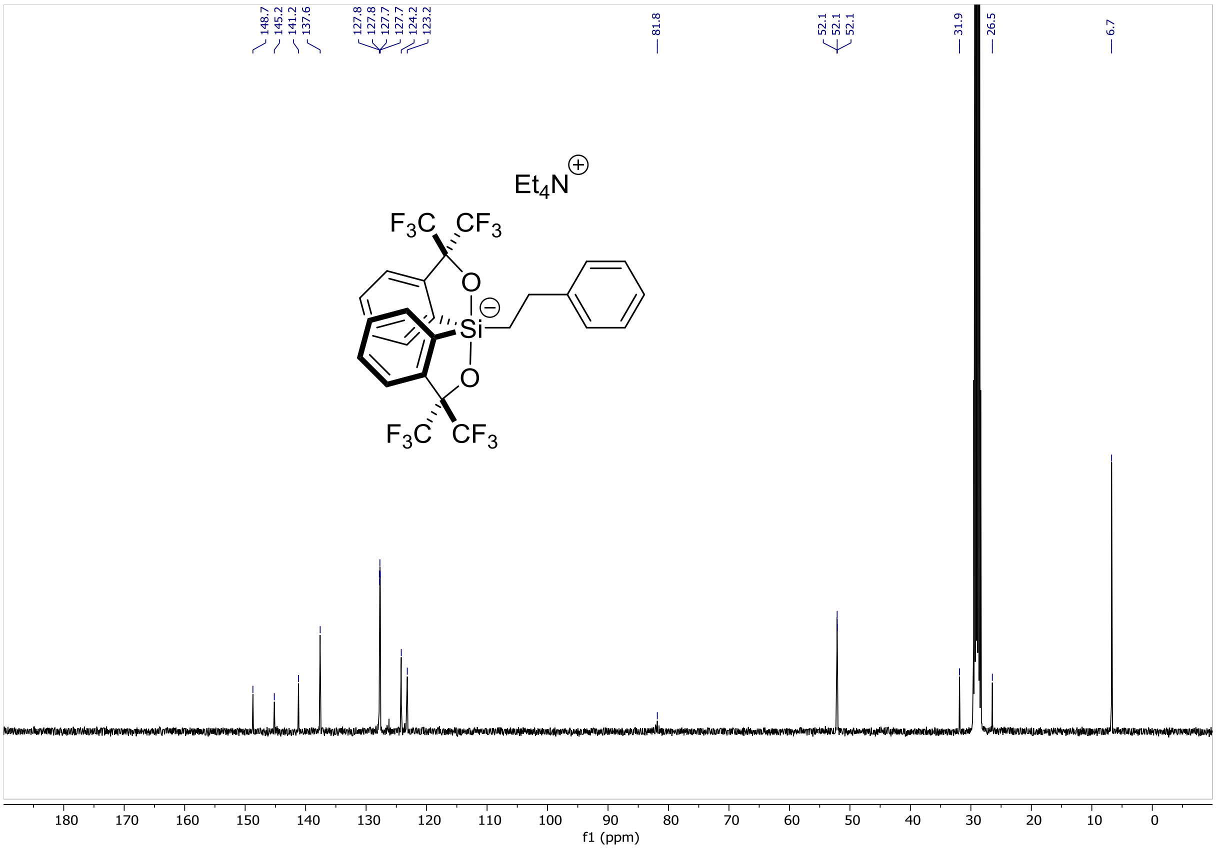

148.7
145.2
141.2
137.6
127.8
127.8
127.7
127.7
124.2
123.2
81.8
52.1
52.1
52.1
31.9
26.5
6.7
Et4N
F3C
CF3
O
Si
O
F3C
CF3
180
170
160
150
140
130
120
110
100
90
80
70
60
50
40
30
20
10
0
f1 (ppm)

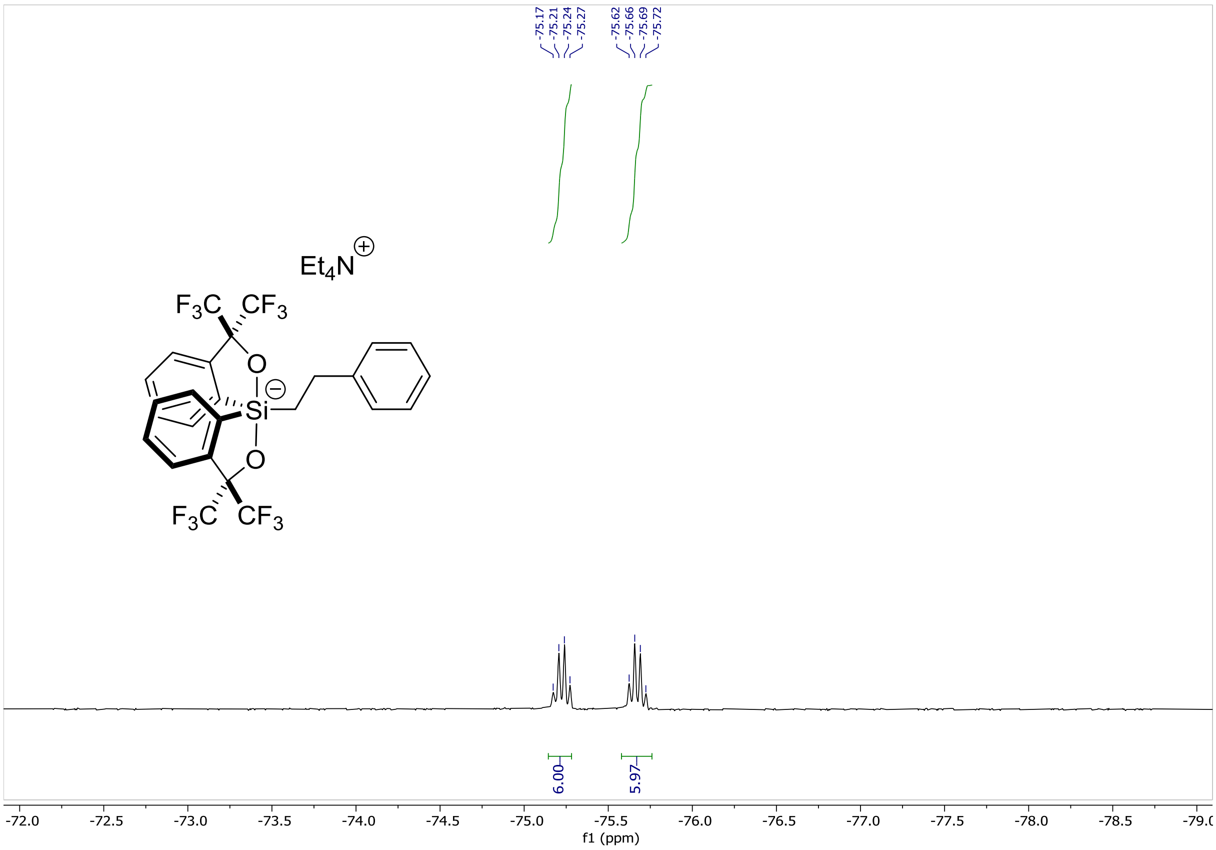

-75.17
-75.21
-75.24
-75.27
-75.62
-75.66
-75.69
-75.72
Et4N
F3C
CF3
O
Si
O
F3C
CF3
6.00
5.97
-72.0
-72.5
-73.0
-73.5
-74.0
-74.5
-75.0
-75.5
-76.0
-76.5
-77.0
-77.5
-78.0
-78.5
f1 (ppm)

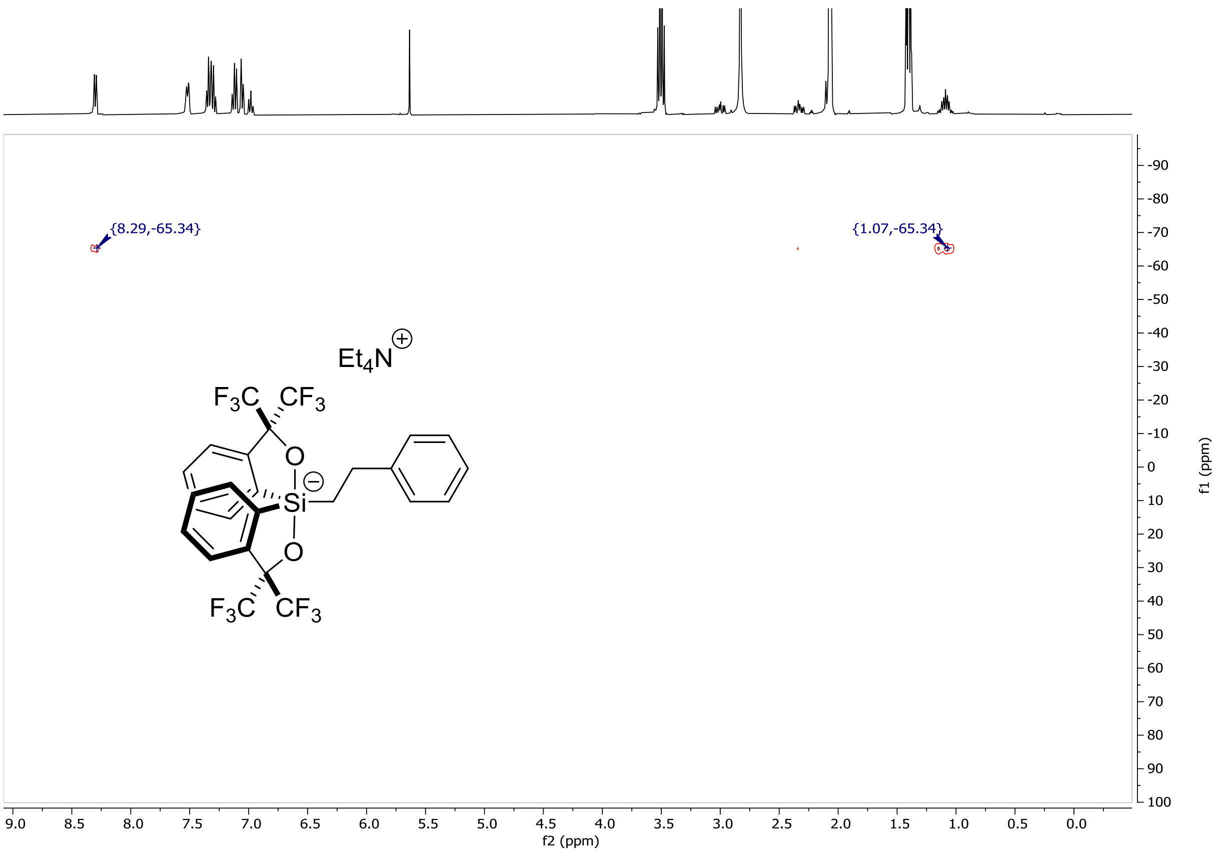

^{1}H, ^{13}C, ^{19}F, and $^{1}H/^{29}Si$ HMQC NMR spectra of mixture 3d/4d in $(CD_3)_2CO$

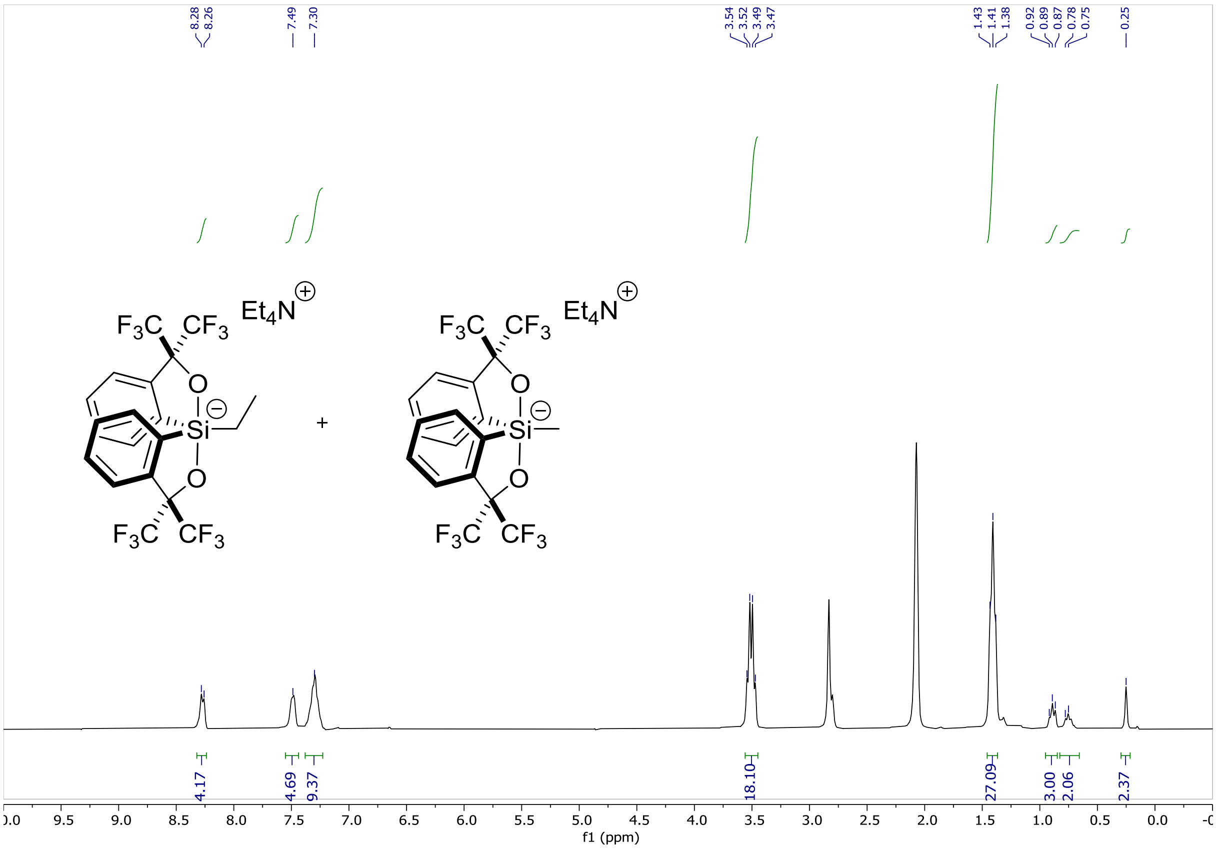
8.28
8.26
7.49
7.30
3.54
3.52
3.49
3.47
1.43
1.41
1.38
0.92
0.89
0.87
0.78
0.75
0.25
Et4N
F3C
CF3
O
Si
O
F3C
CF3
+
Et4N
F3C
CF3
O
Si
O
F3C
CF3
4.17
4.69
9.37
18.10
27.09
3.00
2.06
2.37
f1 (ppm)

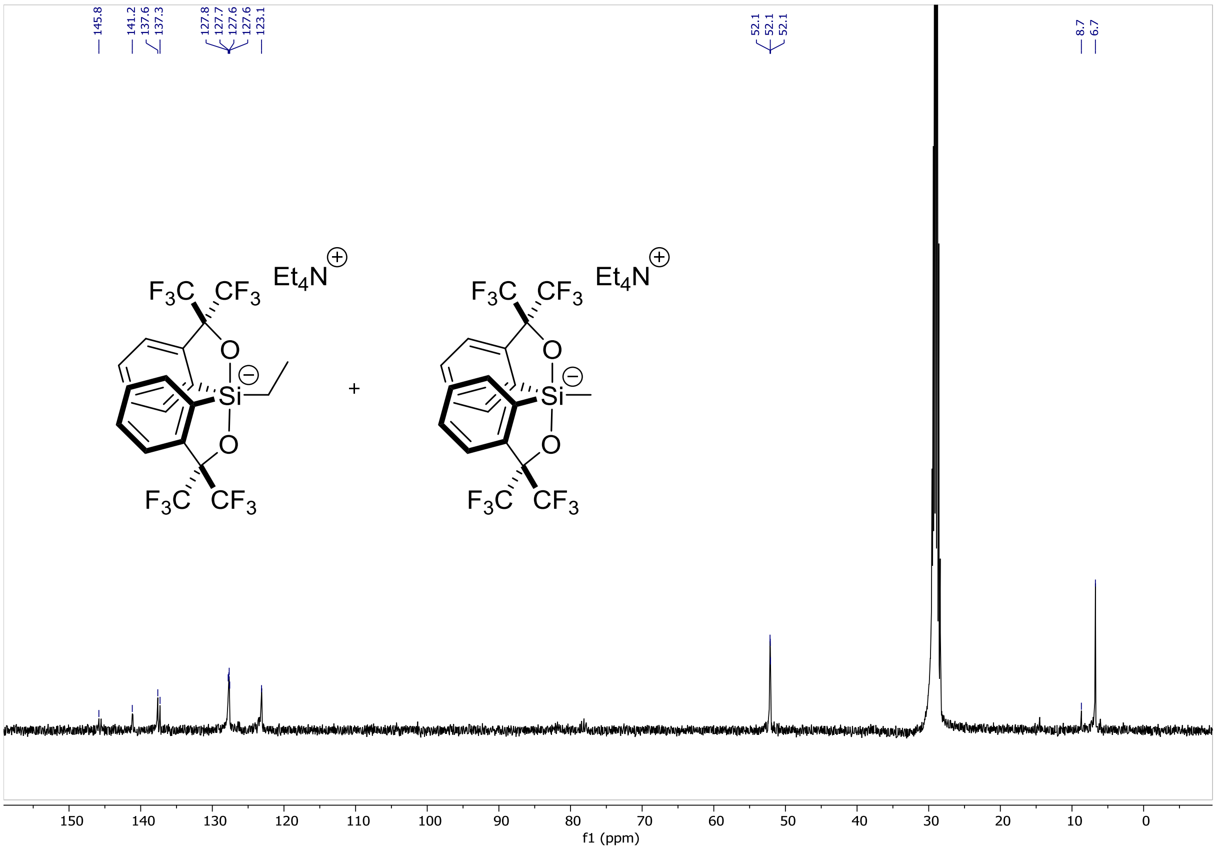
145.8
141.2
137.6
137.3
127.8
127.7
127.6
127.6
123.1
52.1
52.1
52.1
8.7
6.7
Et4N
F3C
CF3
O
Si
O
F3C
CF3
+
Et4N
F3C
CF3
O
Si
O
F3C
CF3
f1 (ppm)

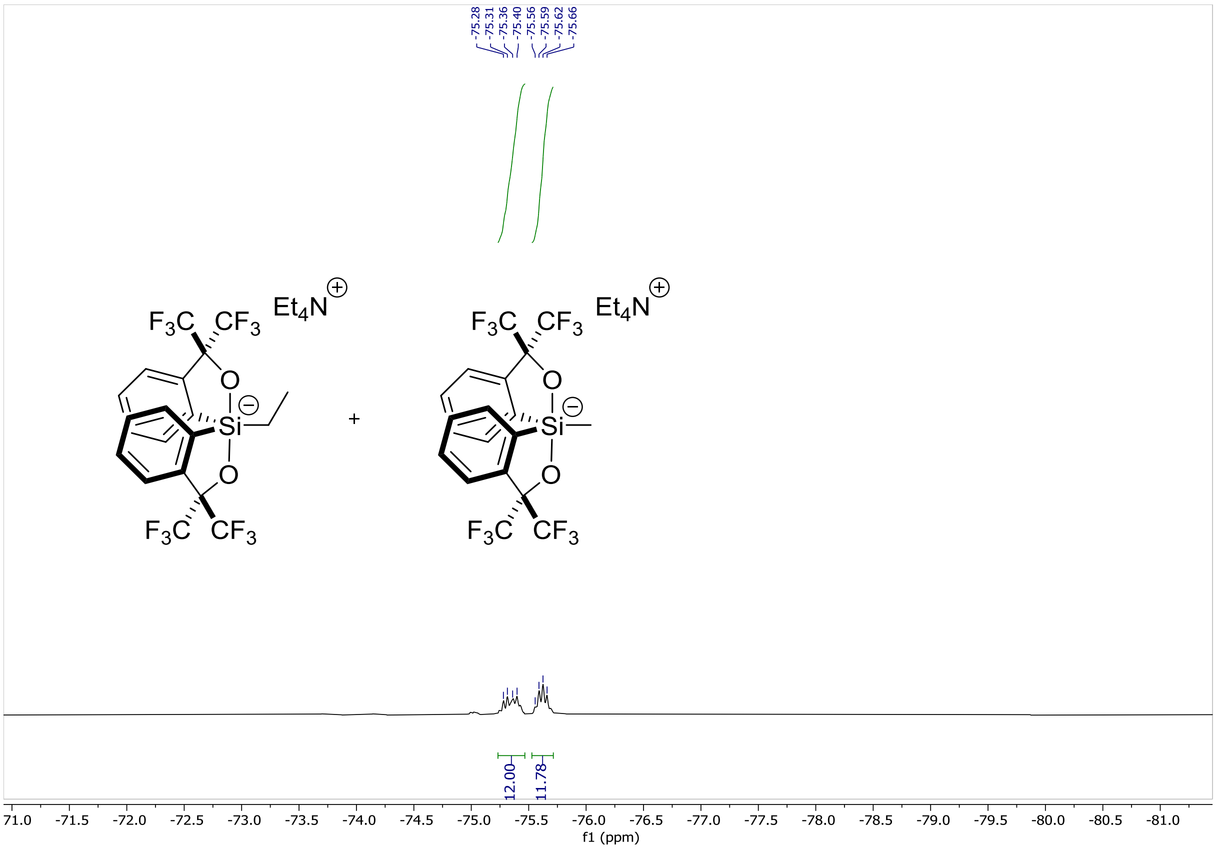
-75.28
-75.31
-75.36
-75.40
-75.56
-75.59
-75.62
-75.66
Et4N
F3C CF3
O
Si
O
F3C CF3
+
12.00
11.78
f1 (ppm)

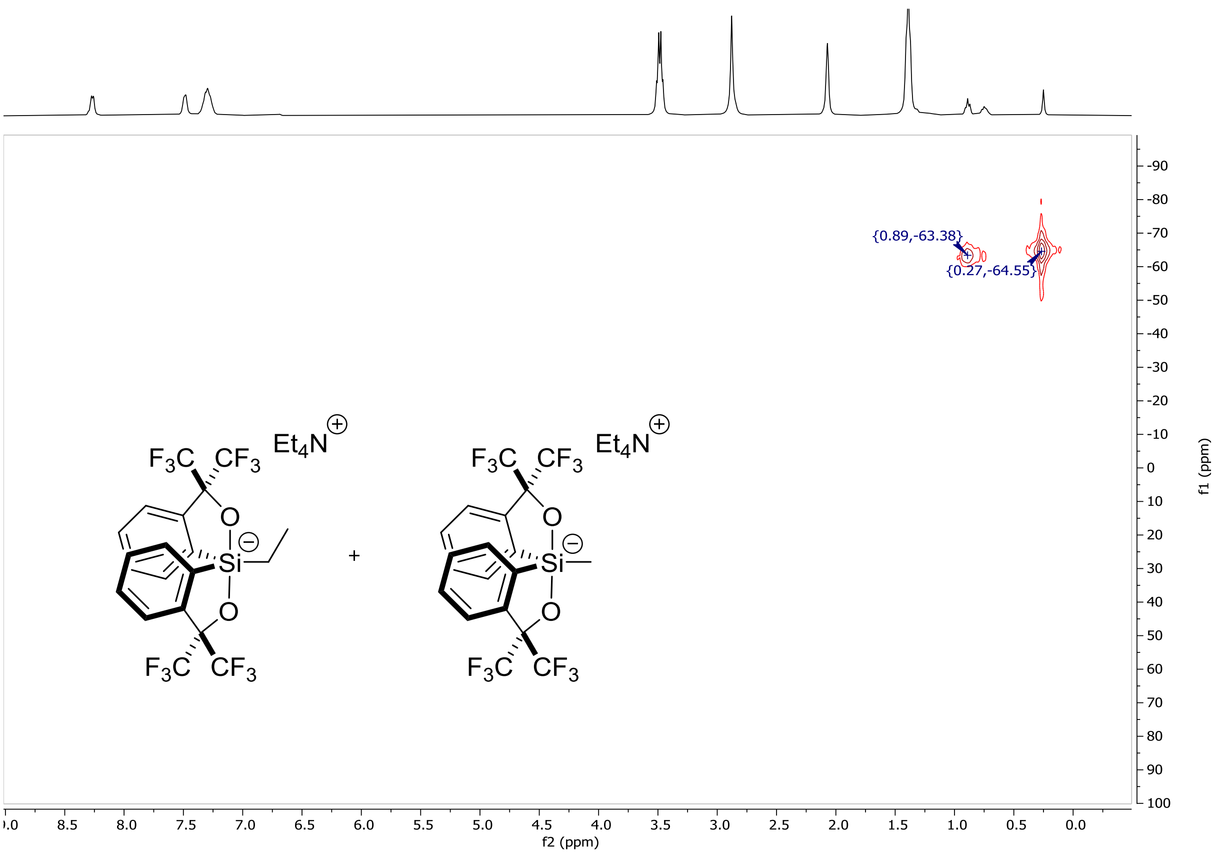
{0.89,-63.38}
{0.27,-64.55}
Et4N
F3C CF3
O
Si
O
F3C CF3
+
f2 (ppm)
f1 (ppm)

^{1}H, ^{13}C, ^{19}F, and $^{1}H/^{29}Si$ HMQC NMR spectra of mixture 3e/4e in $(CD_3)_2CO$ and 4e in $(CD_3)_2CO$

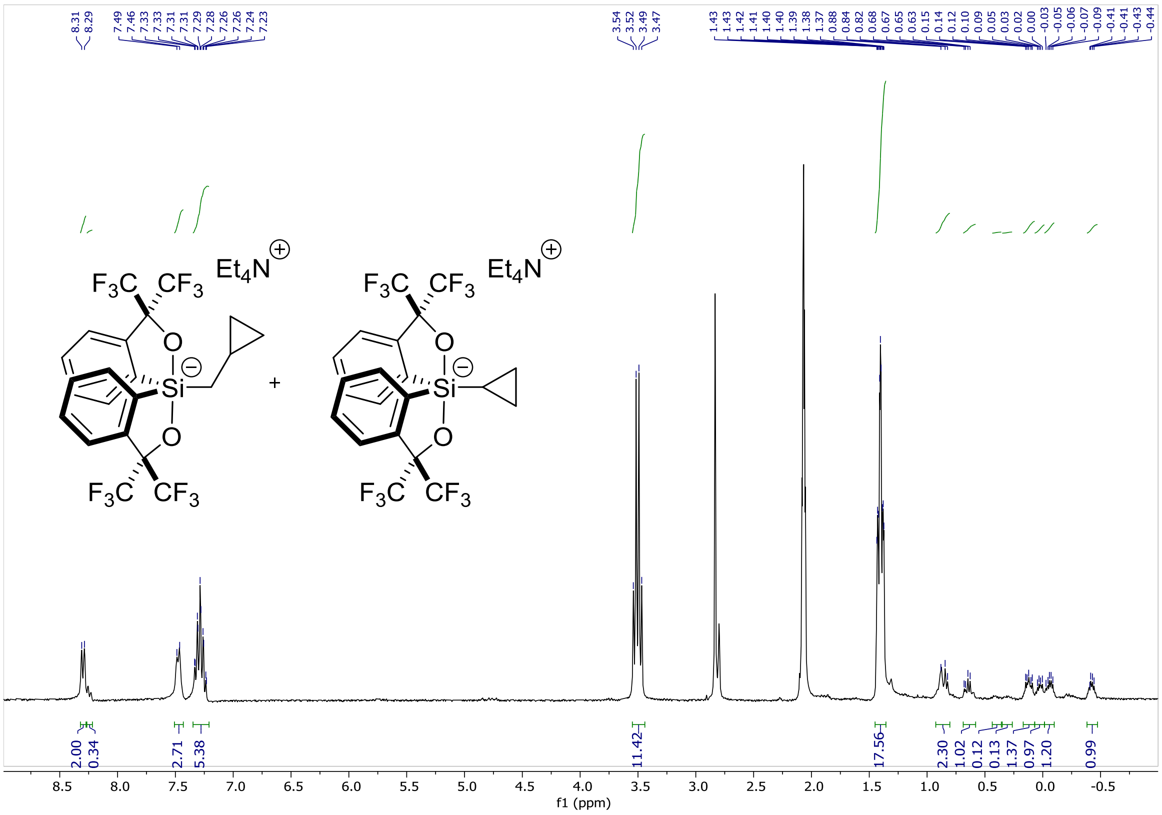

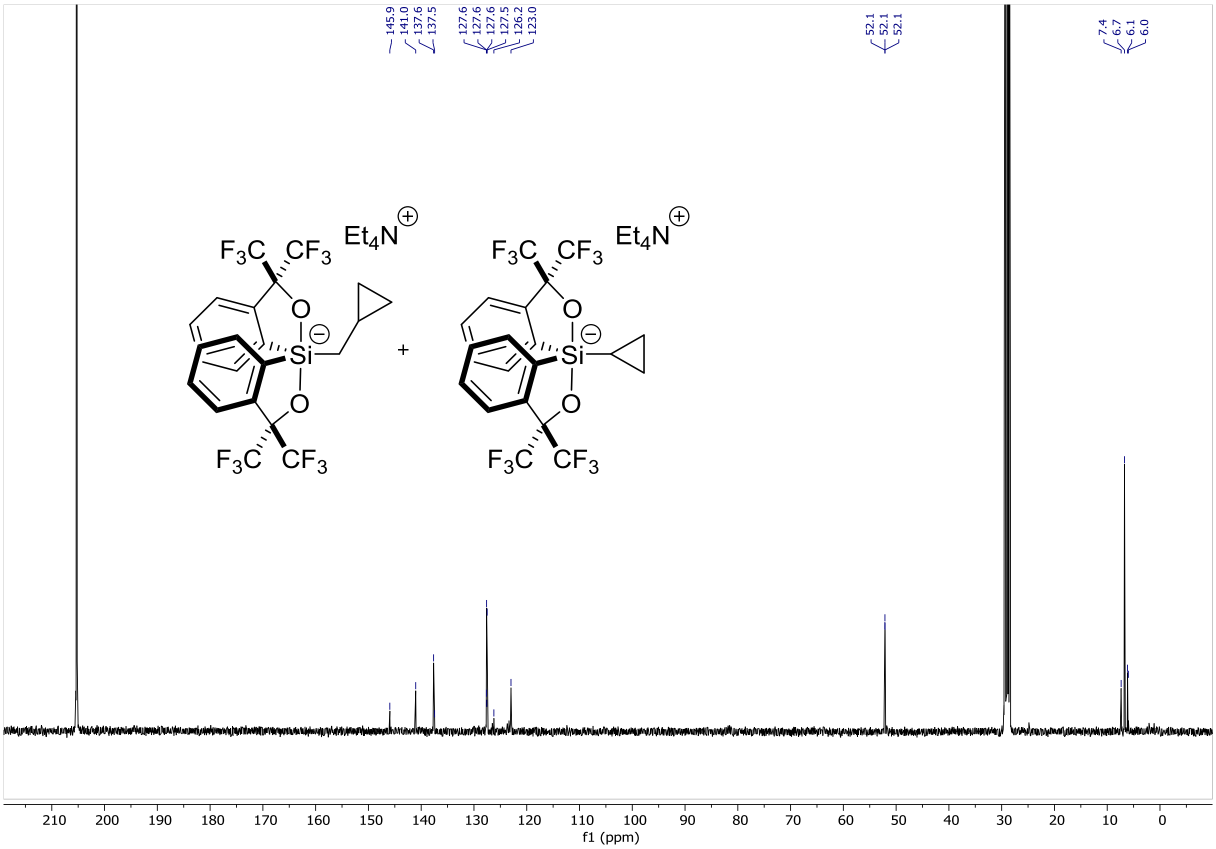
145.9
141.0
137.6
137.5
127.6
127.6
127.6
127.5
126.2
123.0
52.1
52.1
52.1
7.4
6.7
6.1
6.0
F3C CF3 Et4N
Si
O
F3C CF3
+
210 200 190 180 170 160 150 140 130 120 110 100 90 80 70 60 50 40 30 20 10 0
f1 (ppm)

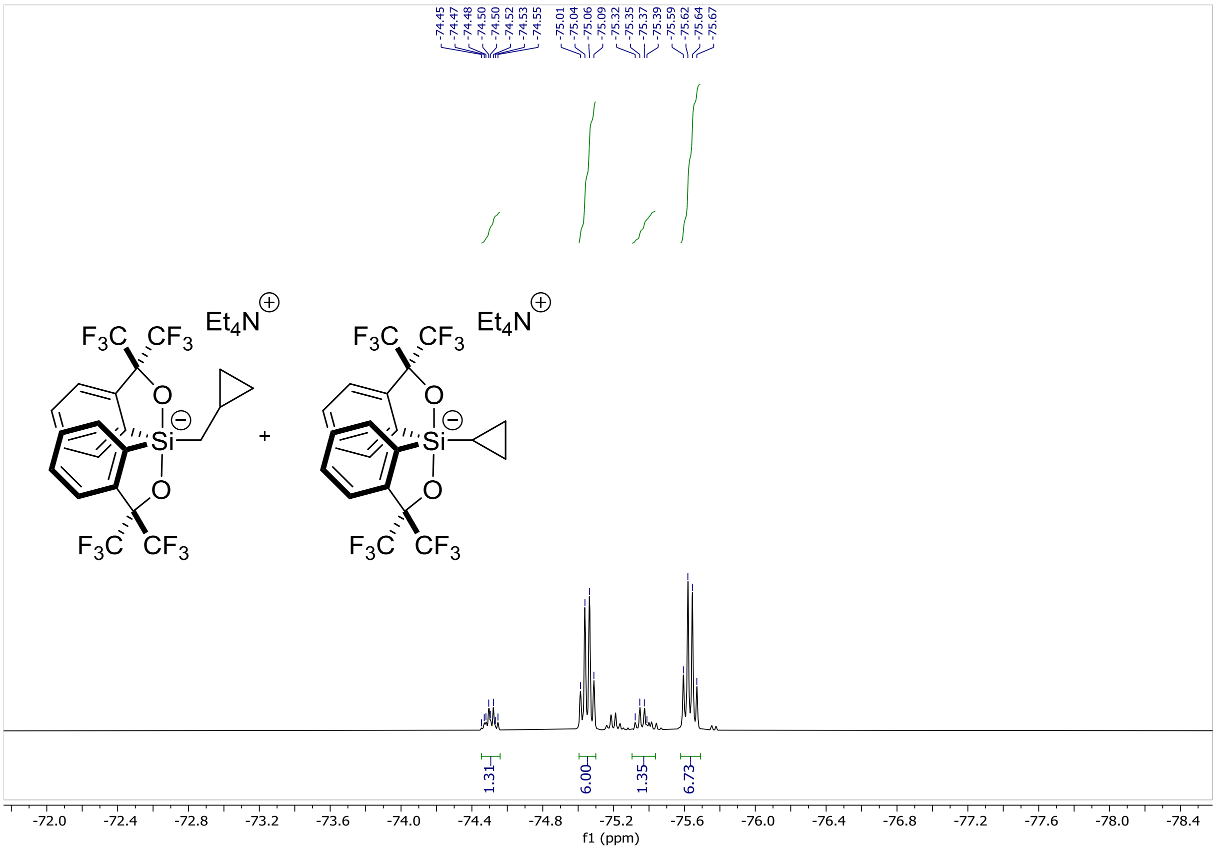
-74.45
-74.47
-74.48
-74.50
-74.50
-74.52
-74.53
-74.55
-75.01
-75.04
-75.06
-75.09
-75.32
-75.35
-75.37
-75.39
-75.59
-75.62
-75.64
-75.67
F3C CF3 Et4N
Si
O
F3C CF3
+
1.31
6.00
1.35
6.73
-72.0 -72.4 -72.8 -73.2 -73.6 -74.0 -74.4 -74.8 -75.2 -75.6 -76.0 -76.4 -76.8 -77.2 -77.6 -78.0 -78.4
f1 (ppm)

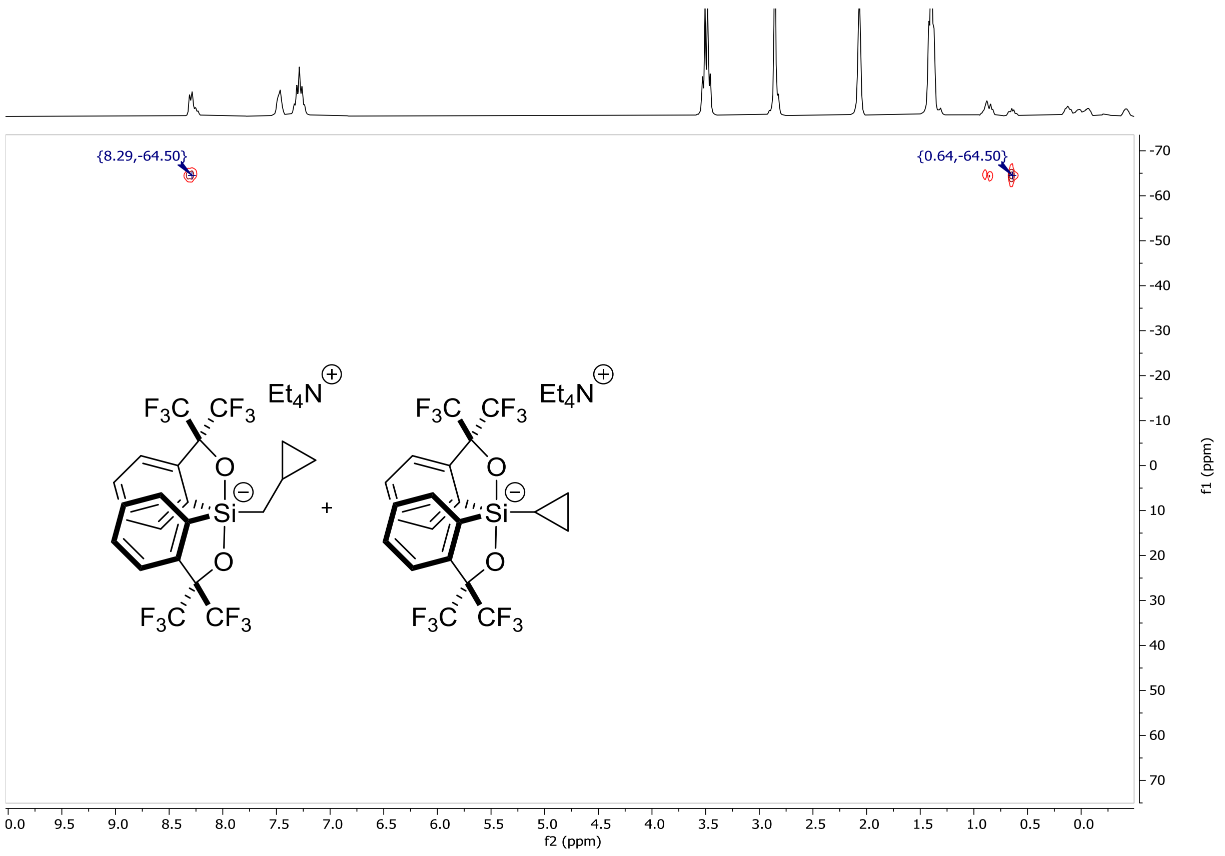

{8.29,-64.50}
{0.64,-64.50}
F3C CF3
Et4N
O
Si
O
+
f1 (ppm)
f2 (ppm)

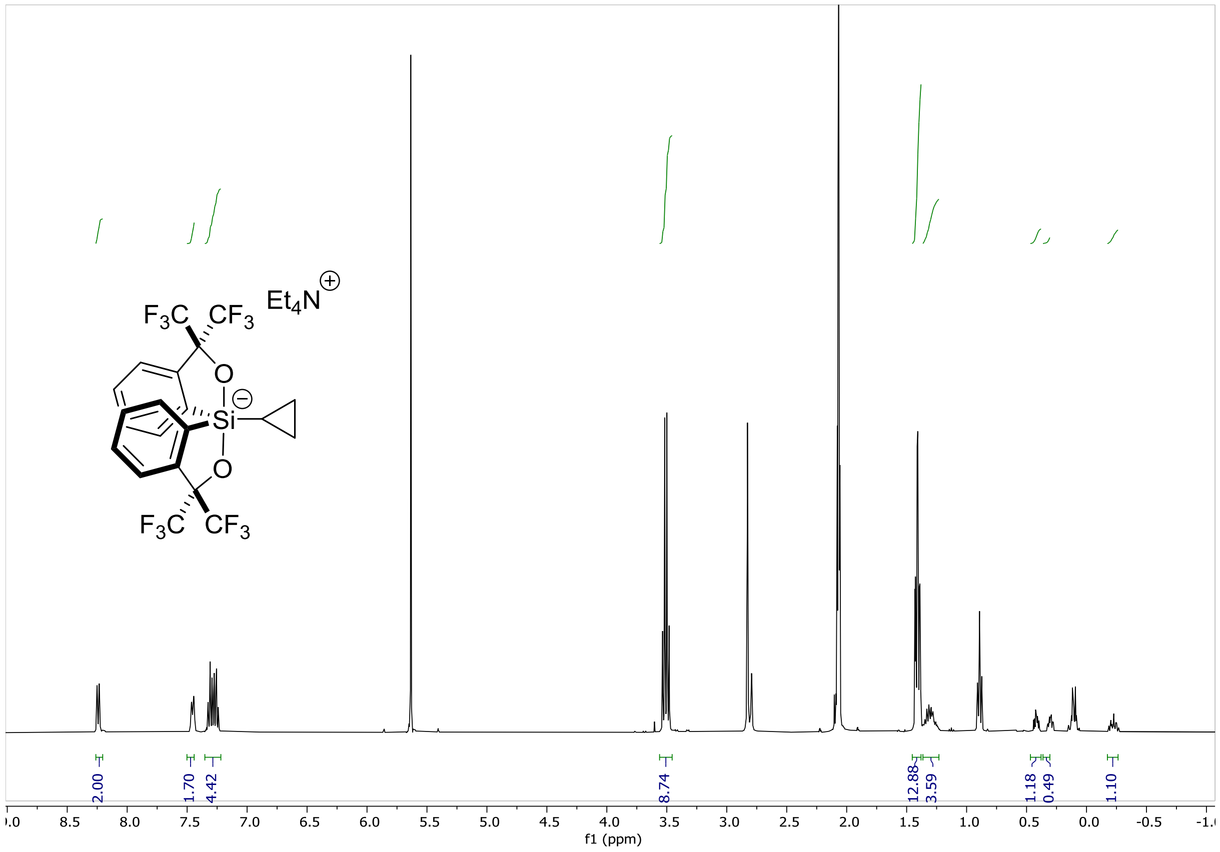

F3C CF3
Et4N
O
Si
O
2.00
1.70
4.42
8.74
12.88
3.59
1.18
0.49
1.10
f1 (ppm)

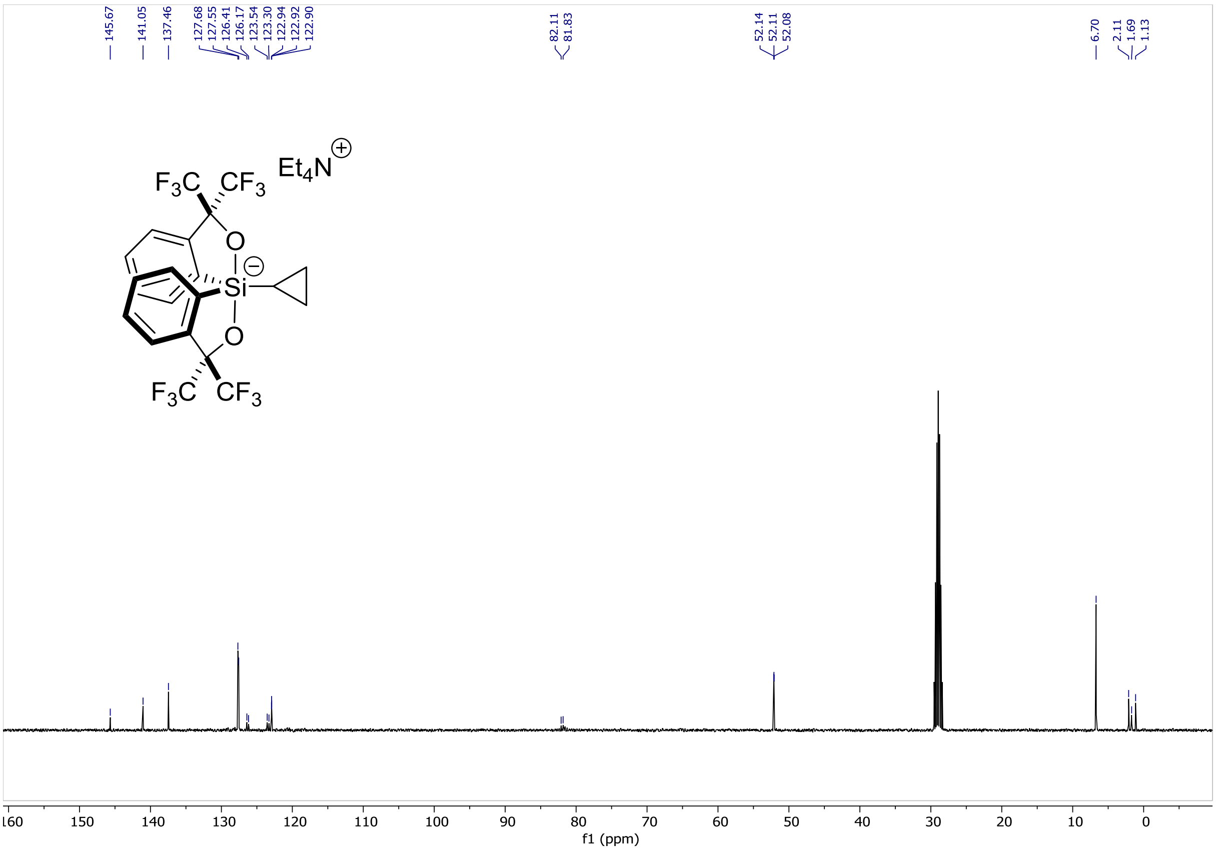
145.67
141.05
137.46
127.68
127.55
126.41
126.17
123.54
123.30
122.94
122.92
122.90
82.11
81.83
52.14
52.11
52.08
6.70
2.11
1.69
1.13
F3C CF3
Et4N
Si
O
F3C CF3
160 150 140 130 120 110 100 90 80 70 60 50 40 30 20 10 0
f1 (ppm)

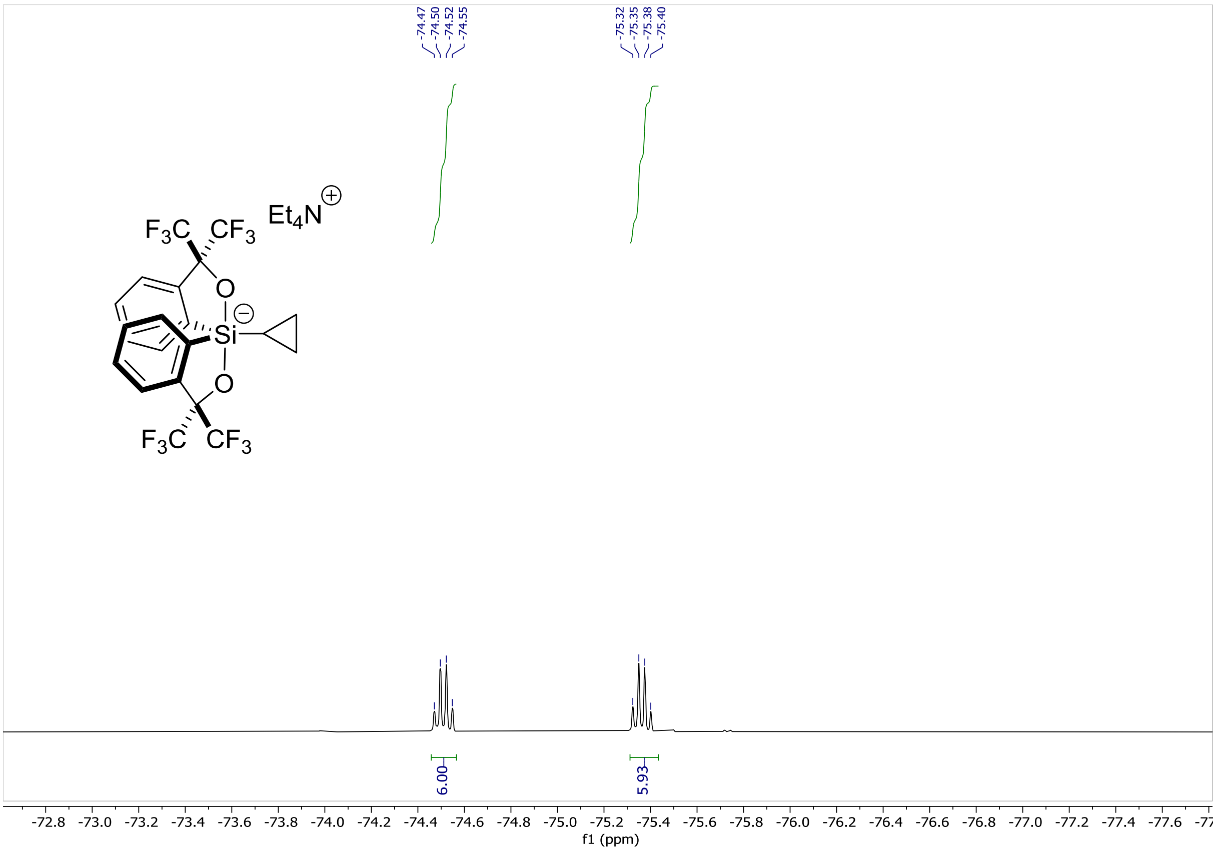
-74.47
-74.50
-74.52
-74.55
-75.32
-75.35
-75.38
-75.40
F3C CF3
Et4N
Si
O
F3C CF3
6.00
5.93
-72.8 -73.0 -73.2 -73.4 -73.6 -73.8 -74.0 -74.2 -74.4 -74.6 -74.8 -75.0 -75.2 -75.4 -75.6 -75.8 -76.0 -76.2 -76.4 -76.6 -76.8 -77.0 -77.2 -77.4 -77.6
f1 (ppm)

^{1}H, ^{13}C, ^{19}F, and $^{1}H/^{29}Si$ HMQC NMR spectra of 3f in $(CD_3)_2CO$

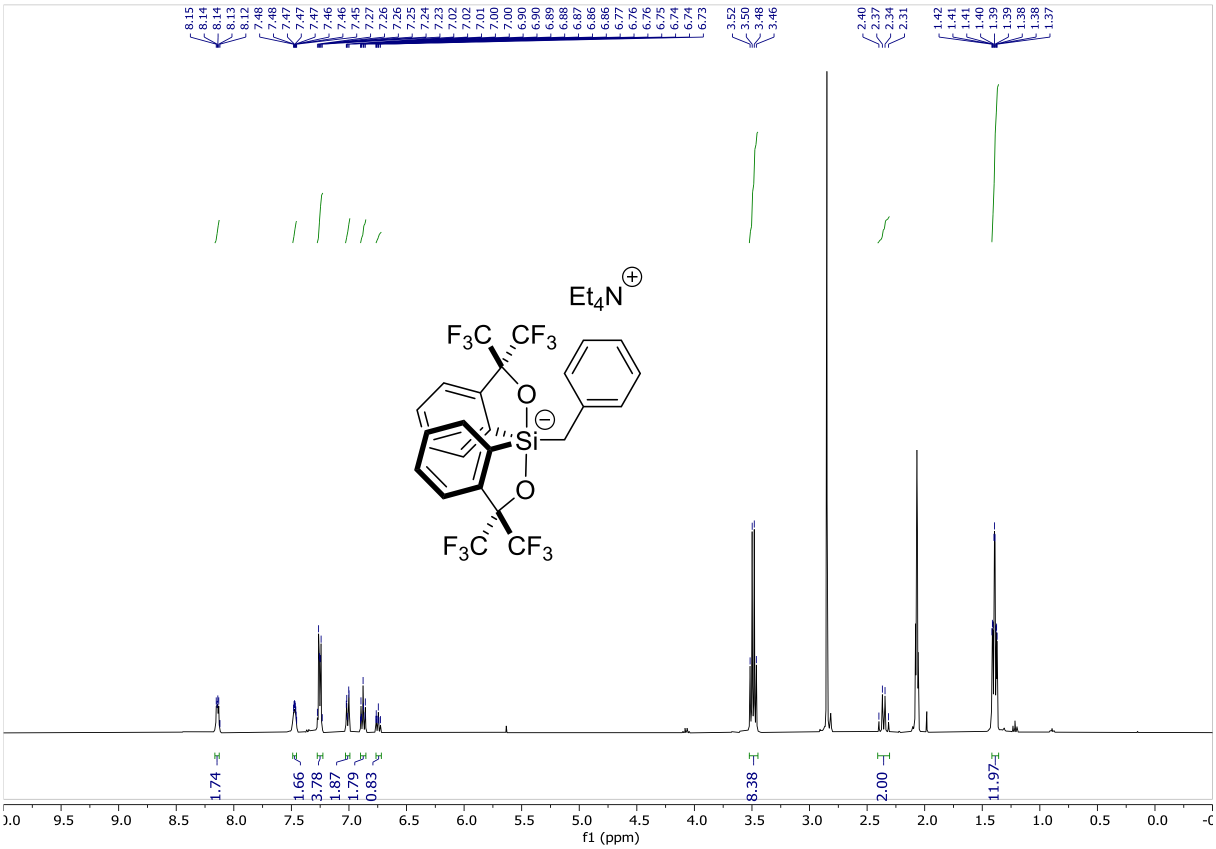

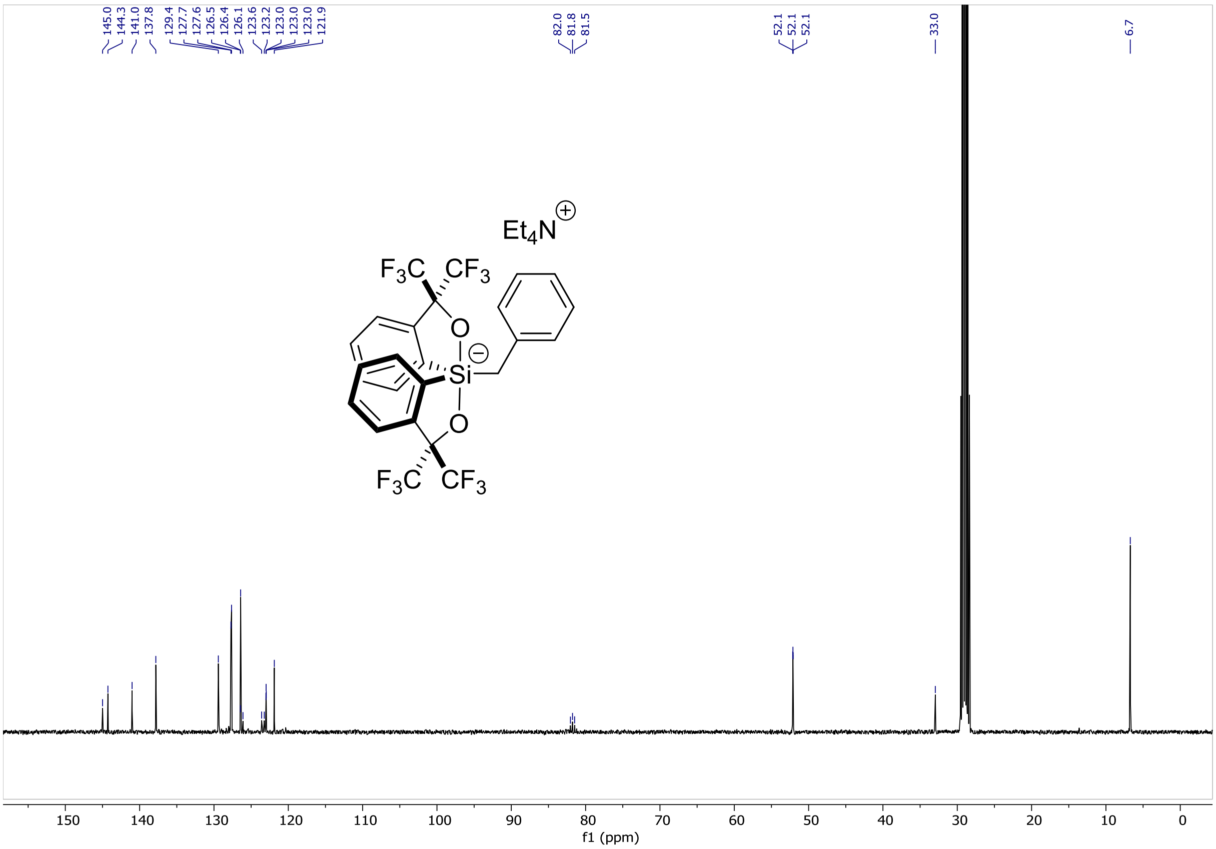
145.0
144.3
141.0
137.8
129.4
127.7
127.6
126.5
126.4
126.1
123.6
123.2
123.0
123.0
123.0
121.9
82.0
81.8
81.5
52.1
52.1
52.1
33.0
6.7
Et4N
F3C
CF3
O
Si
O
F3C
CF3
f1 (ppm)

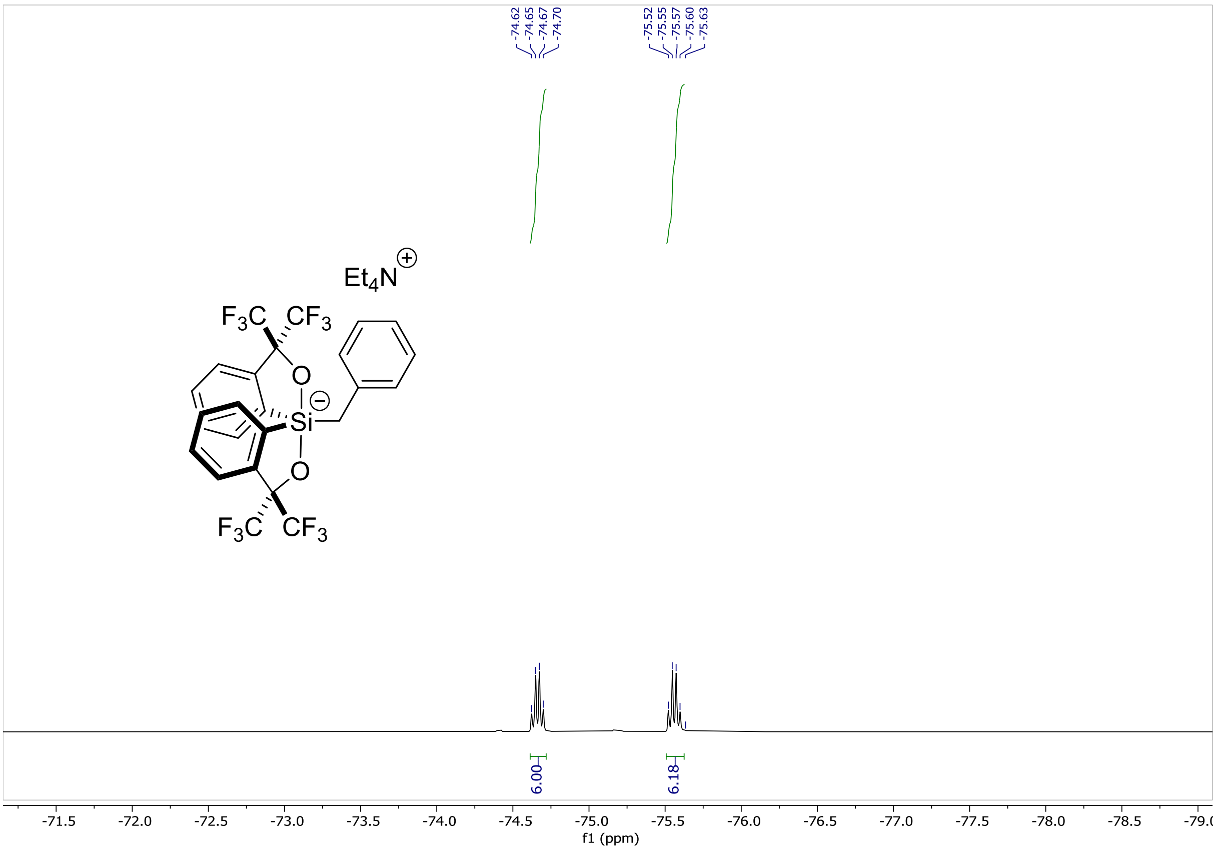
-74.62
-74.65
-74.67
-74.70
-75.52
-75.55
-75.57
-75.60
-75.63
Et4N
F3C
CF3
O
Si
O
F3C
CF3
6.00
6.18
f1 (ppm)

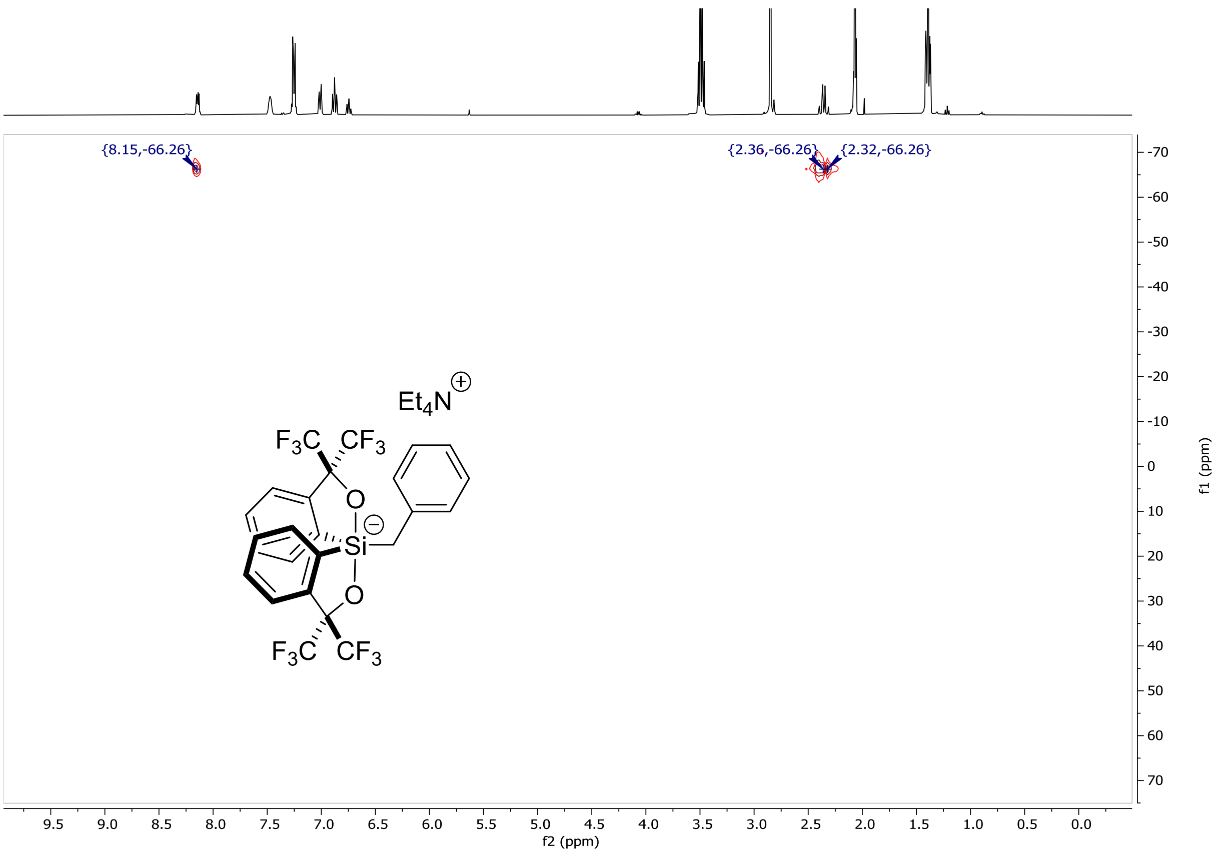

^{1}H, ^{13}C, ^{19}F, and $^{1}H/^{29}Si$ HMQC NMR spectra of 3g in $(CD_3)_2CO$

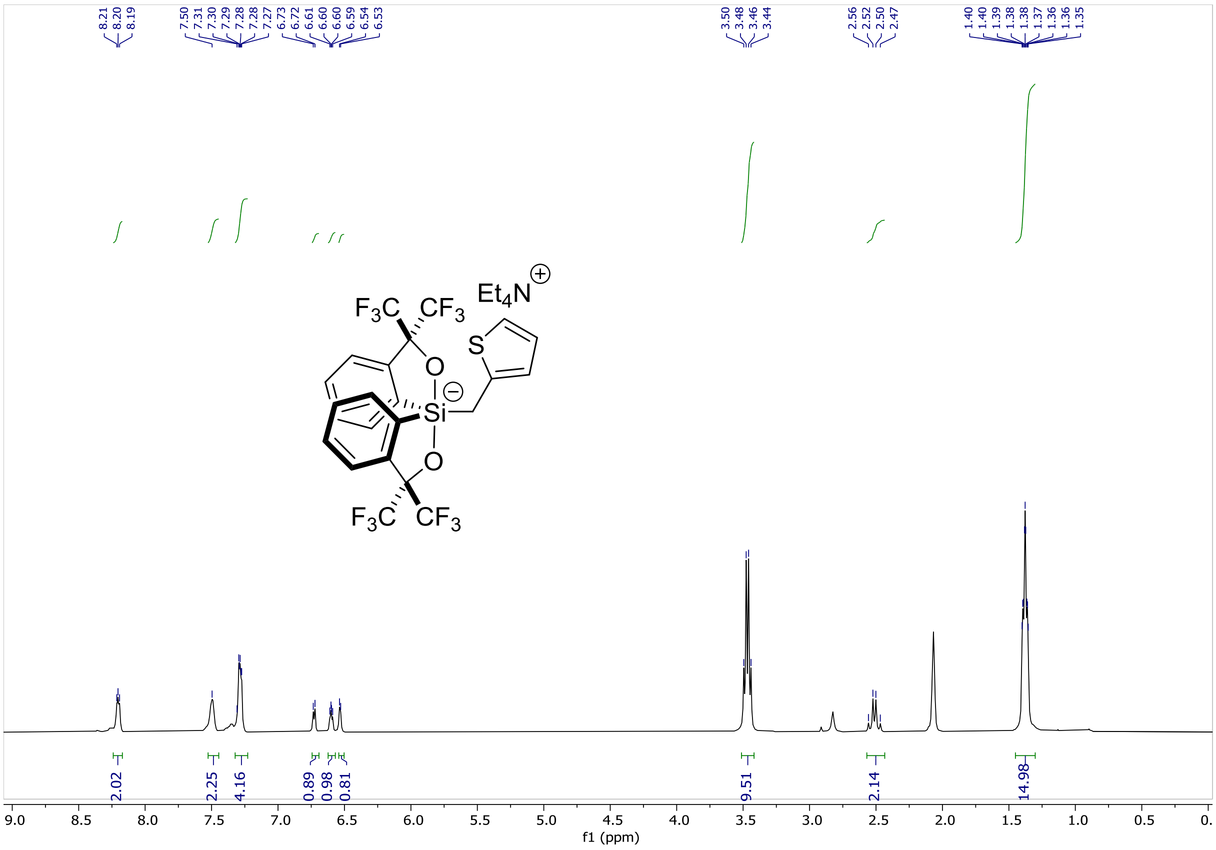
8.21
8.20
8.19
7.50
7.31
7.30
7.29
7.28
7.28
7.27
6.73
6.72
6.61
6.60
6.60
6.59
6.54
6.53
3.50
3.48
3.46
3.44
2.56
2.52
2.50
2.47
1.40
1.40
1.39
1.38
1.38
1.37
1.36
1.36
1.35
Et4N
F3C
CF3
S
O
Si
O
F3C
CF3
2.02
2.25
4.16
0.89
0.98
0.81
9.51
2.14
14.98
9.0 8.5 8.0 7.5 7.0 6.5 6.0 5.5 5.0 4.5 4.0 3.5 3.0 2.5 2.0 1.5 1.0 0.5 0.
f1 (ppm)

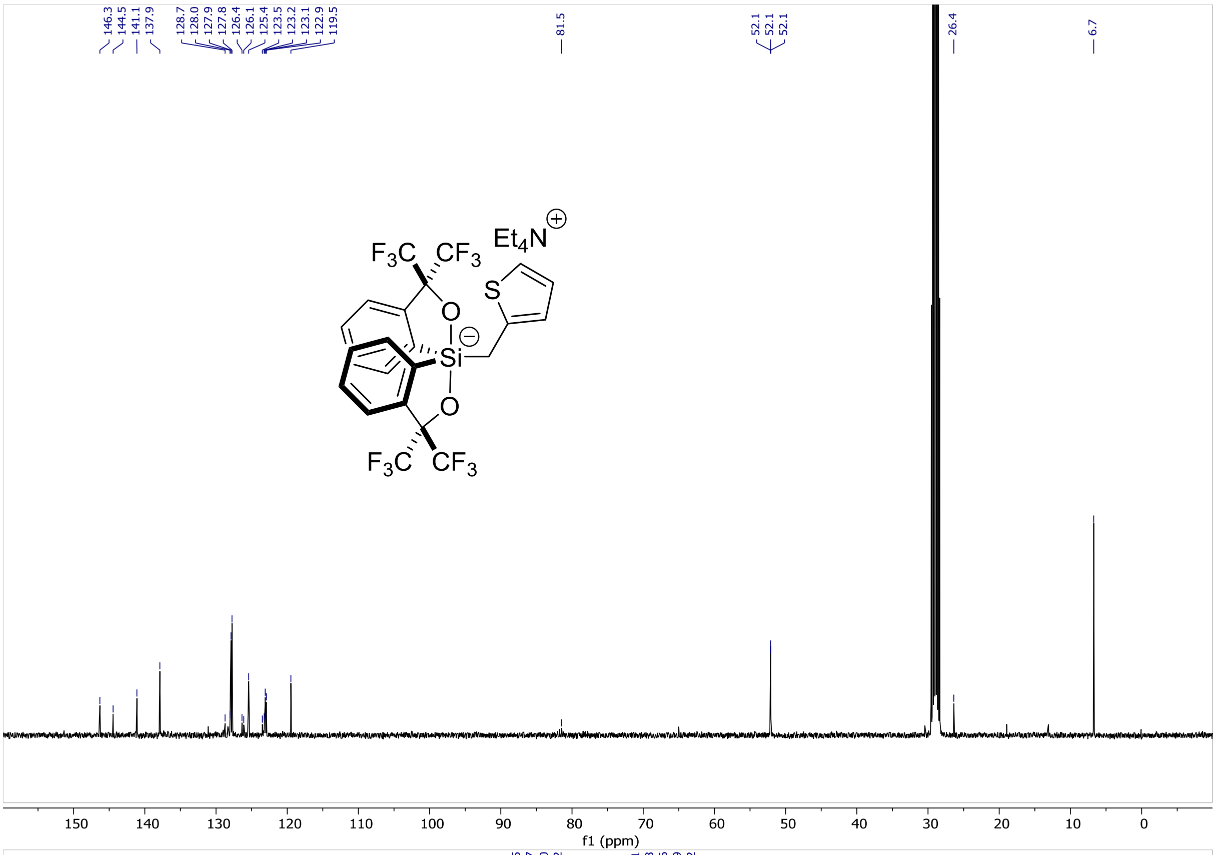
146.3
144.5
141.1
137.9
128.7
128.0
127.9
127.8
126.4
126.1
125.4
123.5
123.2
123.1
122.9
119.5
81.5
52.1
52.1
52.1
26.4
6.7
F_3C CF_3 $Et_4N^{\oplus}$
S
O
Si
O
F_3C CF_3
150 140 130 120 110 100 90 80 70 60 50 40 30 20 10 0
f1 (ppm)

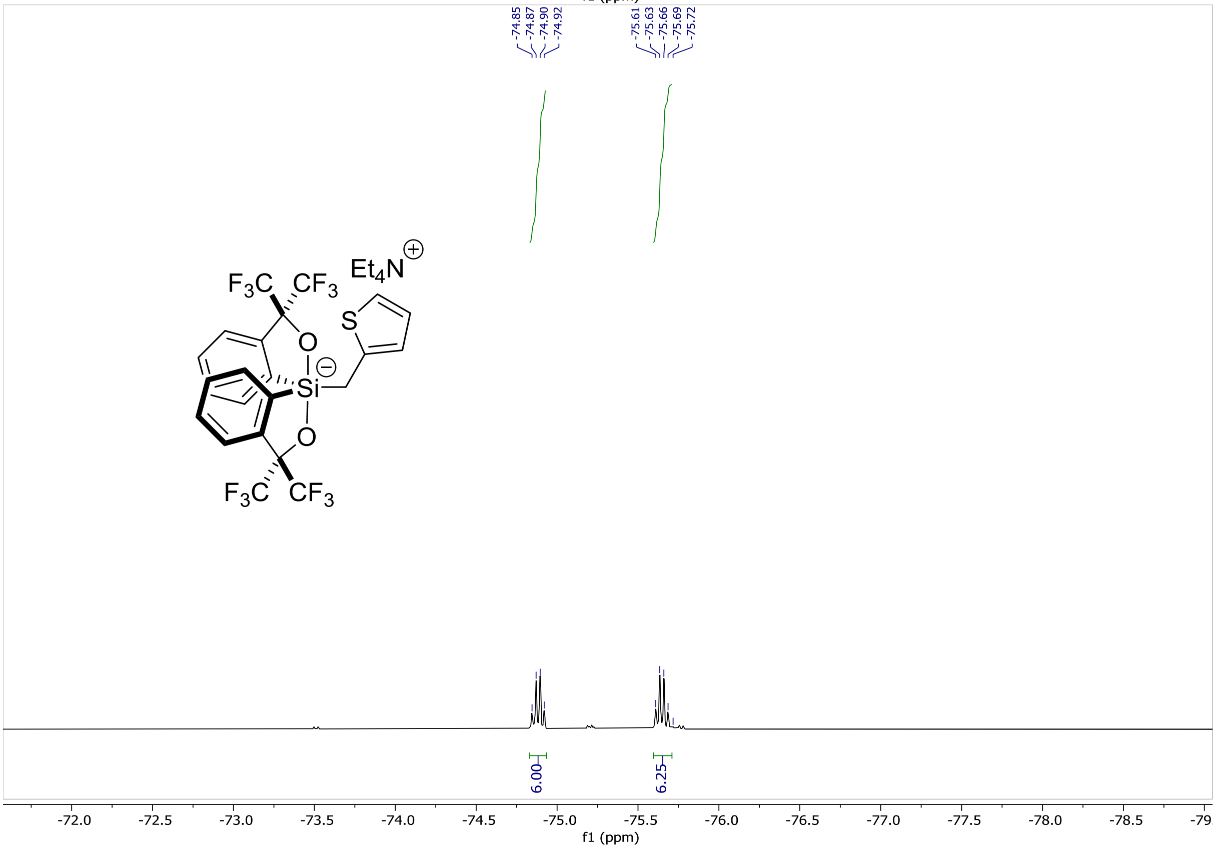
-74.85
-74.87
-74.90
-74.92
-75.61
-75.63
-75.66
-75.69
-75.72
F_3C CF_3 $Et_4N^{\oplus}$
S
O
Si
O
F_3C CF_3
6.00
6.25
-72.0 -72.5 -73.0 -73.5 -74.0 -74.5 -75.0 -75.5 -76.0 -76.5 -77.0 -77.5 -78.0 -78.5 -79
f1 (ppm)

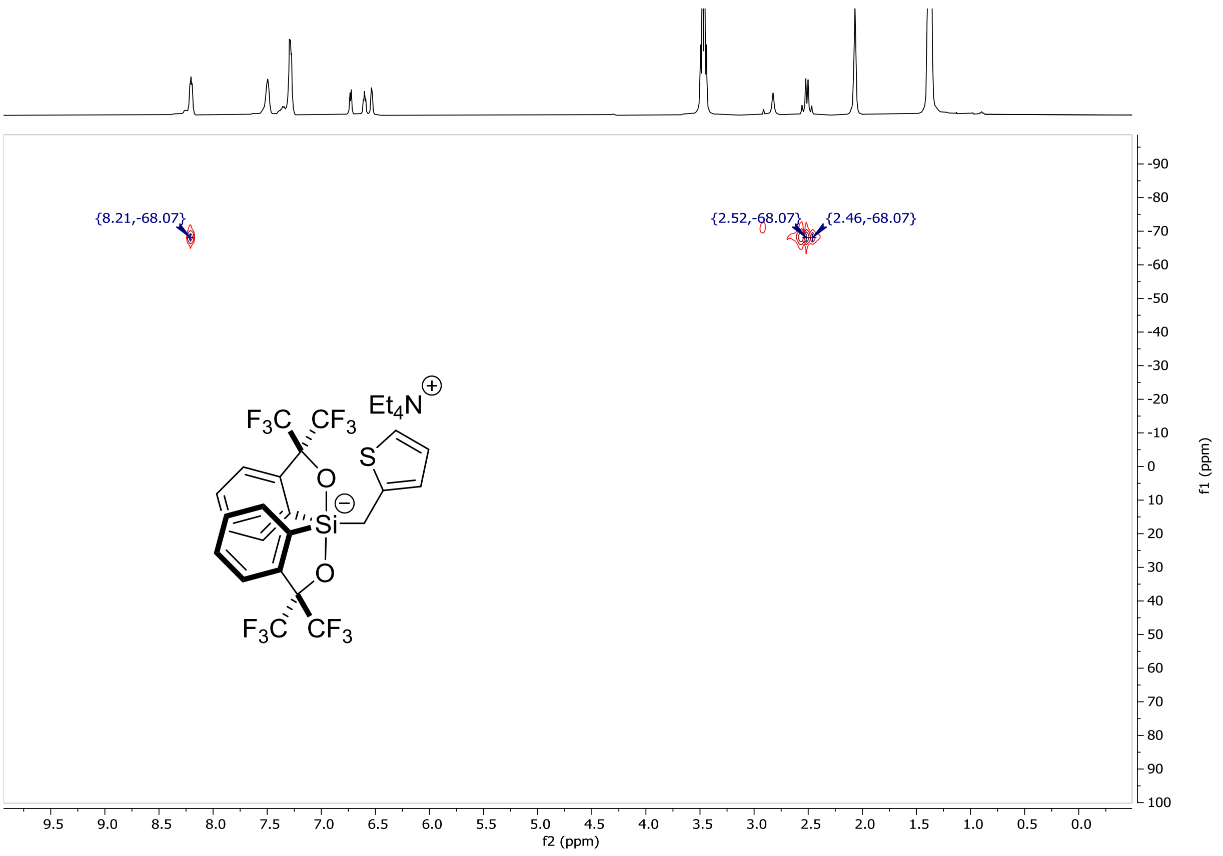

^{1}H, ^{13}C, ^{19}F, and $^{1}H/^{29}Si$ HMQC NMR spectra of 5 in $CDCl_3$

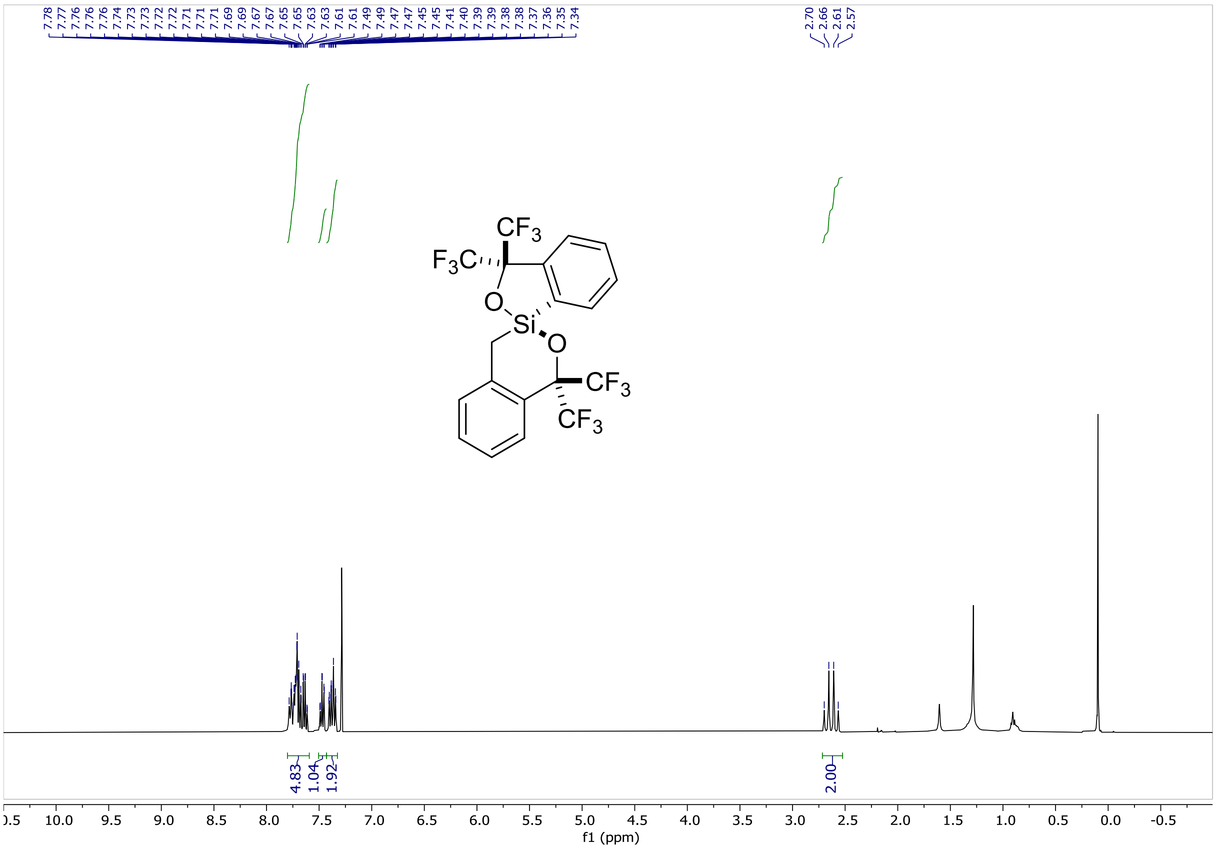
7.78 7.77 7.76 7.76 7.74 7.73 7.73 7.72 7.72 7.71 7.71 7.71 7.69 7.69 7.67 7.65 7.65 7.63 7.63 7.61 7.61 7.49 7.49 7.47 7.45 7.45 7.41 7.40 7.39 7.39 7.38 7.38 7.37 7.36 7.35 7.34
2.70 2.66 2.61 2.57
CF3
F3C
O
Si
O
CF3
CF3
4.83
1.04
1.92
2.00
0.5 10.0 9.5 9.0 8.5 8.0 7.5 7.0 6.5 6.0 5.5 5.0 4.5 4.0 3.5 3.0 2.5 2.0 1.5 1.0 0.5 0.0 -0.5
f1 (ppm)

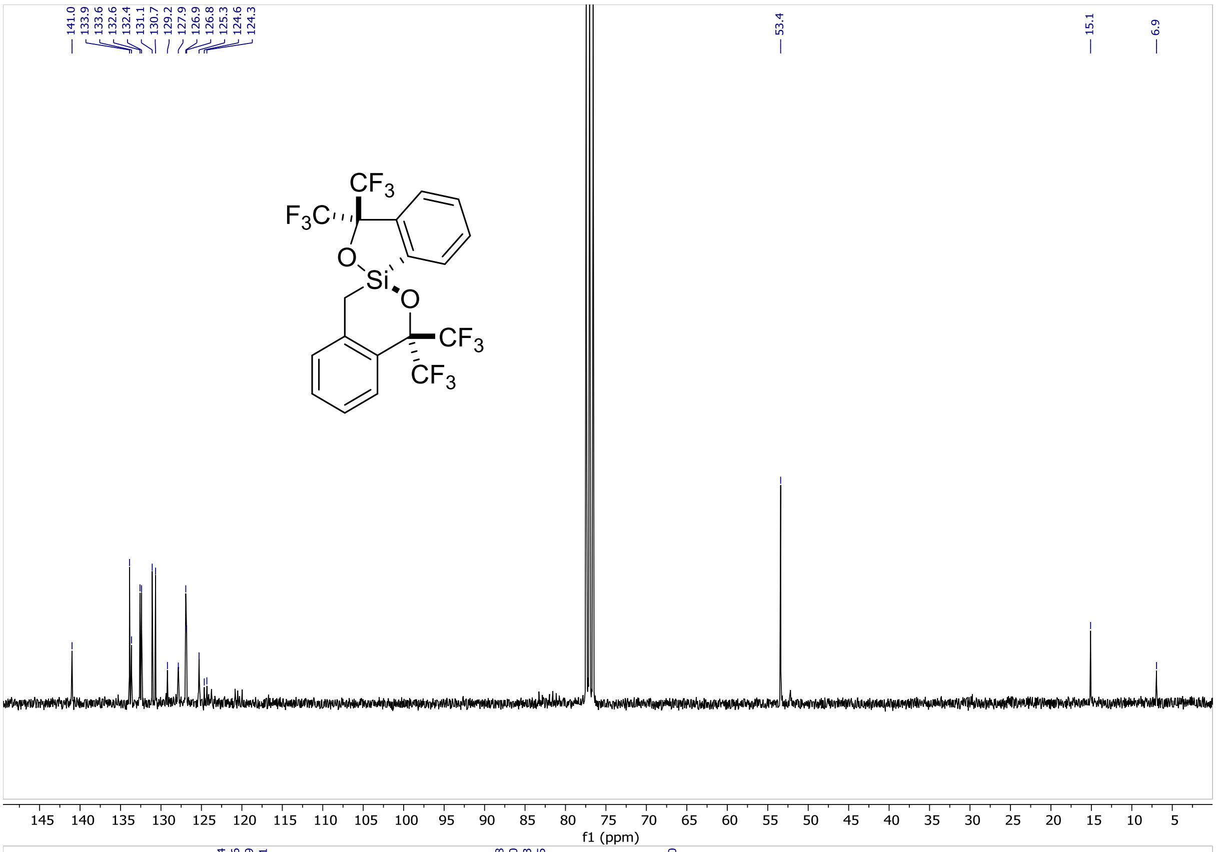
141.0
133.9
133.6
132.6
132.4
131.1
130.7
129.2
127.9
126.9
126.8
125.3
124.6
124.3
53.4
15.1
6.9
CF_3
F_3C
O
Si
O
CF_3
CF_3
145 140 135 130 125 120 115 110 105 100 95 90 85 80 75 70 65 60 55 50 45 40 35 30 25 20 15 10 5
f1 (ppm)

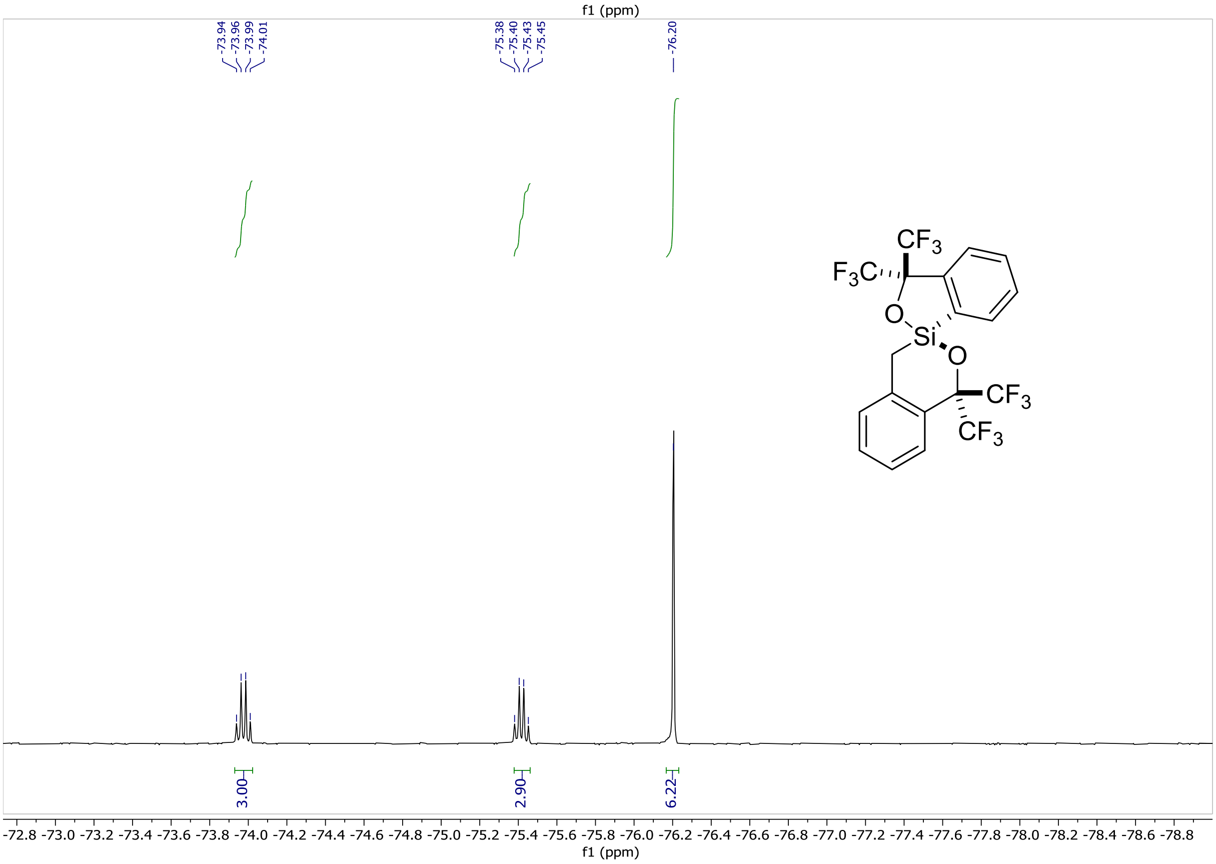
-73.94
-73.96
-73.99
-74.01
-75.38
-75.40
-75.43
-75.45
-76.20
CF_3
F_3C
O
Si
O
CF_3
CF_3
3.00
2.90
6.22
-72.8 -73.0 -73.2 -73.4 -73.6 -73.8 -74.0 -74.2 -74.4 -74.6 -74.8 -75.0 -75.2 -75.4 -75.6 -75.8 -76.0 -76.2 -76.4 -76.6 -76.8 -77.0 -77.2 -77.4 -77.6 -77.8 -78.0 -78.2 -78.4 -78.6 -78.8
f1 (ppm)

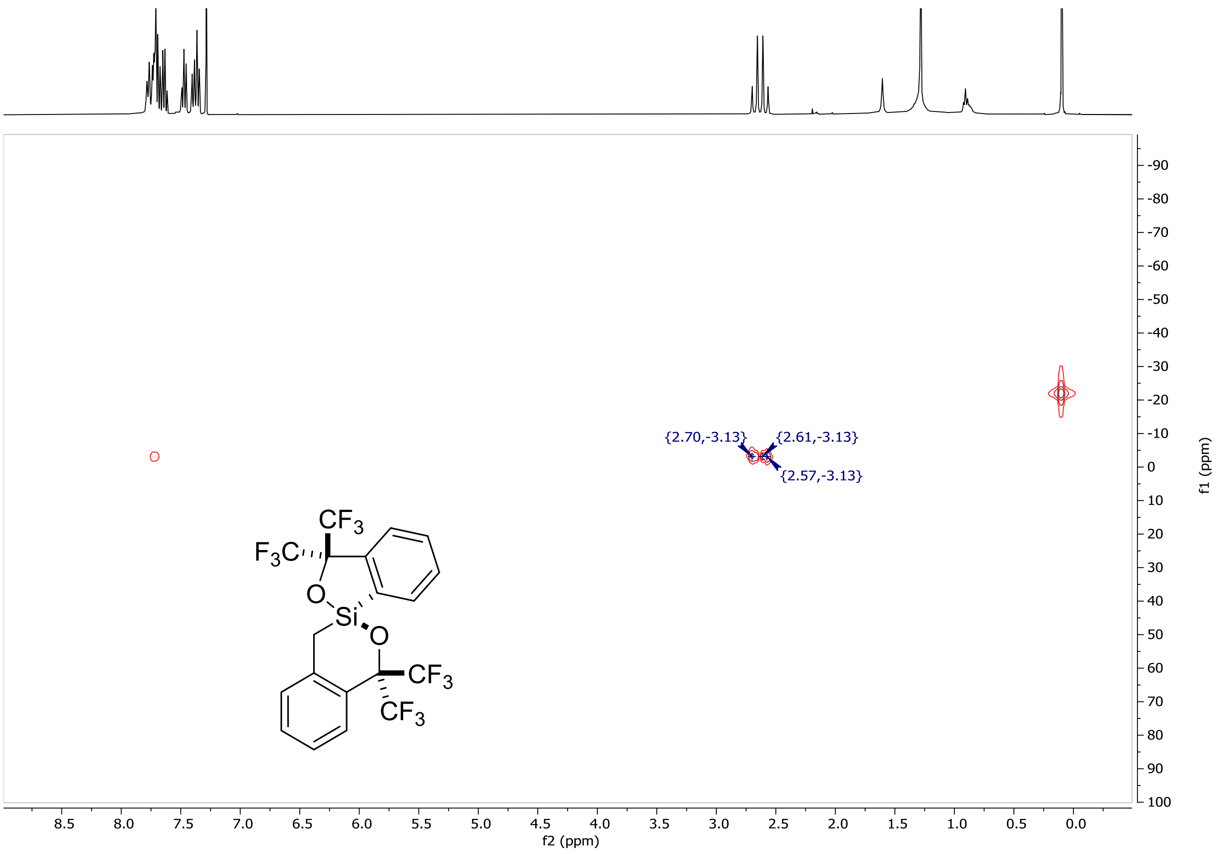

^{1}H, ^{13}C, ^{19}F, and $^{1}H/^{29}Si$ HMQC NMR spectra of 7 in CD_3CN

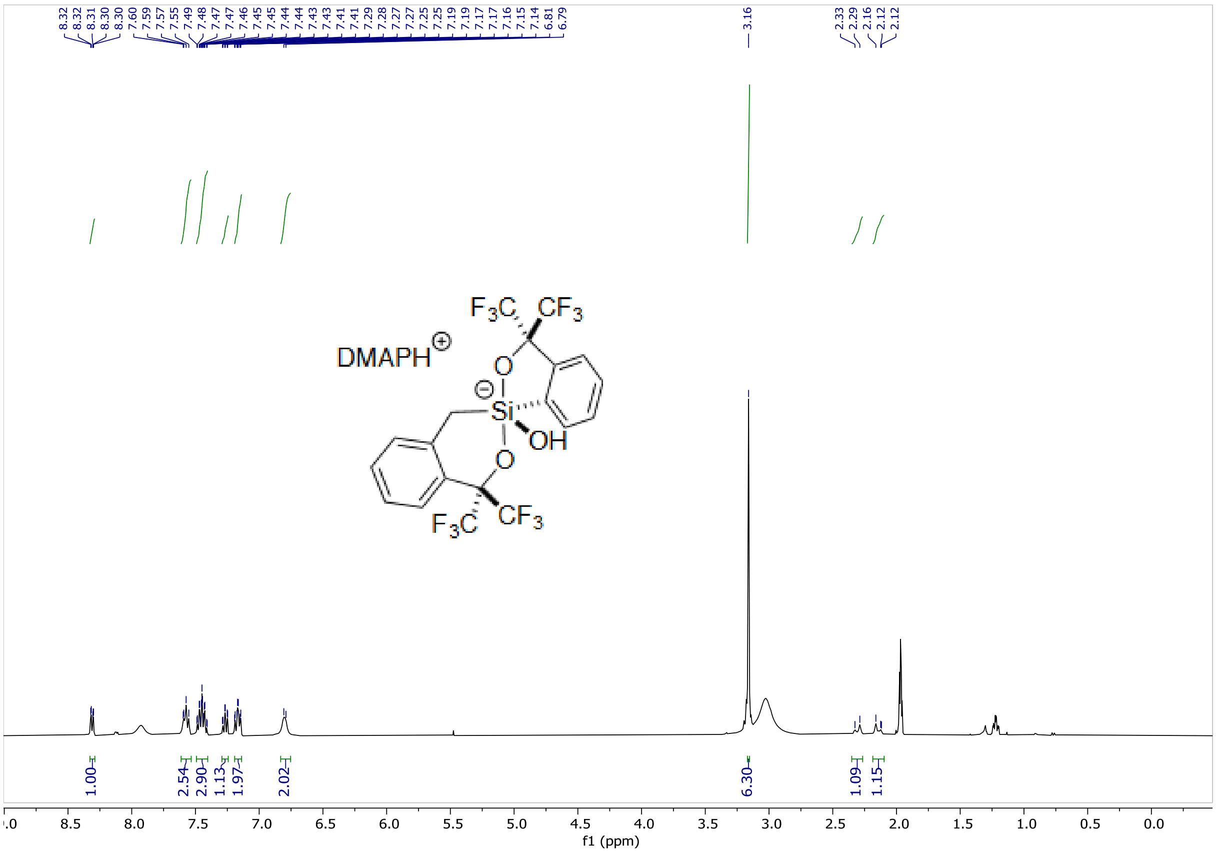
DMAPH
f1 (ppm)

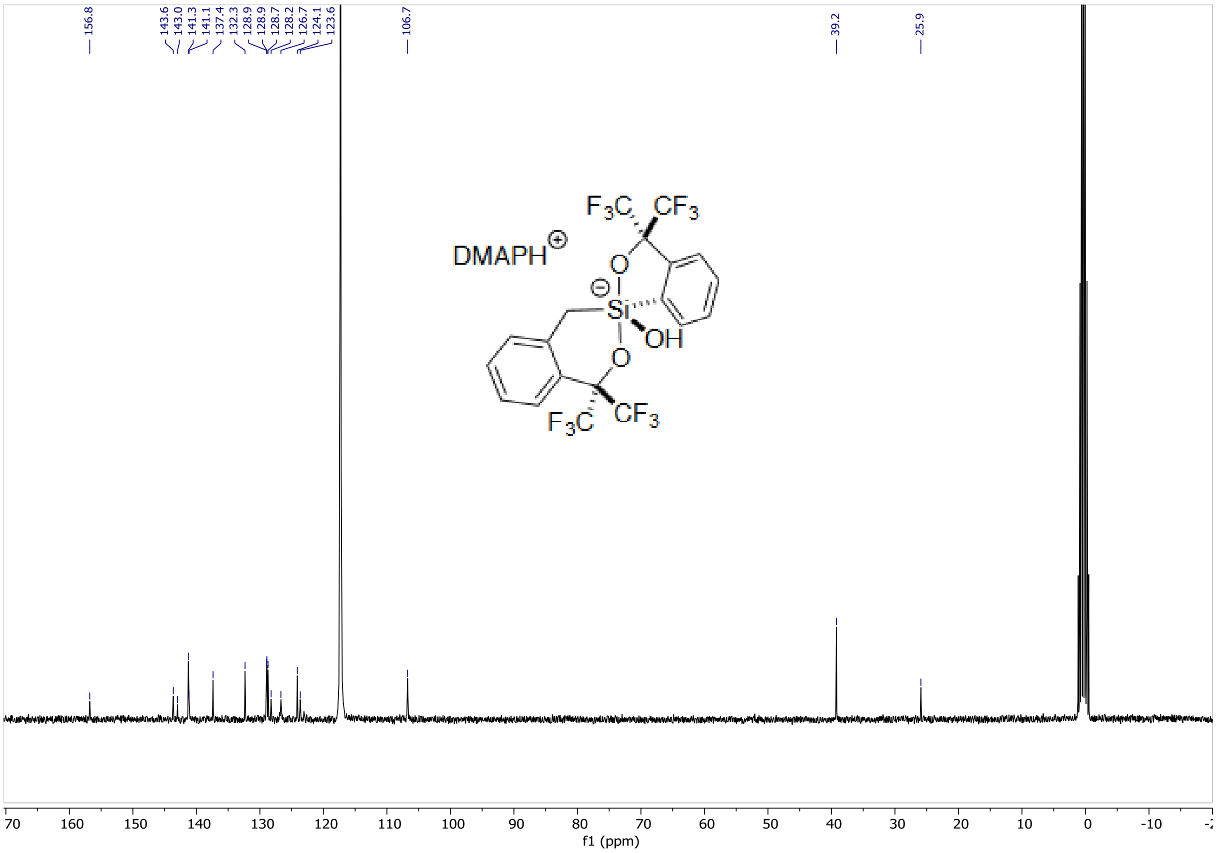
DMAPH
f1 (ppm)

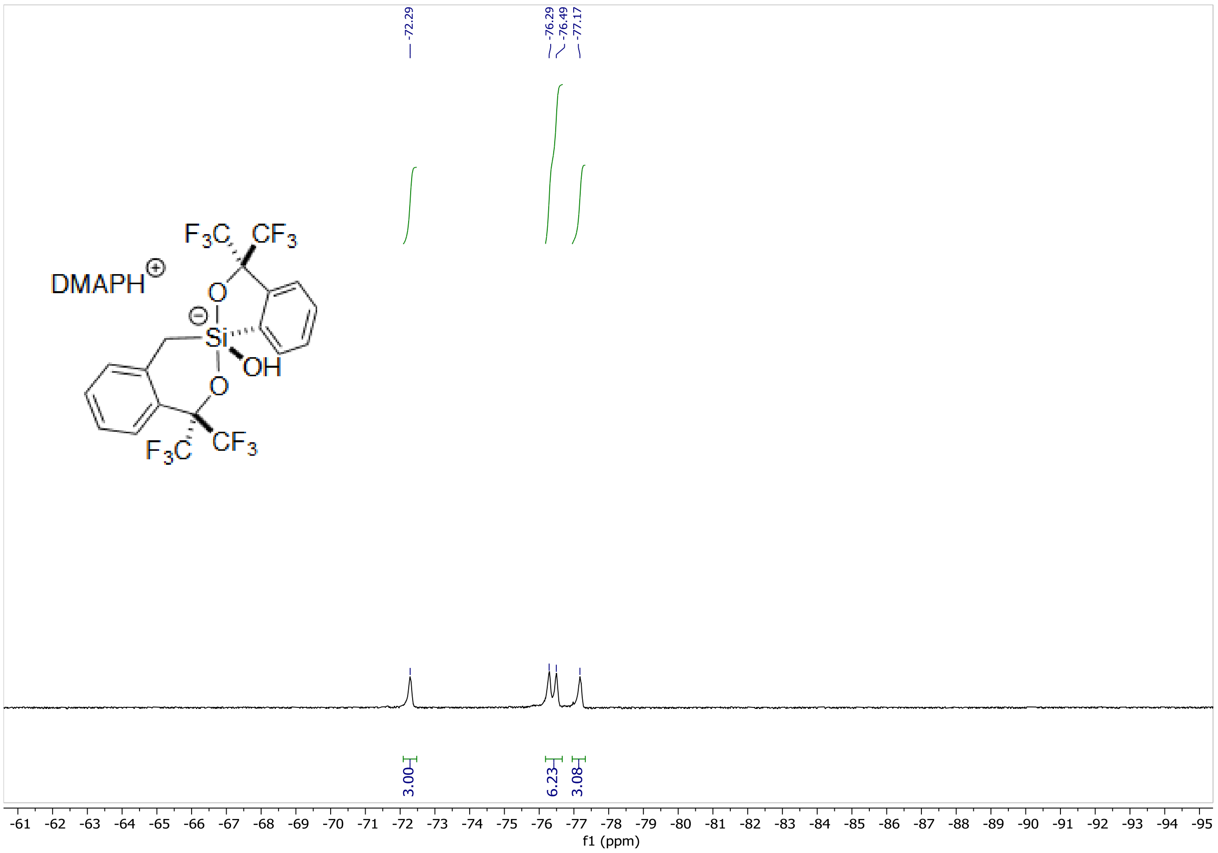
-72.29
-76.29
-76.49
-77.17
DMAPH⊕
F3C CF3
O
⊖
Si
OH
O
F3C CF3
3.00
6.23
3.08
-61 -62 -63 -64 -65 -66 -67 -68 -69 -70 -71 -72 -73 -74 -75 -76 -77 -78 -79 -80 -81 -82 -83 -84 -85 -86 -87 -88 -89 -90 -91 -92 -93 -94 -95
f1 (ppm)

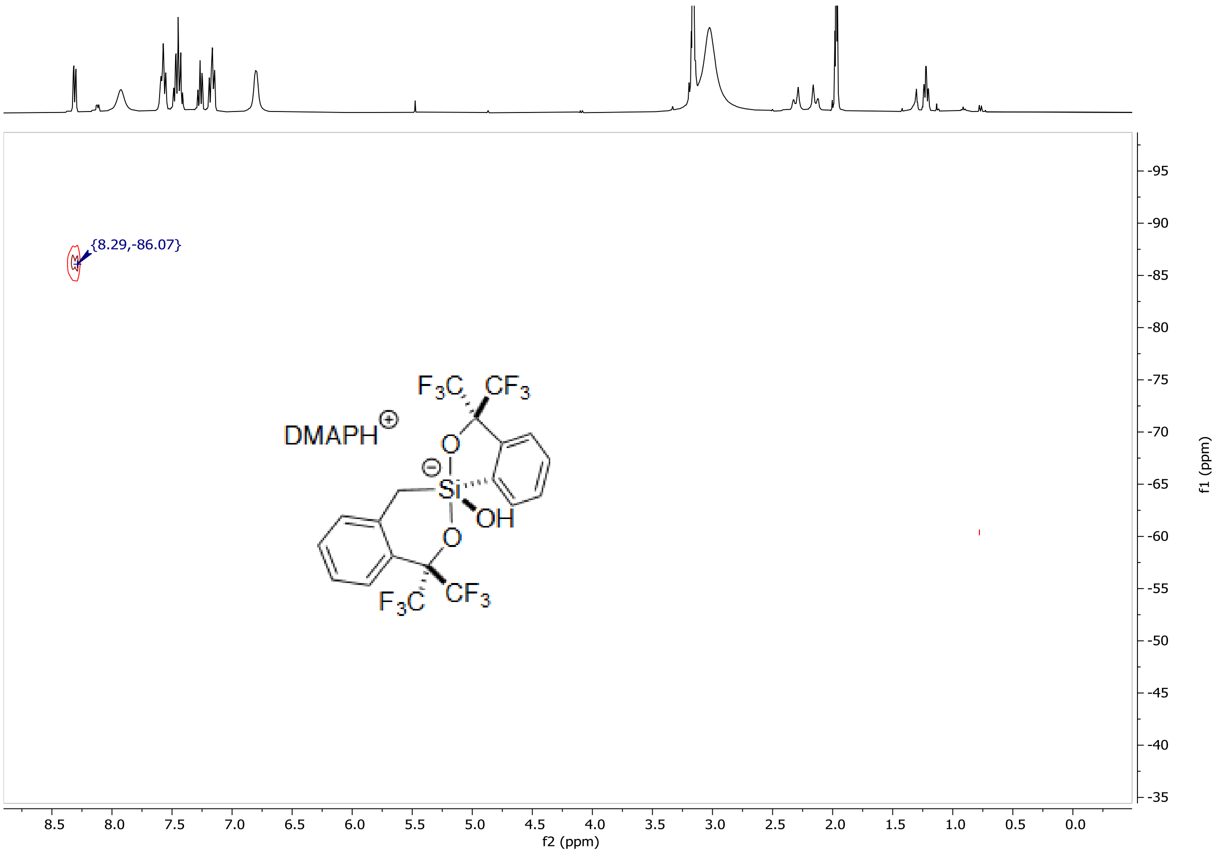
{8.29,-86.07}
DMAPH⊕
F3C CF3
O
⊖
Si
OH
O
F3C CF3
8.5 8.0 7.5 7.0 6.5 6.0 5.5 5.0 4.5 4.0 3.5 3.0 2.5 2.0 1.5 1.0 0.5 0.0
f2 (ppm)
-95 -90 -85 -80 -75 -70 -65 -60 -55 -50 -45 -40 -35
f1 (ppm)

^1H, ^{13}C, ^{19}F, and $^1H/^{29}Si$ HMQC NMR spectra of 7-Et_4N^+ in $(CD_3)_2CO$

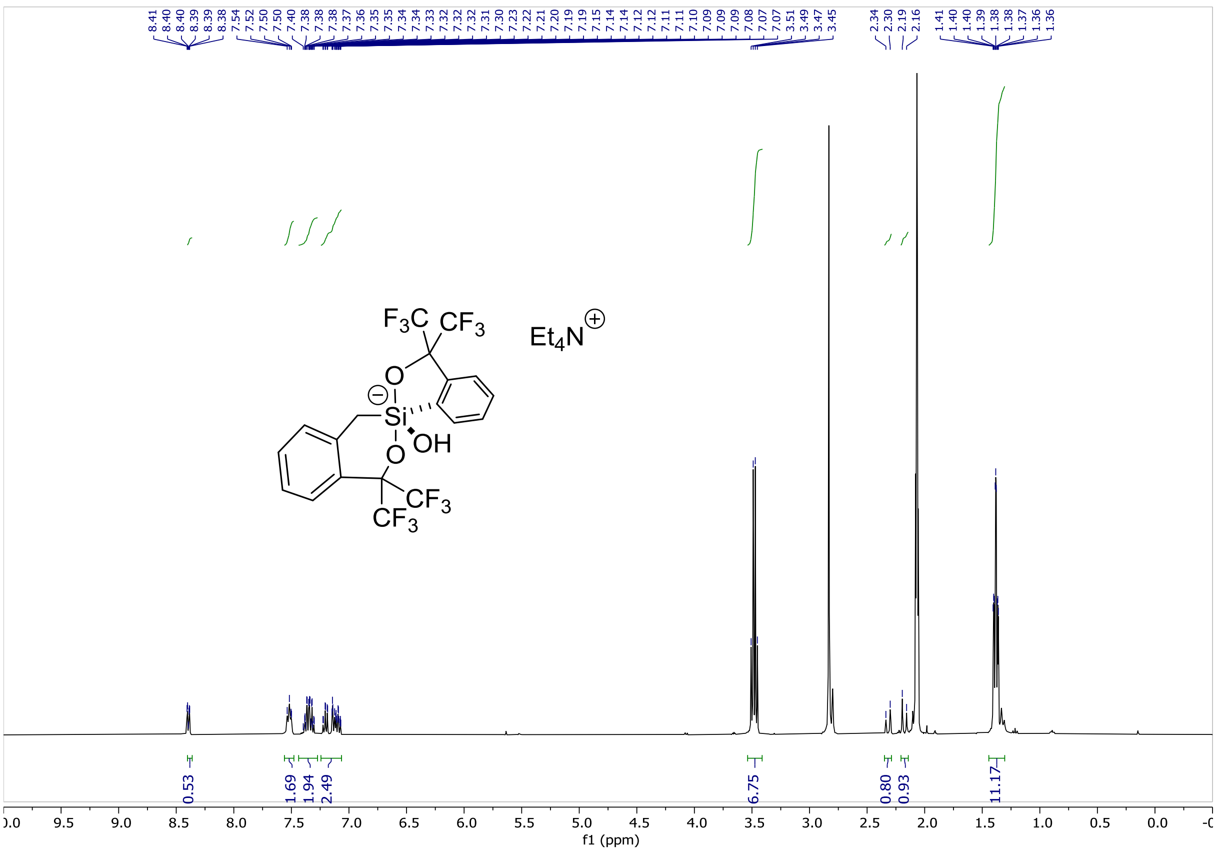

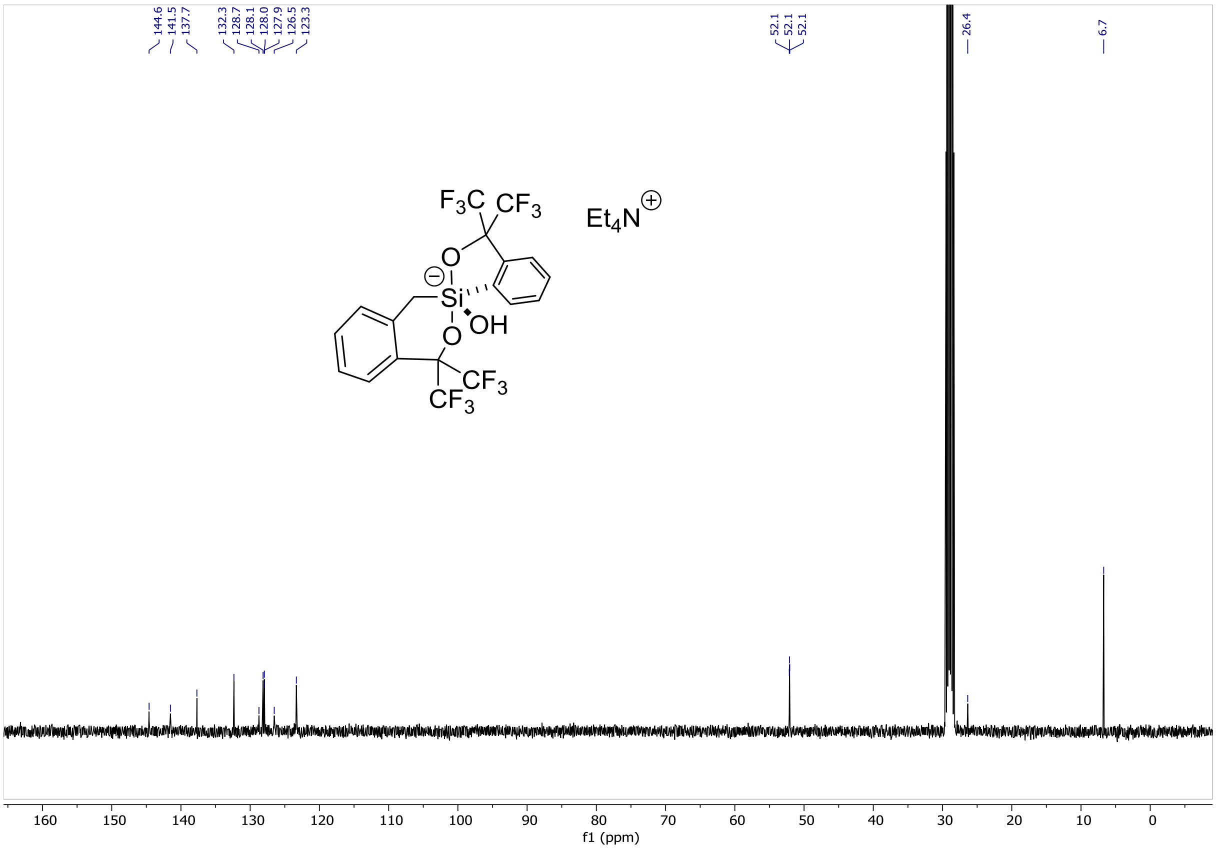
144.6
141.5
137.7
132.3
128.7
128.1
128.0
127.9
126.5
123.3
52.1
52.1
52.1
26.4
6.7
F3C CF3
Et4N
O
Si
OH
O
CF3
CF3
f1 (ppm)

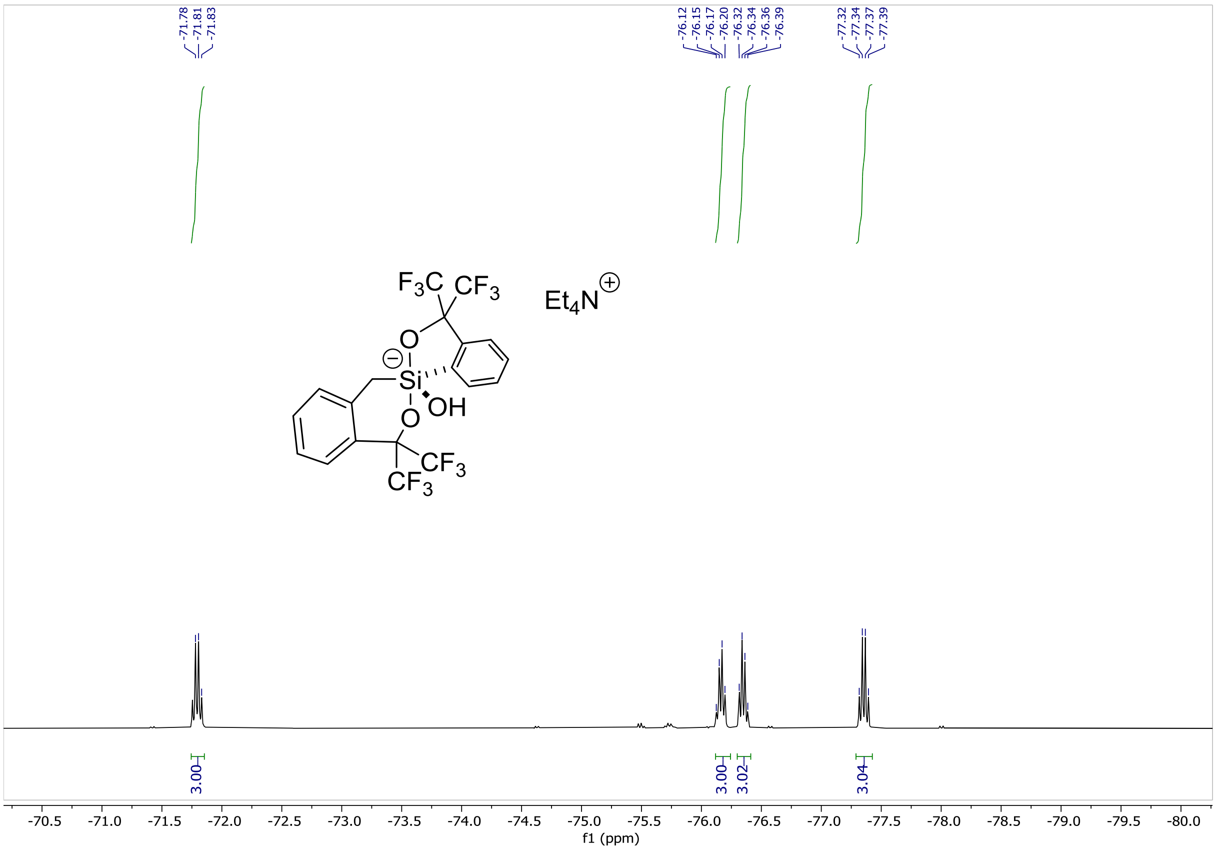
-71.78
-71.81
-71.83
-76.12
-76.15
-76.17
-76.20
-76.32
-76.34
-76.36
-76.39
-77.32
-77.34
-77.37
-77.39
F3C CF3
Et4N
O
Si
OH
O
CF3
CF3
3.00
3.00
3.02
3.04
f1 (ppm)

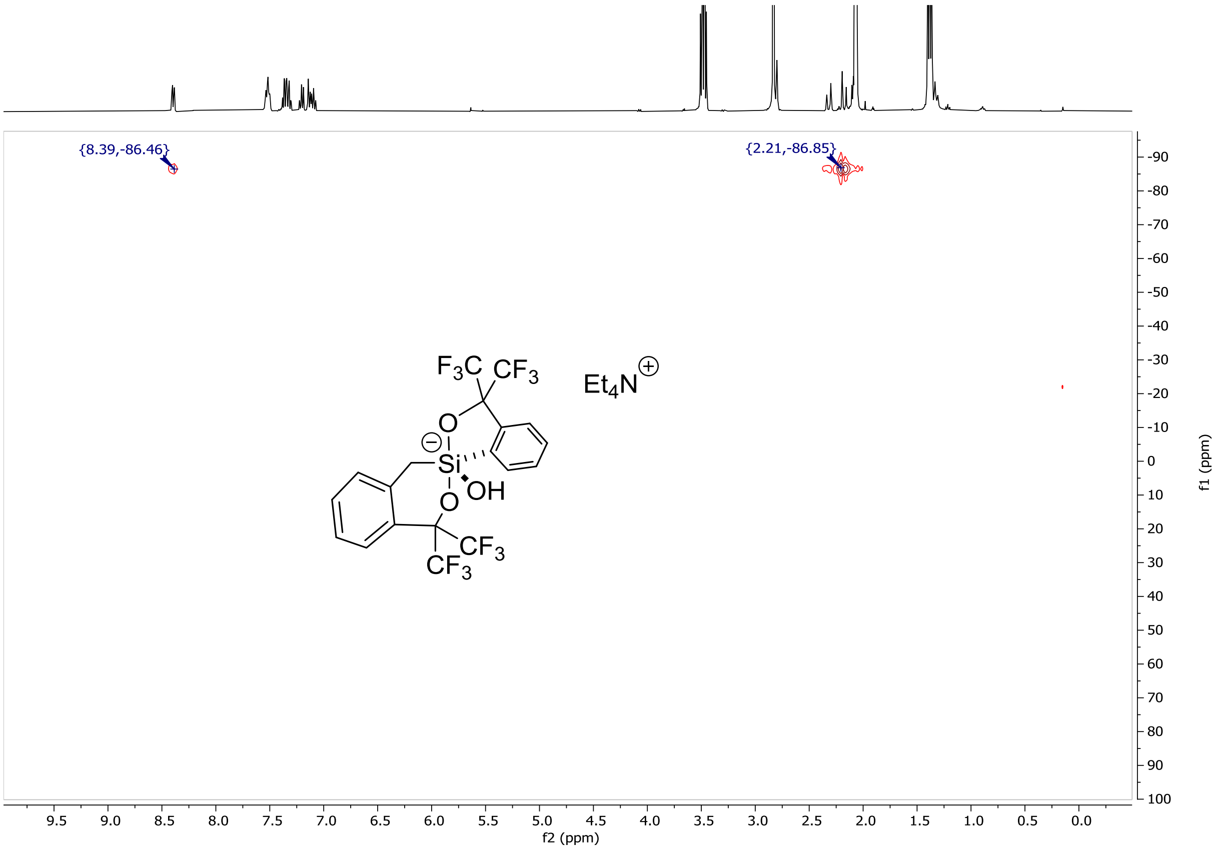

^{1}H, ^{13}C, ^{19}F, NMR spectra of 8 in $CDCl_3$ and ^{1}H/^{29}Si HMQC spectrum of 8 in CD_3CN

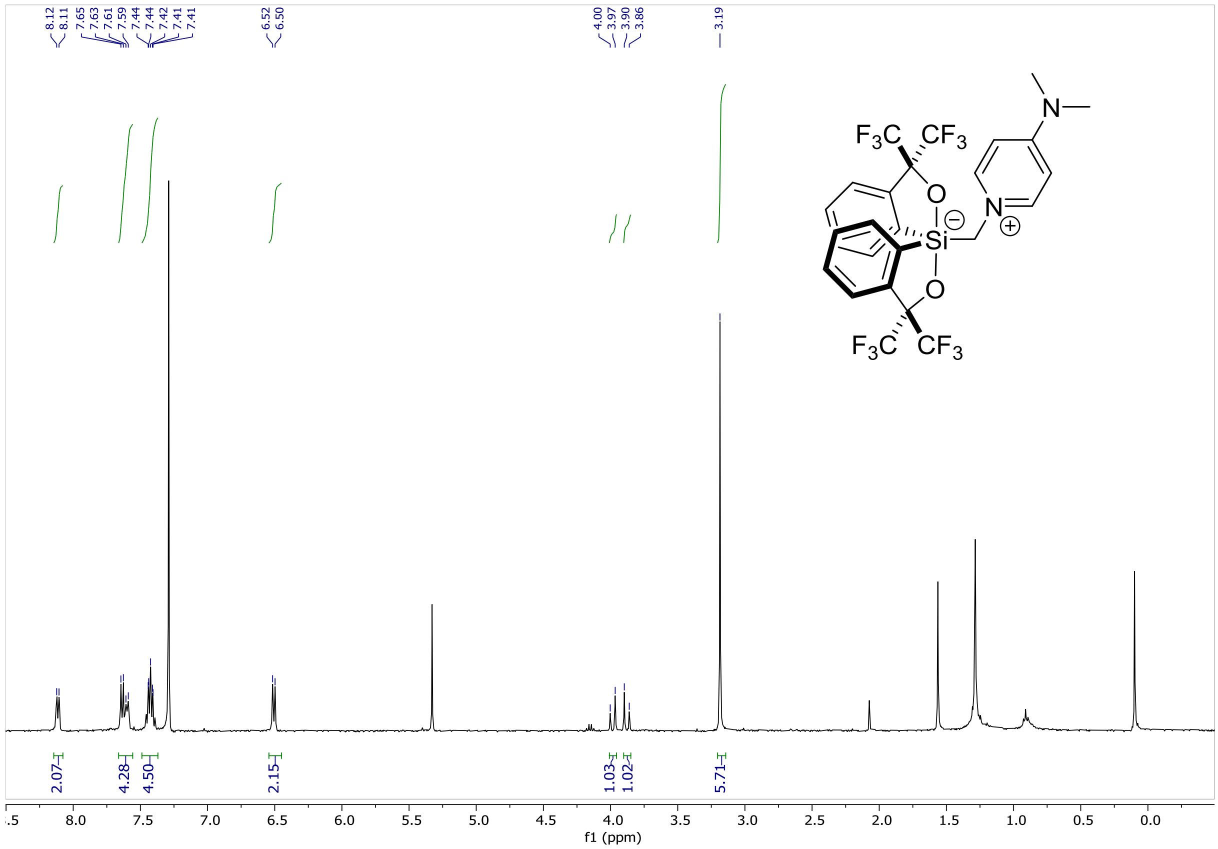

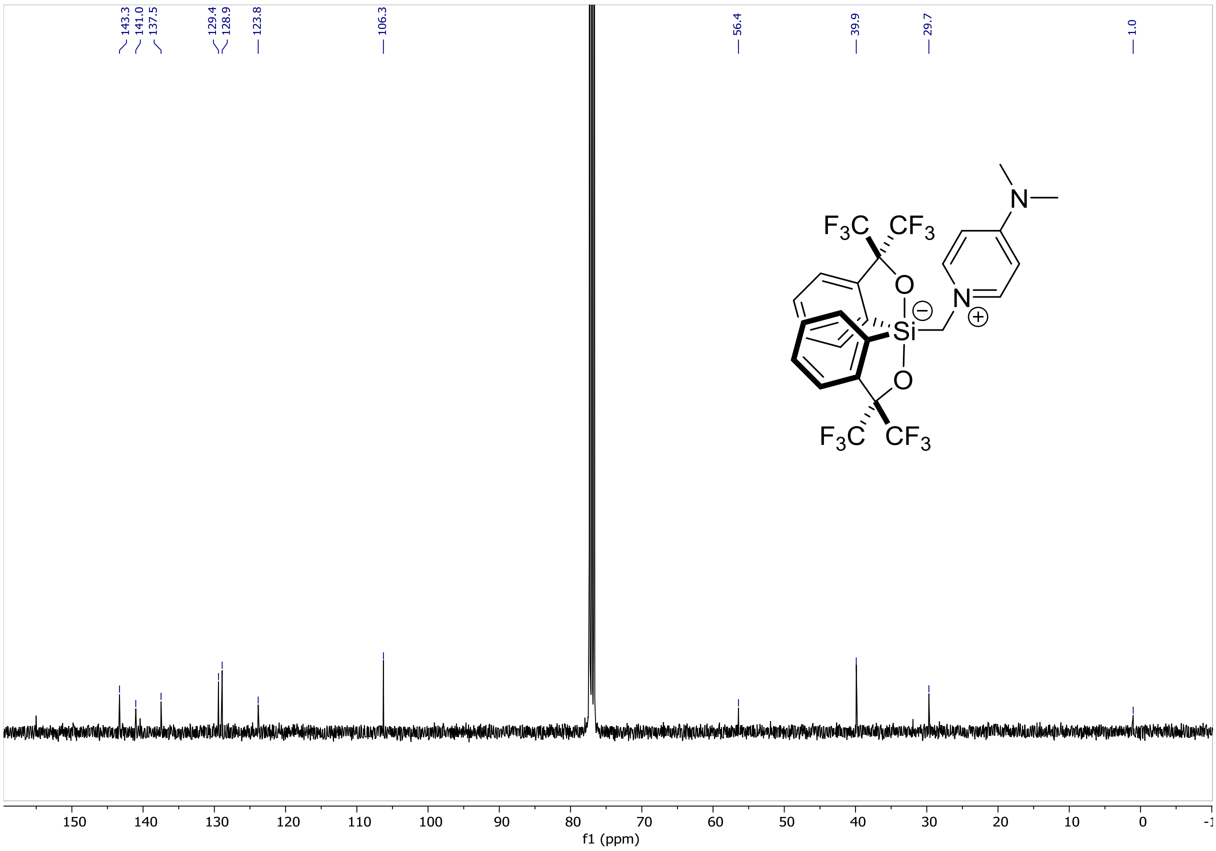

143.3
141.0
137.5
129.4
128.9
123.8
106.3
56.4
39.9
29.7
1.0
F3C CF3
O
Si
N
O
F3C CF3
150 140 130 120 110 100 90 80 70 60 50 40 30 20 10 0
f1 (ppm)

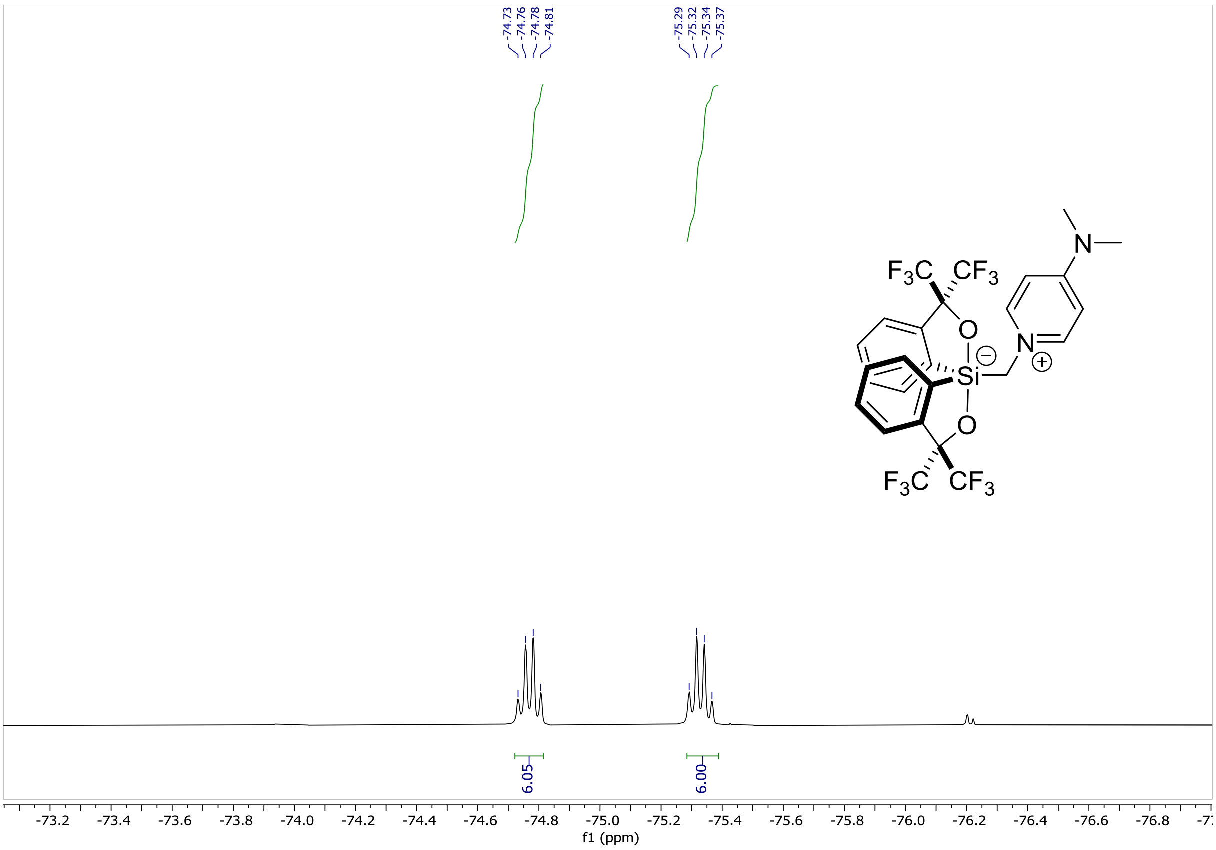

-74.73
-74.76
-74.78
-74.81
-75.29
-75.32
-75.34
-75.37
6.05
6.00
-73.2 -73.4 -73.6 -73.8 -74.0 -74.2 -74.4 -74.6 -74.8 -75.0 -75.2 -75.4 -75.6 -75.8 -76.0 -76.2 -76.4 -76.6 -76.8
f1 (ppm)

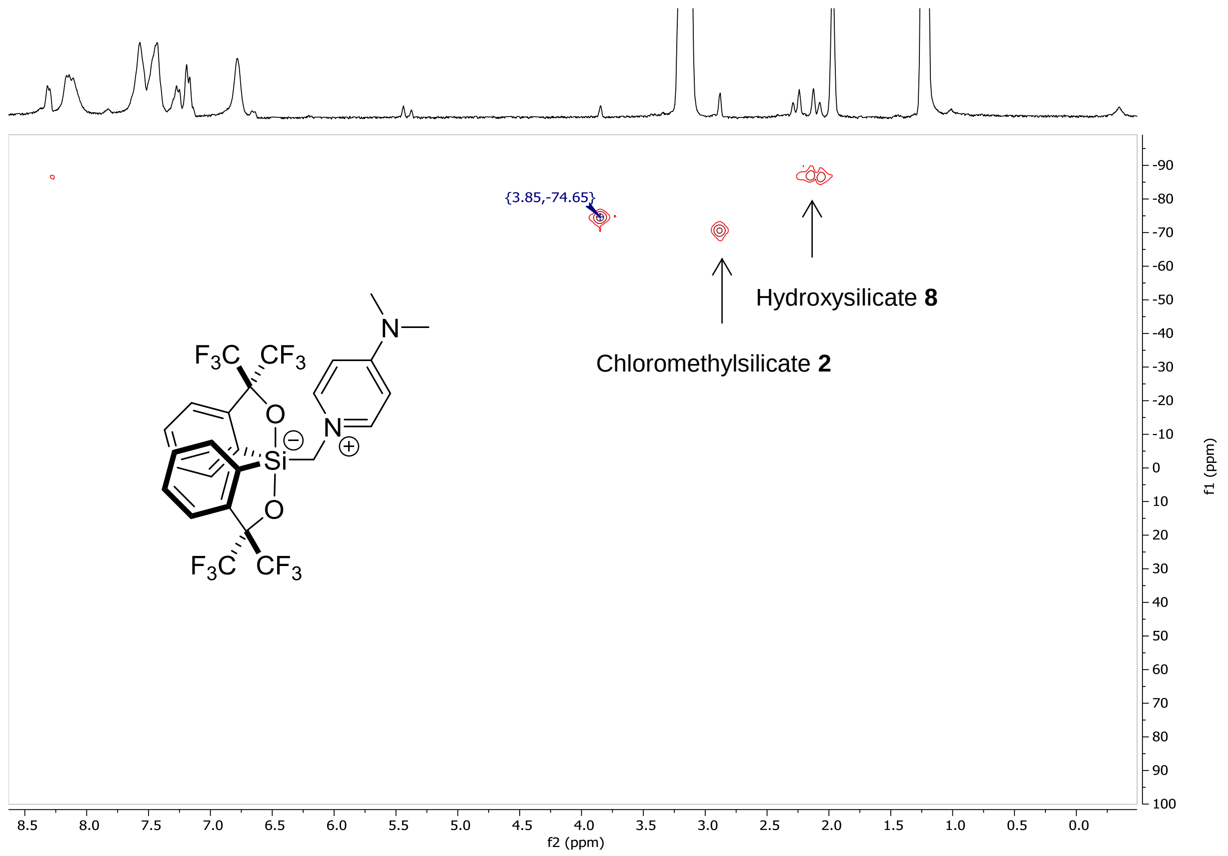
{3.85,-74.65}
Hydroxysilicate **8**
Chloromethylsilicate **2**
f2 (ppm)
f1 (ppm)

IV. Computational details

All DFT computations were carried out using the Gaussian 09[2] software package. Optimization of geometries and frequencies were performed using the B3LYP-D3 functional and the 6-311G(2d,p) basis set for all elements. The nature of all stationary points was confirmed by analyzing the harmonic vibrational frequencies.

In the following table is reported the energy (in Hartree) and the cartesian coordinates (x,y,z) of the computed species.

**A**<br>E(B3LYP-D3) = -2829.28571261<br>N_{imag} = 0				**TS$_{A}$□**<br>E(RB3LYP-D3) = -2829.23036311<br>N_{imag} = 1 (- 480.07 cm^{-1})			
Si	0.00392400	-0.15835100	-0.53838497	Si	-0.09187700	0.66518199	0.33781800
F	-2.25630593	2.06888199	1.46054304	F	-1.58827901	-2.47315907	-0.28454700
F	-4.31937981	1.40451503	1.34532106	F	-3.72134495	-2.29238200	-0.63906401
F	-3.46464109	2.58647895	-0.25899601	F	-2.95735502	-2.34927106	1.38887596
F	-4.91092014	-0.62417102	-0.35780901	F	-4.89466476	0.07042000	-0.07125700
F	-4.14080191	0.58106798	-1.98627806	F	-4.06961918	0.07789000	1.93416905
F	-3.31528711	-1.38634801	-1.61569798	F	-3.63379312	1.71224105	0.58025002
F	3.46918011	-1.63350904	1.77978802	F	2.67069411	-2.07649708	2.23759890
F	4.31524515	0.36123100	1.85441005	F	4.39877415	-1.07936001	1.39019895
F	2.25064611	0.03117100	2.43559289	F	2.89582992	0.06637400	2.45915103
F	3.34271789	-0.22693200	-2.17909408	F	1.77664900	-1.62037206	-1.82754695
F	4.13100100	-1.75804901	-0.86611003	F	1.82427895	-3.01099300	-0.16959800
F	4.92788696	0.25593501	-0.77783602	F	3.67634511	-2.23155499	-0.96894902
O	-1.61510396	0.66206700	-0.86781901	O	-1.49192095	-0.03663200	1.01272702
O	1.62007201	-0.92075598	-0.10940800	O	0.94085598	-0.63255900	0.64770001
C	-0.90520298	-1.06346798	0.85626298	C	-0.90324903	0.71778703	-1.35568798
C	-0.38710701	-1.99289095	1.76201499	C	-0.45701900	1.17342806	-2.59634089
H	0.65942299	-2.26101208	1.70488095	H	0.52970803	1.60867703	-2.68756509
C	-1.20204604	-2.56747603	2.73266792	C	-1.26888001	1.06215405	-3.71948099

H	-0.79062200	-3.28896689	3.42939091	H	-0.91506201	1.41929901	-4.68033695
C	-2.54883194	-2.21533394	2.81418705	C	-2.53391504	0.48688501	-3.61580396
H	-3.18284988	-2.66096306	3.57160211	H	-3.16452289	0.40005001	-4.49344778
C	-3.08297491	-1.28793001	1.92531395	C	-2.99253011	0.01787500	-2.39026403
H	-4.12631989	-1.01193202	1.99263597	H	-3.97145796	-0.43258700	-2.30670404
C	-2.25349402	-0.72540700	0.95813799	C	-2.17154002	0.13926800	-1.27349496
C	-2.68065596	0.31180200	-0.08378900	C	-2.52214909	-0.33518299	0.14094301
C	-3.19677305	1.60000205	0.61837298	C	-2.70697403	-1.87919700	0.15947001
C	-3.78045702	-0.27689701	-1.01156700	C	-3.79449797	0.38879201	0.66454703
C	0.91128701	1.50169098	-0.39488900	C	1.87176502	1.66472602	-0.34261501
C	0.39350599	2.78802299	-0.56742698	C	2.24128294	2.83765888	-1.00401497
H	-0.65388000	2.90820789	-0.81050903	H	1.56327796	3.68593693	-1.01041496
C	1.20940495	3.90613389	-0.42181301	C	3.46694493	2.95165610	-1.65010798
H	0.79814500	4.89986086	-0.55820698	H	3.73187900	3.87305307	-2.15834093
C	2.55641603	3.75240993	-0.09610000	C	4.36008406	1.88290501	-1.63139403
H	3.19049311	4.62327719	0.02103300	H	5.32391691	1.96556997	-2.12043095
C	3.09096909	2.48034096	0.08349000	C	4.01238680	0.70271301	-0.98741603
H	4.13467884	2.36252689	0.34069300	H	4.70338821	-0.12858599	-0.98320299
C	2.26105499	1.37259805	-0.06985600	C	2.76919293	0.59779298	-0.35957301
C	2.68550801	-0.08809800	0.10186700	C	2.27888894	-0.69413298	0.30015799
C	3.19719601	-0.33431000	1.55048704	C	3.06955409	-0.95949203	1.61185503
C	3.79017591	-0.45693901	-0.92829698	C	2.39570093	-1.90889096	-0.66962600
C	0.03146700	-0.95159698	-2.28536391	C	0.48256001	2.14557791	1.18496597
H	0.96577799	-0.73979700	-2.80015993	H	0.30694300	3.12300301	0.77124500
H	-0.81179601	-0.65355802	-2.90131497	H	1.23938203	2.07330203	1.94865894
Cl	-0.06167800	-2.79539108	-2.20849609	Cl	-0.99293298	2.91360092	2.95841599
**B**				**C**			
E(B3LYP-D3) = -2829.32130998				E(B3LYP-D3) = -8177.91061921			
N_{imag} = 0				N_{imag} = 0			
Si	0.17184500	0.51182997	0.49798000	Si	-0.83663702	0.24887000	-0.57611603
F	2.16070199	-0.82968497	-2.26371098	F	-3.84964204	1.63904798	0.96819699

F	4.29034424	-0.70401597	-1.86011195	F	-5.47745705	0.20154400	0.94044799
F	3.21918201	1.02674699	-2.60561991	F	-5.03522301	1.38430095	-0.82324600
F	5.08162785	0.74986500	0.29660800	F	-5.11330986	-2.10448003	-0.46335000
F	3.98757505	2.46694708	-0.44815800	F	-4.72816896	-0.91310602	-2.23206902
F	3.54266000	1.67595005	1.51619101	F	-3.24486995	-2.33337808	-1.54639804
F	-1.98506498	-2.07203007	-2.17789602	F	2.28286004	0.67971998	2.58458209
F	-3.73411298	-0.79756200	-2.31234908	F	1.92160904	2.81943393	2.54068995
F	-1.75274706	0.04564700	-2.55954099	F	0.25624499	1.44057405	2.48170304
F	-3.14507890	-1.40495396	1.76127994	F	2.98156595	2.02267098	-1.35300100
F	-2.29708195	-2.85232496	0.39956000	F	3.77221203	0.78181398	0.24919200
F	-4.26563311	-2.06329489	0.01685200	F	3.57567596	2.92264104	0.52027702
O	1.58916295	1.10112798	-0.37727600	O	-2.53550100	0.28589699	-1.08401406
O	-1.02641201	-0.65645999	0.02449800	O	1.15432894	0.31690100	0.06177900
C	1.29680002	-0.94303399	1.05012703	C	-1.35835099	-0.75831598	0.92422301
C	0.97363001	-2.03277206	1.86026704	C	-0.58978200	-1.33734703	1.93341506
H	-0.03332300	-2.12879300	2.24893689	H	0.48287001	-1.21133494	1.94750702
C	1.93217599	-2.98966098	2.16625404	C	-1.19164300	-2.10454702	2.92243195
H	1.67420006	-3.83486390	2.79606295	H	-0.57784802	-2.56176591	3.68951988
C	3.23077893	-2.87226605	1.66420400	C	-2.57202697	-2.29252100	2.92237091
H	3.97655106	-3.62201500	1.90471697	H	-3.04001307	-2.88671088	3.69917703
C	3.56917500	-1.79680705	0.85357302	C	-3.35525799	-1.73182201	1.92008698
H	4.57121277	-1.70178604	0.45730299	H	-4.42522621	-1.88634706	1.91131794
C	2.58971596	-0.84838998	0.56053001	C	-2.73675799	-0.98068899	0.92472100
C	2.77067900	0.38699901	-0.32051700	C	-3.45029998	-0.34616199	-0.26960400
C	3.11979389	-0.02741200	-1.77515602	C	-4.47099304	0.72461200	0.20498800
C	3.85626507	1.32979000	0.26010701	C	-4.15165520	-1.43174505	-1.13317502
C	-2.25369501	1.96201301	0.00732400	C	-0.59554201	2.11547589	-0.61644101
C	-2.88431001	3.16275096	0.35583299	C	-1.54318702	3.01095700	-1.12318397
H	-2.27651000	4.05805302	0.42144200	H	-2.46540594	2.62562490	-1.53665805
C	-4.24423790	3.23285389	0.61183202	C	-1.31482100	4.38114882	-1.08707297
H	-4.69881487	4.18069983	0.87897700	H	-2.05433297	5.06368780	-1.49050105
C	-5.01824522	2.08177900	0.51916897	C	-0.14359000	4.87768221	-0.52171701

H	-6.08586788	2.11590195	0.70285797	H	0.03367000	5.94640923	-0.48317200
C	-4.41150379	0.87506199	0.20097201	C	0.80211198	4.00471020	0.00169000
H	-5.02716208	-0.00917200	0.14269701	H	1.70409203	4.39314890	0.45320001
C	-3.03655791	0.79475200	-0.03510300	C	0.57282400	2.63263106	-0.06263100
C	-2.35778308	-0.55439597	-0.33137101	C	1.53792298	1.58570302	0.48343599
C	-2.46213794	-0.85640502	-1.85910201	C	1.51060200	1.63103294	2.04444599
C	-3.02635598	-1.73093104	0.46417001	C	2.97976494	1.84999704	-0.03189800
C	-0.79300600	1.95380497	-0.30727100	C	-0.20978600	-0.77837199	-2.05628395
H	-0.33536300	2.90469193	-0.03509800	H	0.65761203	-0.37387300	-2.57576990
H	-0.64706397	1.82154298	-1.38292003	H	-1.00690901	-1.00574696	-2.75780797
Cl	-0.15741500	1.22555900	2.56338692	Cl	0.34997499	-2.42057705	-1.41382694
				Mg	2.43017197	-1.24285698	-0.36106101
				Br	3.00629997	-2.66822290	1.52285099
				Br	3.60527992	-1.12789798	-2.51664090
**T□□□**				**D**			
E(B3LYP-D3) = -8177.86039854				E(B3LYP-D3) = -8177.93989166			
N_{imag} = 1 (- 407.47 cm^{-1})				N_{imag} = 0			
Si	0.61682600	0.39350700	0.76983601	Si	0.88539600	1.08174300	0.36461499
F	-0.60569602	1.07691300	-2.53246808	F	-1.65131903	1.09405005	-2.35750890
F	-2.21992612	2.52735209	-2.52512693	F	-3.36859488	2.21134901	-1.63648403
F	-2.62762904	0.44408500	-2.07433510	F	-3.10314202	0.14157800	-1.05607796
F	-3.19385600	3.31309295	-0.14822100	F	-3.36601901	2.98279190	0.97540897
F	-3.68399405	1.26273406	0.34654200	F	-3.14754605	0.90420598	1.55354702
F	-2.38320303	2.40214396	1.64729202	F	-1.67736399	2.36124706	2.19397902
F	2.13306689	-2.71144605	-1.86673796	F	2.65298510	-1.21065998	-2.60306311
F	4.13212013	-2.58503890	-1.03825700	F	4.48534489	-1.87440705	-1.67656803
F	2.44453096	-2.96658802	0.26661399	F	2.57555199	-2.62705302	-0.96719098
F	3.01458001	1.38133705	-1.53245294	F	3.87469697	1.84534097	-0.01349900
F	2.49931002	-0.21564700	-2.90175104	F	3.97957897	1.15225196	-2.06436110
F	4.48204994	-0.13239600	-2.03633308	F	5.37594700	0.39389199	-0.59100002
O	-1.05101204	0.44118199	0.14397199	O	-0.74116403	0.66692603	0.24835500

O	1.28601205	-0.49249899	-0.49019301	O	1.80442500	0.16968299	-0.64842999
C	0.74993700	2.24159503	0.52861398	C	0.58470702	2.80796504	-0.25799799
C	1.79376996	3.12103009	0.81706297	C	1.44105899	3.87604308	-0.52883798
H	2.65770197	2.77171111	1.36729503	H	2.51084399	3.76067996	-0.41256300
C	1.74113297	4.43875122	0.38091001	C	0.92101002	5.09077406	-0.95817101
H	2.55453897	5.11778688	0.60988599	H	1.58645594	5.91872692	-1.17411995
C	0.65371001	4.88454103	-0.36759999	C	-0.45552099	5.24837399	-1.11499703
H	0.62053603	5.90873814	-0.72061002	H	-0.85640597	6.19791985	-1.44989502
C	-0.39327499	4.02197218	-0.66701198	C	-1.32223105	4.19592905	-0.84739500
H	-1.23889399	4.36819601	-1.24320495	H	-2.38846707	4.31973410	-0.96874303
C	-0.33642000	2.70968390	-0.20606700	C	-0.78906399	2.98183203	-0.42383400
C	-1.44183505	1.67520499	-0.40322599	C	-1.60244298	1.73313200	-0.08426100
C	-1.73717999	1.42599702	-1.90573800	C	-2.44997406	1.27350998	-1.30048895
C	-2.70345592	2.16371393	0.36591700	C	-2.47842908	1.98567104	1.17352104
C	2.51470208	0.11053700	1.74380100	C	2.70548606	-0.18576799	2.13305402
C	3.09841704	0.57750702	2.92298293	C	3.01433301	-0.62933302	3.42241311
H	2.47447395	0.95811802	3.72489190	H	2.48079491	-0.18953300	4.25659609
C	4.47849607	0.57179302	3.09300494	C	3.94825101	-1.62261105	3.65308809
H	4.91416693	0.93792099	4.01616383	H	4.15202093	-1.95191598	4.66525412
C	5.29847479	0.08902300	2.07696009	C	4.60581589	-2.20226598	2.57481694
H	6.37459803	0.07527000	2.20446897	H	5.33804083	-2.98552608	2.72936296
C	4.74009085	-0.36790201	0.88958597	C	4.30835819	-1.78621495	1.28840005
H	5.37924385	-0.72524297	0.09490400	H	4.82688999	-2.25353408	0.46631899
C	3.35514903	-0.35088399	0.72872901	C	3.35542488	-0.79001701	1.04572105
C	2.64695811	-0.75560600	-0.56413001	C	3.07667208	-0.35856199	-0.40313599
C	2.82529092	-2.28120589	-0.81467599	C	3.18707800	-1.54343295	-1.42877698
C	3.17001390	0.06724700	-1.78092098	C	4.10110378	0.75460899	-0.79093701
C	0.45587200	-0.52257299	2.32156110	C	1.69232702	0.93597102	2.02115202
H	0.55714399	-0.02379300	3.27168012	H	2.18908811	1.89298606	2.22143602
H	0.65662098	-1.58720696	2.31592202	H	0.92196602	0.80441397	2.78212404
Cl	-1.85665298	-0.89545703	2.95967603	Cl	-0.07364000	-1.90419698	2.26703811
Mg	-2.36795592	-1.43615401	0.68897098	Mg	-1.59519100	-2.09191108	0.56842101

Br	-4.73013496	-1.73557699	0.14958400	Br	-3.92907310	-2.47486591	1.17945600
Br	-0.84726202	-3.21346307	0.01139300	Br	-0.80622900	-2.51796699	-1.69474804
**E**				**TS_{EG}**			
E(B3LYP-D3) = -2751.371693				E(B3LYP-D3) = -2751.29882958			
N_{imag} = 0				N_{imag} = 1 (- 533.0 cm^{-1})			
F	-1.64788306	-2.18193507	-2.61845899	F	-1.41407394	-2.08959603	-2.81126308
F	-3.74023104	-1.66302800	-2.35488391	F	-3.40464091	-2.54165792	-2.07536006
F	-2.23095393	-0.11779600	-2.35384297	F	-2.66212392	-0.51197100	-2.02083611
F	-2.67874193	-3.08262610	1.37007594	F	-0.97654802	-3.81010604	0.99933100
F	-2.16286802	-3.90998006	-0.56176800	F	-0.88635498	-4.17571306	-1.13065100
F	-4.14446783	-3.10347795	-0.21565400	F	-2.79811597	-4.17466307	-0.10395900
O	-0.96333802	-1.47587800	-0.09445800	O	-0.42399099	-1.52126396	-0.38568300
C	-2.18823695	0.45491600	1.05213296	C	-1.89545798	-0.40691301	1.41529703
C	-2.76353097	1.50070000	1.77725601	C	-2.60979605	0.29198501	2.39093304
H	-2.12616897	2.17098904	2.34536195	H	-2.09631205	1.01983202	3.00975490
C	-4.13998413	1.71330595	1.77008200	C	-3.97251797	0.07937300	2.56611705
H	-4.56846094	2.53183508	2.33884096	H	-4.51888990	0.63403898	3.32054305
C	-4.97228193	0.87880099	1.02408600	C	-4.63888407	-0.85151601	1.76918697
H	-6.04285192	1.04746699	1.00817597	H	-5.70137310	-1.01824903	1.90096402
C	-4.42514181	-0.17676499	0.29970199	C	-3.94305801	-1.56885195	0.79996598
H	-5.07025099	-0.82530397	-0.27877101	H	-4.46527576	-2.28288889	0.17710800
C	-3.04649210	-0.38062999	0.33803999	C	-2.57719803	-1.34859002	0.64383799
C	-2.30070901	-1.50473201	-0.39141899	C	-1.69966197	-2.01956797	-0.41432500
C	-2.48236609	-1.36517000	-1.93974698	C	-2.30011010	-1.78782403	-1.84363306
C	-2.83331609	-2.90628505	0.04372400	C	-1.60009801	-3.55813503	-0.16614500
C	-0.52861702	-1.01101696	2.60287094	C	0.03226100	-1.08766305	2.24511194
H	-1.32233703	-1.74177694	2.50827098	H	-0.24404500	-2.13129210	2.27350092
H	-0.82376200	-0.30307701	3.37592506	H	-0.16971000	-0.58602500	3.18125391
Cl	0.82440501	-2.00736189	3.44535398	Cl	2.20883203	-1.69732404	3.22843790
C	1.74049699	-1.64665902	-0.65516502	C	2.30648494	-1.62925506	-0.30445200
C	2.61815596	-0.66846901	1.21706200	C	2.81387210	0.56006902	0.14938000

C	2.96510196	-2.14482808	-1.03080702	C	3.61300111	-1.92047906	-0.60352802
H	0.86485797	-1.82198596	-1.25995696	H	1.55079401	-2.39562988	-0.35538900
C	3.88464093	-1.11682296	0.92477202	C	4.14438581	0.36172199	-0.12090400
H	2.45541191	-0.06785200	2.09787798	H	2.47295094	1.53412497	0.45818999
C	4.11019802	-1.89161003	-0.23695800	C	4.60745192	-0.91516900	-0.52088201
H	3.02470994	-2.71811199	-1.94327497	H	3.85385203	-2.93337297	-0.88742697
H	4.68653488	-0.85786003	1.59897995	H	4.81349277	1.20181596	-0.01789800
N	1.53700197	-0.91692901	0.45913199	N	1.87313795	-0.40160599	0.06044000
N	5.33937502	-2.35582995	-0.57055700	N	5.90777779	-1.15814996	-0.79799497
C	6.49158907	-2.05070400	0.26953799	C	6.89657116	-0.09071200	-0.68319499
H	6.66610622	-0.97243702	0.33219099	H	6.66427422	0.73870599	-1.35657299
H	6.35821295	-2.43933797	1.28305197	H	6.94921112	0.29428300	0.33904099
H	7.37663794	-2.51392388	-0.15721200	H	7.87405109	-0.48135501	-0.95057899
C	5.52651215	-3.13251996	-1.79064202	C	6.33997583	-2.49605489	-1.19027197
H	6.57207012	-3.41477799	-1.87603903	H	7.41063404	-2.48448110	-1.37240100
H	4.92722082	-4.04718685	-1.77579904	H	6.13399410	-3.22695494	-0.40340900
H	5.25460911	-2.55406189	-2.67848611	H	5.84076691	-2.82036901	-2.10717106
O	-0.08446800	0.78635597	-0.76256001	O	-0.46463200	0.88559401	-0.81630802
C	0.51966703	1.56005096	1.59458196	C	0.31110600	1.64221203	1.46996403
C	0.81796700	1.89469695	2.91989994	C	0.81304902	1.93961298	2.74142694
C	0.80149299	2.51837397	0.62196398	C	-0.05250000	2.71328092	0.65717101
C	1.37146199	3.12904906	3.25317001	C	0.93009102	3.25658393	3.17783809
H	0.63102502	1.18192303	3.71508503	H	1.13988101	1.13757002	3.39328289
C	1.35869205	3.75858307	0.92931098	C	0.05264300	4.03920078	1.07362199
C	1.64371896	4.06408119	2.25647998	C	0.54575598	4.30776787	2.34599090
H	1.59521198	3.36262608	4.28850079	H	1.32512999	3.46809793	4.16539001
H	1.57170105	4.48016310	0.15197501	H	-0.23777300	4.85300016	0.42313200
H	2.07710600	5.02448606	2.51029301	H	0.63629198	5.33313513	2.68469596
C	0.44346100	2.04534698	-0.79096699	C	-0.54040098	2.25316000	-0.71824402
C	-0.61379802	2.99375200	-1.43680406	C	-2.02060199	2.68505311	-0.94846803
C	1.71883500	2.01985002	-1.68894196	C	0.34565899	2.85122490	-1.85030401
F	2.70154595	1.31927800	-1.09596002	F	1.64257300	2.56133199	-1.63297403

F	2.22322202	3.25171304	-1.94728601	F	0.25541601	4.19655800	-1.94566500
F	1.49027395	1.43812096	-2.88397694	F	0.03444000	2.35107899	-3.06132197
F	-0.19532700	4.27776194	-1.55791903	F	-2.19473004	4.02680492	-0.88072503
F	-0.97257501	2.59116006	-2.67349410	F	-2.49272203	2.29641390	-2.14835596
F	-1.73098397	3.02276802	-0.69169402	F	-2.81436610	2.14162588	-0.01362600
Si	-0.31277600	-0.05432400	0.86746699	Si	-0.01074500	-0.08784700	0.63993299
**F**				**TS_{FG}**			
E(B3LYP-D3) = -3211.72472818				E(B3LYP-D3) = -3211.70507892			
N_{imag} = 0				N_{imag} = 1 (- 362.57 cm^{-1})			
Si	-0.55892599	0.35692400	-0.46548399	Si	0.58620697	0.00677000	0.47071099
F	-3.92801905	0.72584701	1.04016495	F	0.33209899	2.31428003	-2.25129104
F	-5.01607895	-1.15183604	1.03542197	F	-1.33989596	3.67236710	-1.98527801
F	-4.96934605	0.08389000	-0.74625701	F	-1.66991401	1.68465900	-2.78579092
F	-3.96424603	-3.22595811	-0.36395901	F	-3.09633994	3.13450909	0.00960300
F	-3.91196203	-2.00219893	-2.15104604	F	-3.40906596	1.13425803	-0.76491898
F	-2.08827305	-2.90164804	-1.40653706	F	-2.70813990	1.40840805	1.26617706
F	2.55808711	1.84879303	2.23731089	F	2.48173499	-2.25154901	-2.70134997
F	1.85781097	3.88440490	1.97124398	F	4.03288603	-2.94888210	-1.35518301
F	0.45197901	2.30346894	2.45509791	F	1.94101596	-3.15835190	-0.81108499
F	2.28442788	2.39809608	-1.92081201	F	3.82321596	1.19002903	-0.73733300
F	3.61938906	1.93517995	-0.27930701	F	3.35807705	0.31265700	-2.66047812
F	2.87823296	3.95608211	-0.53185701	F	5.04960299	-0.42038700	-1.52697802
O	-2.24179912	-0.15675101	-0.98427403	O	-0.76737303	0.47845101	-0.49242100
O	1.09086502	0.93098700	0.11067100	O	1.55977702	-0.43615401	-0.83978701
C	-0.82501900	-0.75419801	1.04223502	C	0.72843599	1.74496901	1.14644301
C	0.05589000	-1.00489104	2.09694290	C	1.61898303	2.31170702	2.05926609
H	1.01208496	-0.49934700	2.12101293	H	2.37274694	1.69757104	2.53464103
C	-0.28832400	-1.89622700	3.10811305	C	1.54595006	3.66772699	2.36014891
H	0.40194201	-2.08821011	3.92149806	H	2.23898506	4.10234880	3.07052398
C	-1.52207804	-2.54537201	3.07857895	C	0.58588099	4.47127819	1.74744403
H	-1.79004395	-3.23872304	3.86691499	H	0.53261298	5.52720594	1.98309398

C	-2.41529608	-2.30570889	2.03890300	C	-0.30652201	3.92626691	0.82983601
H	-3.37257195	-2.80806303	2.02063489	H	-1.04760504	4.55464792	0.35598901
C	-2.05296397	-1.41430700	1.03214002	C	-0.22430700	2.56785297	0.53952301
C	-2.91261792	-1.04070401	-0.17885900	C	-1.12276804	1.81733894	-0.44706500
C	-4.22571278	-0.35028401	0.28891900	C	-0.95943499	2.38381410	-1.88382804
C	-3.23900604	-2.30187201	-1.02635002	C	-2.60514498	1.87830198	0.01154300
C	-0.97512400	2.19511294	-0.65148699	C	2.17515492	-0.90613103	1.68811500
C	-2.16640806	2.76862597	-1.10428500	C	2.46987510	-0.94643801	3.05160093
H	-2.98379588	2.12628508	-1.40348804	H	1.68594694	-0.77008998	3.78045893
C	-2.30797791	4.15179777	-1.16348803	C	3.75931811	-1.21391499	3.49935889
H	-3.23460388	4.58758116	-1.51904297	H	3.97160006	-1.23977804	4.56176376
C	-1.26202404	4.98115396	-0.76085502	C	4.77544117	-1.45774996	2.57843804
H	-1.37506294	6.05797100	-0.80223799	H	5.77984715	-1.67644298	2.91956401
C	-0.07086600	4.42989111	-0.29955801	C	4.50694084	-1.41052103	1.21533597
H	0.73705298	5.07378912	0.01941100	H	5.30556488	-1.58200300	0.50743699
C	0.05687700	3.04418206	-0.25500101	C	3.21202707	-1.12860596	0.77993798
C	1.28984499	2.28158307	0.23296700	C	2.83659506	-0.96613598	-0.69252998
C	1.55230904	2.59042311	1.73481905	C	2.83259106	-2.34467292	-1.40796900
C	2.53396702	2.65524912	-0.61889899	C	3.78567791	0.03158700	-1.41950500
C	0.26188299	-0.56953698	-1.95499599	C	0.13914800	-1.46646297	1.41408801
H	0.59506398	0.15155999	-2.70048904	H	-0.06306300	-1.41154397	2.47287989
H	-0.44903401	-1.24057305	-2.43059492	H	0.42541400	-2.43939805	1.04408896
C	1.25959098	-2.55962801	-0.92624801	C	-2.87773108	-1.64144194	1.93544900
C	2.69228005	-0.89087802	-1.68928301	C	-2.26767802	-2.44260502	-0.11757300
C	2.30470800	-3.29262209	-0.44401601	C	-4.21839905	-1.68920803	1.62001097
H	0.23920099	-2.89636707	-0.82531101	H	-2.56206799	-1.27802098	2.90778303
C	3.78646588	-1.56672001	-1.22968304	C	-3.57689404	-2.52990699	-0.53690302
H	2.78463697	0.06238200	-2.18390298	H	-1.46173298	-2.72031498	-0.78857797
C	3.63803911	-2.81569099	-0.56956398	C	-4.62108803	-2.13905501	0.33855200
H	2.08016706	-4.23206615	0.03499600	H	-4.93585014	-1.36588097	2.35885692
H	4.75424290	-1.11689401	-1.38241994	H	-3.77831602	-2.88397694	-1.53652000
N	1.43580401	-1.36948597	-1.54963195	N	-1.90490305	-2.01340389	1.09674501

N	4.69065189	-3.50395107	-0.09450900	N	-5.92573690	-2.18572497	-0.03100000
C	6.04665422	-2.97771311	-0.24495600	C	-6.29591799	-2.64014912	-1.36609101
H	6.15815115	-2.02225089	0.27373099	H	-5.88037682	-1.98917305	-2.14139390
H	6.74977589	-3.68449998	0.18451101	H	-5.94873810	-3.66093493	-1.54787302
H	6.29950285	-2.83968306	-1.29883802	H	-7.37831402	-2.63079596	-1.45771801
C	4.49206877	-4.78103924	0.58982098	C	-6.96463680	-1.72717404	0.88340902
H	4.03303289	-5.51667118	-0.07511300	H	-6.82291317	-0.67653000	1.15382802
H	5.45606899	-5.16420603	0.90963697	H	-7.93272495	-1.82587504	0.40056399
H	3.86045790	-4.65963602	1.47302997	H	-6.97911882	-2.32226396	1.80104101
G<br>E(B3LYP-D3) = -2751.38585347<br>N_{imag} = 0				DMAP<br>E(B3LYP-D3) = -382.391238753<br>N_{imag} = 0			
F	-2.53487897	1.67243302	2.50695109	N	2.66828704	0.00000000	0.00011300
F	-4.50376177	1.28760302	1.71836603	C	1.95030999	-1.13352501	0.00005700
F	-3.18167806	-0.36710000	2.18209696	C	1.95030999	1.13352501	0.00004200
F	-2.40117002	2.33930993	-1.66868305	H	2.51832795	2.05939507	0.00012500
F	-2.58853102	3.31040692	0.25994399	C	0.56881601	-1.19673204	-0.00008800
F	-4.31310797	2.36834908	-0.66106802	H	0.09239200	-2.16586995	-0.00007900
O	-1.22504997	0.91387600	0.42065799	C	0.56881601	1.19673204	-0.00010500
C	-2.28837991	-0.85995901	-1.58365202	N	-1.54713905	0.00000000	-0.00051700
C	-2.78701401	-1.93515801	-2.32846808	C	-2.27978492	-1.25865901	0.00018600
H	-2.13272095	-2.41416311	-3.04721904	H	-2.05037999	-1.85628998	0.88786697
C	-4.08798981	-2.39005399	-2.17405891	H	-3.34634995	-1.05113006	-0.00044400
H	-4.44519615	-3.22305608	-2.76782489	H	-2.04965091	-1.85757399	-0.88641500
C	-4.92872477	-1.76508296	-1.25941098	C	-2.27978611	1.25865805	0.00021600
H	-5.95179081	-2.09705591	-1.13313901	H	-3.34635091	1.05113006	-0.00033800
C	-4.44765806	-0.70943999	-0.49774900	H	-2.05032301	1.85629904	0.88787597
H	-5.11767721	-0.23986700	0.20644499	H	-2.04971004	1.85756505	-0.88640702
C	-3.13037801	-0.25991800	-0.63389403	C	-0.18576799	0.00000000	-0.00023800
C	-2.58984590	0.89379102	0.22583200	H	0.09239200	2.16586995	-0.00011400
C	-3.21805406	0.87547803	1.66836596	H	2.51832795	-2.05939507	0.00015500

C	-2.98972297	2.24388003	-0.45656899	
C	-0.87974501	-0.39520600	-1.83384001	
C	0.77175802	2.57787395	-0.87409300	
C	2.38015389	1.42773402	0.33192399	
C	1.53888404	3.70784497	-0.90448600	
H	-0.20013399	2.56703591	-1.33753800	
C	3.20104909	2.51843095	0.36497101	
C	2.81303096	3.72736001	-0.27430901	
H	1.14494705	4.57385588	-1.41232598	
H	4.14275122	2.42771506	0.88264900	
N	1.16987205	1.42844796	-0.27768299	
N	3.59829998	4.81703520	-0.28107399	
C	4.89854908	4.79635096	0.38862801	
H	4.78752804	4.59108877	1.45582902	
H	5.55472183	4.04125977	-0.05068400	
H	5.37020302	5.76749182	0.27533501	
C	3.16568089	6.03784323	-0.96076000	
H	3.94475007	6.78833580	-0.86980498	
H	2.98882198	5.85290813	-2.02277207	
H	2.25115895	6.43470287	-0.51344103	
O	1.53627503	-1.06311500	-0.90797299	
C	0.36614600	-1.11953700	1.32447600	
C	-0.18928599	-0.91358501	2.58835411	
C	1.26277101	-2.17750812	1.16288400	
C	0.12665799	-1.75949502	3.64708400	
H	-0.85672498	-0.08196600	2.75094509	
C	1.56650805	-3.05000806	2.20221591	
C	0.99088597	-2.83462501	3.45063090	
H	-0.30142999	-1.58314204	4.62687922	
H	2.24833488	-3.87612796	2.05472302	
H	1.22738898	-3.49954391	4.27262402	
C	1.85764098	-2.22252297	-0.24387400	

C	3.40775108	-2.33476806	-0.20877200	
C	1.26012397	-3.43163610	-1.02676797	
F	-0.07981600	-3.30671406	-1.09225702	
F	1.51833403	-4.62031794	-0.44517699	
F	1.71322703	-3.49849200	-2.29237390	
F	3.84624100	-3.51570511	0.27493700	
F	3.94871402	-2.18133998	-1.42886198	
Si	0.10534700	-0.10553300	-0.24852900	
H	-0.90334702	0.56852901	-2.35298800	
H	-0.36754200	-1.09463406	-2.48811889	
H	2.67267203	0.50537997	0.80492002	
F	3.92939305	-1.37112606	0.57799000	

V. Experimental references

[1] Frisch, M.J.; Trucks, G.W.; Schlegel, H.B.; Scuseria, G.E.; Robb, M.A.; Cheeseman, J.R.; Scalmani, G.; Barone, V.; Mennucci, B.; Petersson, G.A.; et al. *Gaussian 09, Revision D.01*; Gaussian, Inc.: Wallingford, CT, USA, 2009.
